# Deficient extravillous trophoblast invasion caused by impaired sialylation–Siglec-7 interaction contributes to recurrent pregnancy loss

**DOI:** 10.1038/s41419-026-08503-9

**Published:** 2026-03-02

**Authors:** Linyu Zhang, Ying Feng, Peng Wu, Liuyan Chen, Nan Jiang, Xue Ma, Qianhong Ma, Hao-Jie Lu, Xue Xiao, Fang Ma

**Affiliations:** 1 Center for Translational Medicine, Key Laboratory of Birth Defects and Related Diseases of Women and Children (Sichuan University), Ministry of Education, West China Second University Hospital, Sichuan University, Chengdu, Sichuan PR China; 2 Department of Obstetrics and Gynecology, West China Second Hospital, Sichuan University, Chengdu, Sichuan PR China; 3 West China School of Basic Medical Sciences & Forensic Medicine, Sichuan University, Chengdu, Sichuan PR China; 4 Institutes of Biomedical Sciences, Laboratory of Glycoconjugates Research, Fudan University, Shanghai, PR China; 5 Department of Pediatric Surgery, West China Hospital, Sichuan University, Chengdu, Sichuan PR China; 6 Key Laboratory of Birth Defects and Related Diseases of Women and Children (Sichuan University), Ministry of Education, West China Second University Hospital, Sichuan University, Chengdu, Sichuan PR China; 7 Institutes of Biomedical Sciences, Department of Chemistry and Laboratory of Glycoconjugates Research, Fudan University, Shanghai, PR China

**Keywords:** Glycobiology, Cytokines, Developmental biology, Diseases

## Abstract

Successful pregnancy requires precise immune interactions between fetal extravillous trophoblasts (EVT) and maternal decidual immune cells at the maternal–fetal interface. Glycosylation, particularly terminal sialylation, is emerging as a key modulator of these interactions; however, its functional role in regulating the EVT–immune crosstalk remains poorly defined. Here, we aimed to identify a critical sialic acid–Siglec-7–IL-8–STAT3 signaling axis that promotes EVT invasiveness and is disrupted during recurrent pregnancy loss (RPL). Using primary human tissues and organ-on-chip models, we demonstrate that EVTs from patients with RPL exhibit reduced sialylation, coinciding with an increased proportion of Siglec-7⁺ decidual natural killer (dNK) cells. Mechanistically, sialylated glycoproteins on EVT surfaces engage Siglec-7, stimulating IL-8 secretion by dNK cells, which, in turn, activates STAT3 in EVTs to enhance migration and invasion. Restoration of EVT sialylation re-engages Siglec-7, rescues IL-8–STAT3 signaling, and restores invasive capacity. Our findings reveal that defective EVT sialylation disrupts a key immunological checkpoint that normally promotes EVT invasion and potentially contributes to RPL. This work provides direct mechanistic evidence that specific glycan-encoded immune signals at the maternal-fetal interface are critical for healthy pregnancy outcomes and suggests that modulating sialylation may offer a therapeutic strategy for RPL.

**Figure:**
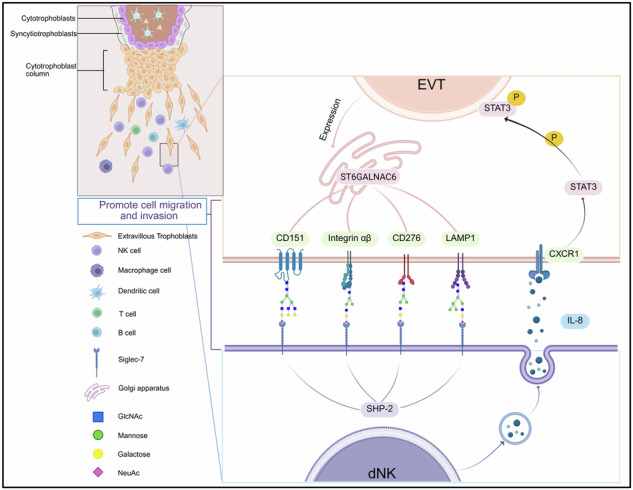
Proposed model of sialic acid–Siglec-7–mediated regulation of EVT invasion through the ST6GALNAC6–sialic acid–Siglec-7–IL-8–STAT3 signaling axis. Schematic representation of the working model: enhanced sialylation of EVT membrane glycoproteins—driven by ST6GALNAC6—facilitates recognition by Siglec-7 expressed on dNK cells. This interaction promotes the activation of the IL-8–STAT3 signaling pathway, which supports EVT cell migration and invasion. Disruption of sialylation or Siglec-7 engagement impairs this pathway and reduces EVT invasiveness, potentially contributing to the pathogenesis of RPL. Figure created with BioRender.com (https://BioRender.com/dxxt5az).

Proposed model of sialic acid–Siglec-7–mediated regulation of EVT invasion through the ST6GALNAC6–sialic acid–Siglec-7–IL-8–STAT3 signaling axis. Schematic representation of the working model: enhanced sialylation of EVT membrane glycoproteins—driven by ST6GALNAC6—facilitates recognition by Siglec-7 expressed on dNK cells. This interaction promotes the activation of the IL-8–STAT3 signaling pathway, which supports EVT cell migration and invasion. Disruption of sialylation or Siglec-7 engagement impairs this pathway and reduces EVT invasiveness, potentially contributing to the pathogenesis of RPL. Figure created with BioRender.com (https://BioRender.com/dxxt5az).

## Introduction

The establishment of a functional maternal–fetal interface depends on highly orchestrated interactions between fetal extravillous trophoblast (EVT) cells and maternal decidual immune cells during the first weeks of gestation [1]. Although immunological crosstalk has long been recognized as a determinant of EVT behavior, the molecular signals that calibrate this dialog remain unclear.

Protein glycosylation, particularly terminal sialylation, is a pivotal regulator of implantation, placentation, and maternal tolerance [2]. Glycans decorate virtually every cell type at the interface and are sensed by a dedicated repertoire of lectin-like receptors, including selectins, galectins, and sialic acid-binding immunoglobulin-like lectins (Siglecs) [3]. Recent reviews have highlighted that most reproductive glycobiology studies have been descriptive, leaving the functional consequences of specific glycan patterns largely unexplored [2, 4, 5]. One outstanding question is whether altered sialylation of EVT surface proteins and their recognition by inhibitory Siglecs on decidual natural killer (dNK) cells constitute an active checkpoint that safeguards pregnancy.

Siglec-7 is highly expressed in a subset of NK cells, and tumor studies have shown that the engagement of this receptor by sialylated ligands dampens cytotoxic responses [6]. Analogous glycan-based mechanisms have been proposed during early human pregnancy; however, direct causal evidence is lacking [7]. Of particular interest is interleukin-8 (IL-8), a dNK-derived chemokine that promotes EVT motility via STAT3 signaling [8]. Dysregulated IL-8 production has been documented in RPL, but the upstream trigger remains obscure [9]. These observations raise the possibility that a sialic acid–Siglec-7 sensor couples innate immune recognition to IL-8–STAT3–driven trophoblast invasion and that failure of this axis may underpin idiopathic RPL.

In this study, we combined transcriptomic, glycoproteomic, and functional analyses of primary human tissues to test this hypothesis. We report that EVTs from RPLs display a marked loss of α 2,6-linked sialic acid, which coincides with the expansion of Siglec-7^+^ dNK cells. We further demonstrated that restoring EVT sialylation re-engages Siglec-7, rescues IL-8–STAT3 signaling, and reinstates trophoblast invasiveness. By delineating a previously unrecognized ST6GALNAC6–sialic acid–Siglec-7–IL-8–STAT3 circuit, our study provides mechanistic insights into glycan-encoded immune regulation at the maternal–fetal interface and identifies a tractable therapeutic target for RPL.

## Results

### Single–cell transcriptomic profiling reveals altered sialylation and Siglec-7–associated immune activation at the maternal–fetal interface in RPL

**Fig. 1:**
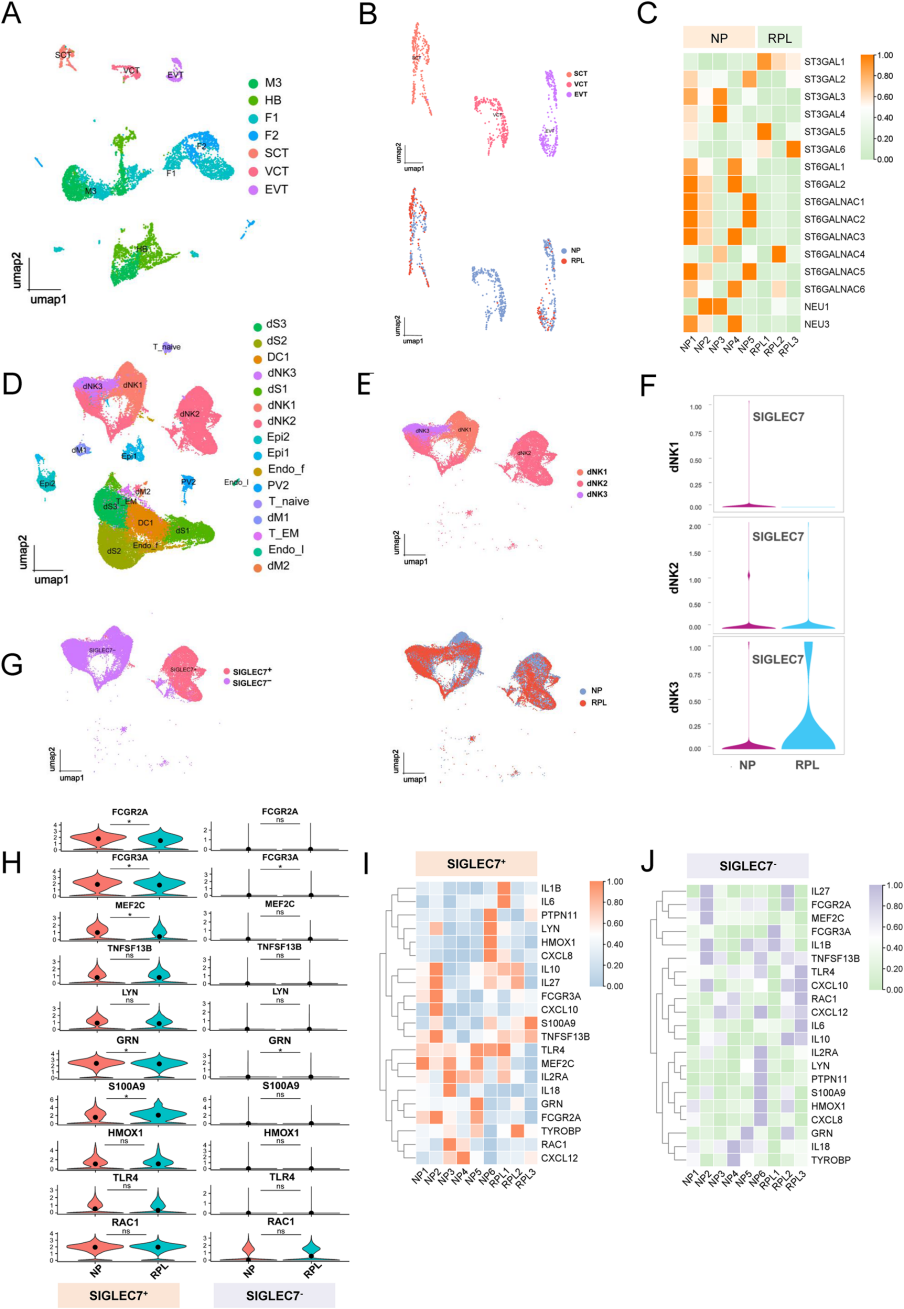
Dysregulated expression of sialylation-related genes in EVT cells and imbalance of Siglec7^+^ dNK cells in RPL. **A** UMAP visualization of fetal-derived cells. **B** UMAP visualization of trophoblast subpopulations based on 10×Genomics scRNA-seq data, identifying SCT, VCT, and EVT clusters. Cell types or states are indicated by color. *N* = *8 total; n* = *5 normal pregnancies (NP), n* = *3 recurrent pregnancies lost (RPL)*. **C** Heatmap showing the expression levels of sialylation-associated genes, including *ST3GAL1–6*, *ST6GAL1–2*, *ST6GALNAC1–6*, *NEU1* and *NEU3*, in EVT cells from NP and RPL samples. **D** UMAP visualization of maternal-derived cells. **E** UMAP of dNK cell subtypes on the basis of scRNA-seq data, identifying dNK1, dNK2, and dNK3 populations. The cell types are color-coded. *N* = *9 total; n* = *6 NP, n* = *3 RPL*. **F** Violin plots showing *SIGLEC7* expression across dNK subtypes in NP and RPL samples. **G** Left: UMAP plot indicating the distribution of *SIGLEC7*^+^ and *SIGLEC7*^−^ dNK cells among total dNKs. Right: UMAP plot comparing the proportions of *SIGLEC7*^+^ and *SIGLEC7*^−^ dNK cells between the NP and RPL samples, revealing a marked increase in the RPL samples. **H** Violin plots illustrating the differential expression of representative immune regulatory genes (e.g., *FCGR3A*, *S100A9*, *HMOX1*, and *TLR4*) between *SIGLEC7*^+^ and *SIGLEC7*^−^ dNK cells in the NP and RPL samples. Heatmaps highlighting distinct transcriptomic profiles of *SIGLEC7*^+^ (**I**) and *SIGLEC7*^−^ (**J**) dNK cells between the NP and RPL. *SIGLEC7*^*+*^ dNK cells in RPL are enriched in proinflammatory and EVT*-*suppressive genes (e.g., *IL1B*, *LYN*, *HMOX1*, and *CXCL8*). Box plots: the centerline represents the median; boxes span the interquartile range (IQR), from the 25th to 75th percentiles; whiskers extend to the most extreme values within 1.5× IQR; data points beyond this range are shown as outliers. *P < 0.05, ns not significant. M macrophage, HB Hofbauer, F fibroblast, SCT syncytiotrophoblast, VCT villous cytotrophoblast, EVT extravillous trophoblast, dS decidual stromal, DC dendritic cell, dNK decidua natural killer, Epi epithelial, Endo_f endometrial fibroblast, PV perivascular, dM decidual macrophages, Endo_l endometrial Lymphocytes.

To investigate novel cellular interactions distinct from well-characterised maternal-fetal communication, we conducted single-cell RNA sequencing (scRNA-seq) of chorionic villi–decidua pairs obtained from women with RPL (*n* = 3) and NP (*n* = 6). Unsupervised clustering and UMAP visualization revealed distinct fetal cell populations, including three trophoblast subtypes defined by canonical markers: syncytiotrophoblasts (SCTs; *EGFR*), villous cytotrophoblasts (VCTs; *NRP2*), and EVTs; *HLA-G* (Fig. 1A, B). Differential expression analysis between NP and RPL samples revealed consistent downregulation of most sialyltransferase and sialidase genes in EVTs from RPL tissues, highlighting the general suppression of sialylation–related pathways (Fig. 1C).

Parallel analysis of maternal decidual cells revealed multiple dNK cell subsets (Fig. 1D, E). On the basis of gene signatures, dNK1 cells were characterized by *ENTPD1*, *CYP26A1*, and *B4GALNT1* expression; dNK2 cells, by *ITGB2* and *ANXA1*; and dNK3 cells, by *KIR2DL1*, *ITGAE*, and *CD160* (Fig. 1E). Notably, the expression of *SIGLEC7*, which encodes an inhibitory receptor prominently expressed on NK cells, was markedly increased in dNK3 cells from RPL tissues and modestly elevated in dNK2 cells compared with that in NP controls (Fig. 1F). Although the overall expression of *SIGLEC7* remained low, its upregulation in RPL-associated dNK cell subsets may reflect a compensatory response aimed at modulating NK cell activity.

To investigate the immunological consequences of *SIGLEC7* expression, we further stratified the dNK1-3 populations into the *SIGLEC7*⁺ dNK and *SIGLEC7*⁻ dNK subsets (Fig. 1G). Differential gene expression analysis revealed that, in RPL tissues, *SIGLEC7*^+^ dNK cells exhibited a markedly proinflammatory transcriptional program compared with their *SIGLEC7*^−^ counterparts. Specifically, *SIGLEC7*⁺ cells presented significant downregulation of immunoregulatory markers in RPL, including *FCGR2A*, *FCGR3A*, *MEF2C*, and *GRN* (*P* < 0.05), while immunoregulatory genes such as *S100A9* were concurrently upregulated (Fig. 1H, I). This pronounced difference in gene expression profiles suggests that *SIGLEC7*⁺ dNK cells may represent a distinct immunological subset that contributes to aberrant inflammatory signaling in RPL. Given the known inhibitory role of Siglec-7, its expression may reflect a compensatory, but insufficient, regulatory attempt within an otherwise dysregulated immune microenvironment. These findings implicate *SIGLEC7*⁺ dNK cells as potential modulators of maternal immune tolerance breakdown at the maternal–fetal interface in RPL.

### Paired loss of EVT sialylation and gain of Siglec-7^+^ dNK cells in RPL reveals disrupted maternal–fetal immunological crosstalk

**Fig. 2:**
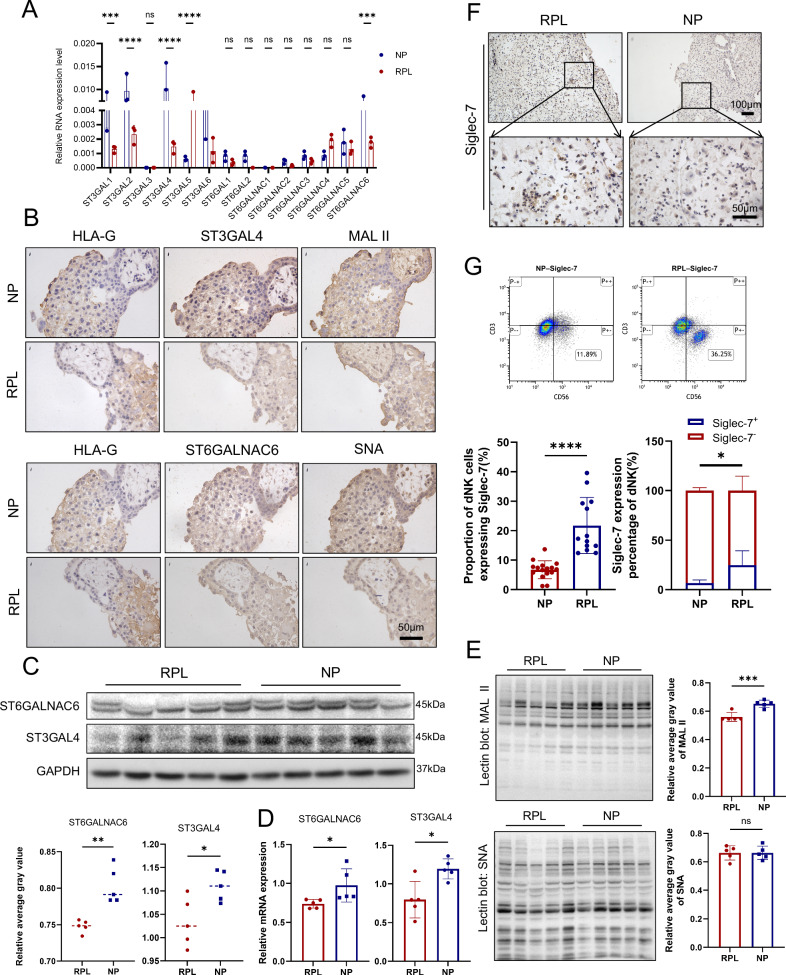
Validation of single‒cell transcriptomic findings in clinical samples. **A** RT‒qPCR results showing the differential expression of sialyltransferases in EVT cells from NP and RPL samples (*n* = *3 NP; n* = *3 RPL*). **B** Immunohistochemical staining of human placental villous tissues showing HLA-G⁺ EVT cell and the expression of ST3GAL4 and ST6GALNAC6, along with positive signals for MAL II (which detects α 2,3-linked sialic acid) and SNA (which detects α 2,6-linked sialic acid) in NP and RPL samples. **C** Western blot analyses of ST3GAL4 and ST6GALNAC6 protein levels in EVT cells isolated from NP and RPL tissues (*n* = 5 per group). **D** RT‒qPCR analyses of *ST3GAL4* and *ST6GALNAC6* mRNA levels in EVT cells (*n* = 5 per group). **E** Lectin blot analysis of α 2,3-linked sialylation and α 2,6-linked sialylation in EVT cell via MAL II and SNA in NP and RPL samples (*n* = 5 per group). **F** Immunohistochemical staining of human decidual tissues showing Siglec-7 expression in the NP and RPL groups. **G** Flow cytometric analysis of Siglec-7 expression in CD3^−^CD56⁺ dNK cells from NP and RPL decidual cells (*n* = *16 NP; n* = *13 RPL*). Comparison of the proportions of the Siglec7⁺ and Siglec7⁻ subsets among CD3⁻CD56⁺ dNK cells revealed a marked imbalance in the RPL samples. Statistical significance was determined via a two-tailed Student’s *t* test. *P < 0.05, **P < 0.01, ***P < 0.001, ****P < 0.0001, ns not significant.

To validate and extend our scRNA-seq findings of reduced sialyltransferase gene expression in EVT cells, we first assessed the expression of sialyltransferase family members in chorionic villus tissues from RPL and NP samples. Using real-time quantitative polymerase chain reaction (RT‒qPCR), we observed a general downregulation of multiple sialyltransferases in RPL villi compared with those in NP control villi. From this panel, we determined that *ST3GAL4* and *ST6GALNAC6* presented the most pronounced reduction in expression levels in RPL tissues (Fig. 2A). To confirm their cellular localization, immunohistochemical (IHC) staining of chorionic villi tissues revealed that both enzymes were predominantly expressed in EVT cells, and lectin staining via MAL II (which detects α 2,3-linked sialic acid) and SNA (which detects α 2,6-linked sialic acid) further demonstrated reduced sialylation levels in RPL villi tissues (Fig. 2B). Consistent with these findings, primary EVT cells isolated from RPL samples presented significantly lower mRNA levels (by RT‒qPCR) and protein levels (by western blot [WB]) of ST3GAL4 and ST6GALNAC6 than NP controls (Fig. 2C, D). Next, lectin blotting using MAL II demonstrated reduced sialylation levels in isolated EVT cells, which were consistent with the transcriptional and protein levels of ST3GAL4 (Fig. 2E).

To explore changes in maternal immune sensing of sialylation, we investigated Siglec-7 expression in decidual tissues from the same cohort. IHC staining of decidual tissues from RPL samples revealed strong Siglec-7 positivity (Fig. 2F), supporting the scRNA-seq findings of upregulated *SIGLEC7* expression in dNK cell clusters in RPL samples (Fig. 1F). Flow cytometric analysis of decidual lymphocytes from an expanded cohort (*n* = 13 RPL, *n* = 16 NP) further revealed that surface Siglec-7 expression was significantly higher, specifically on the CD3⁻CD56⁺ dNK cell subset, in RPL patients compared to NP controls (Fig. 2G). Moreover, the proportion of Siglec-7⁺ dNK cells was markedly greater in RPL samples than in NP samples, indicating an imbalance in the Siglec-7⁺ and Siglec-7⁻ dNK subsets in the RPL decidual environment (Fig. 2G). Collectively, these findings reinforce the concept of coordinated alterations in sialylation and Siglec-7–mediated immune interactions at the maternal–fetal interface in RPL.

### Glycoproteomic profiling identifies sialylated membrane proteins as potential Siglec-7 ligands

**Fig. 3:**
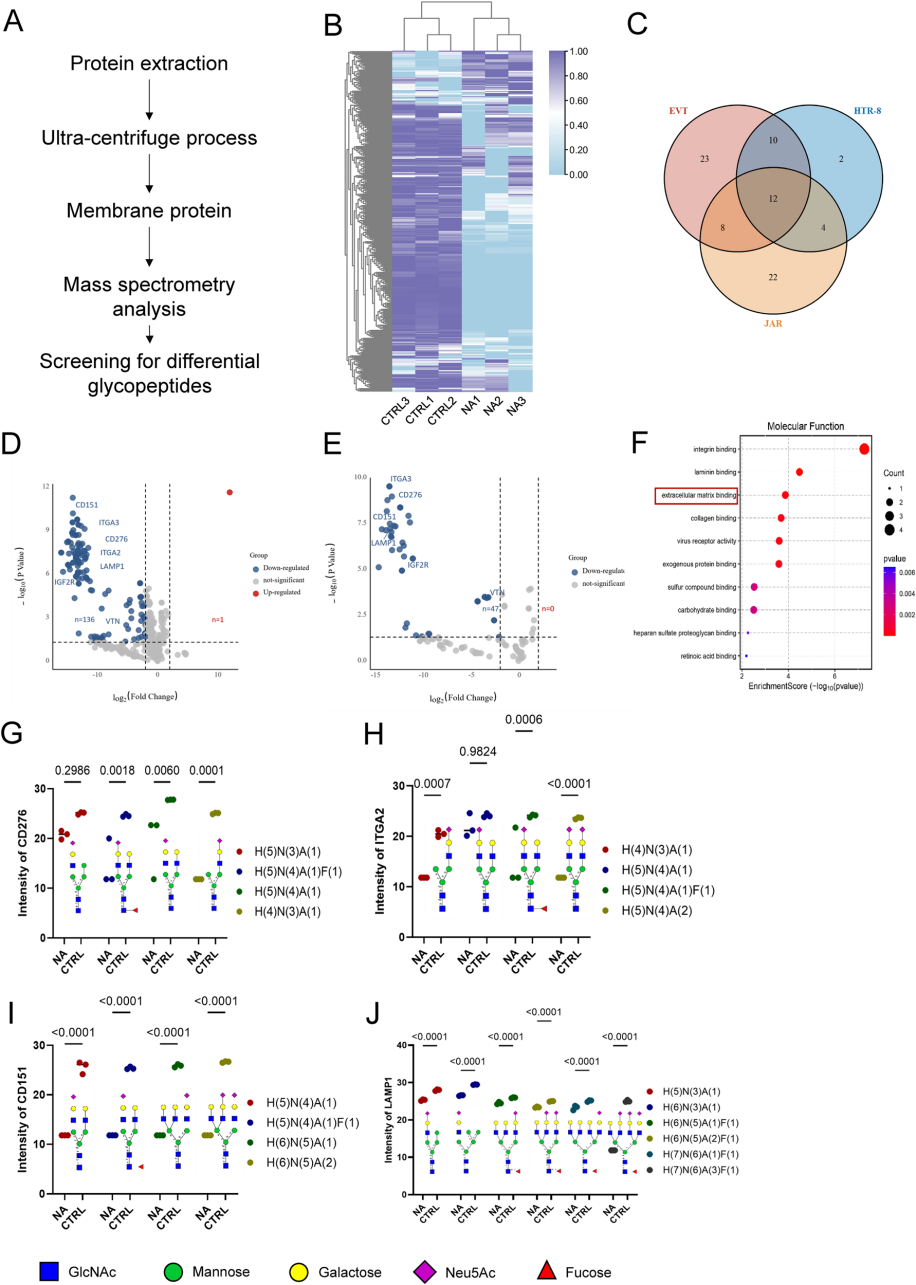
Identification and functional validation of sialylated membrane glycoproteins that interact with Siglec-7 in EVT cells. **A** Workflow depicting membrane protein extraction from primary EVT cells followed by glycopeptide enrichment and mass spectrometry analysis. **B** Heatmap showing altered glycopeptide profile after neuraminidase (NA) treatment. **C** Venn diagram illustrating glycoprotein overlap among EVT, JAR, and HTR-8 cells identified by glycoproteomics; greater overlap was observed between HTR-8 and EVT cells. Volcano plot identifying differentially expressed glycoproteins between the NA and CTRL groups in EVTs (**D**) and HTR-8 cells (**E**). Selected significantly altered glycoproteins are labeled. **F** Gene Ontology (GO) enrichment analysis of differentially expressed glycoproteins highlights associations with extracellular matrix binding functions. Relative abundance of terminally sialylated glycoforms on selected glycoproteins—CD276 (**G**), ITGA2 (**H**), CD151 (**I**), and LAMP1 (**J**)—reveals a significant reduction in sialylated species upon NA treatment. Data represent the mean ± SEM from *n* = *3* biological replicates. Two-way ANOVA was used to assess the main and interaction effects of glycan type and treatment. *P < 0.05 was considered statistically significant. H, N, F, and A represent hexose Hex, N-acetylhexosamine HexNAc, deoxyhexose dHex, and sialic acid NeuAc, respectively.

To investigate the potential glycoproteins in EVTs that might interact with Siglec-7 in maternal dNK cells, we performed glycoproteomic profiling. Given that Siglec-7 is a cell-surface receptor expressed on dNK cells, only sialylated glycoproteins present on the plasma membrane of EVT cells are accessible for direct interactions. Therefore, we focused our analysis on membrane glycoproteins extracted from primary EVT cells derived from NP samples under both control conditions and after the enzymatic removal of terminal sialic acids via neuraminidase (NA; Fig. 3A). Mass spectrometry–based glycoproteomic profiling, followed by relative quantification, revealed a marked reduction in the sialylation of membrane glycoproteins upon NA treatment (Fig. 3B). Differential expression analysis (|log₂ fold change|>2, *P* < 0.05) revealed a subset of membrane glycoproteins whose sialylation was most strongly reduced by NA treatment, as visualized via a volcano plot (Fig. 3D, E). Functional enrichment analysis of these differentially sialylated membrane proteins revealed that they were associated primarily with cell adhesion and extracellular matrix organization (Fig. 3F), processes essential for EVT invasion and the establishment of maternal–fetal immune tolerance.

Among the candidate glycoproteins identified through glycoproteomic screening, CD276, ITGA2, CD151, and LAMP1 were prioritized for further investigation. These proteins exhibited the most pronounced reduction in sialylation following NA treatment, which is consistent with the downregulation of the sialylation machinery observed in RPL-EVT cells. Importantly, these membrane glycoproteins play critical roles in cell migration and invasion [10–13] and are highly relevant to EVT function at the maternal–fetal interface. Therefore, we focused on subsequent validation experiments using these four proteins. Detailed glycan profiling further confirmed a marked reduction in the terminal sialic acid residues on multiple glycan chains linked to these proteins (Fig. 3G–J). Building on these findings, we next examined whether the reduced sialylation of these membrane proteins compromises their interaction with Siglec-7 using lectin pull-down and co-immunoprecipitation assays.

### ST6GALNAC6–mediated sialylation enhances the interaction of CD151, CD276, ITGA2, and LAMP1 with Siglec-7 on dNK cells

**Fig. 4:**
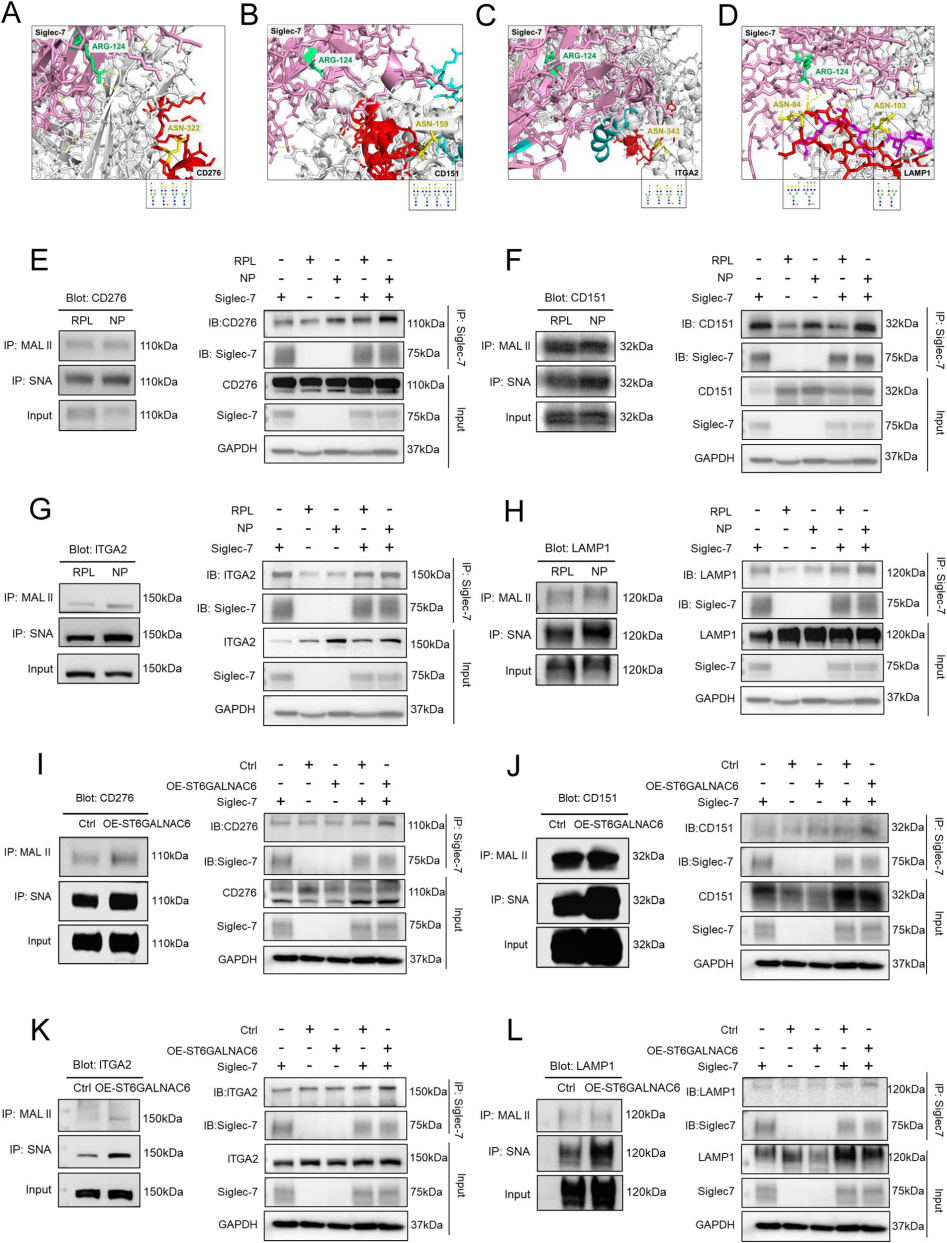
Glycosylation of membrane glycoproteins with sialic acid and their interaction with Siglec-7. Structural modeling using AlphaFold3 predicts potential recognition interfaces between CD276 (**A**), CD151 (**B**), ITGA2 (**C**), LAMP1 (**D**) with Siglec-7. Lectin pull-down assays demonstrated that both α 2,6- and α 2,3-linked sialylation of CD276 (**E**), CD151 (**F**), ITGA2 (**G**), and LAMP1 (**H**) were decreased in EVT cells derived from RPL compared with those from NP. Co-immunoprecipitation analysis further revealed that the interaction between these glycoproteins and Siglec-7 was correspondingly reduced in RPL-EVT cells. To validate this regulatory relationship, we overexpressed ST6GALNAC6 in HEK-293FT cells. Overexpression of ST6GALNAC6 increased α 2,6- and α 2,3-linked sialylation of CD276 (**I**), CD151 (**J**), ITGA2 (**K**), and LAMP1 (**L**). Co-immunoprecipitation analysis confirmed that ST6GALNAC6 enhances the sialylation of multiple membrane glycoproteins and thereby promotes their interaction with Siglec-7.

As shown in Fig. 2, the expression of ST3GAL4 and ST6GALNAC6 was positively correlated with the overall sialylation levels of total EVT proteins, suggesting their potential role in regulating surface sialylation. To explore the structural basis underlying the potential interactions between these glycoproteins and Siglec-7, we used AlphaFold3-based structural modeling. The predicted structures revealed clusters of surface-exposed residues on CD276 (ASN-322), CD151 (ASN-159), ITGA2 (ASN-343), and LAMP1 (ASN-84, ASN-103), which may serve as potential sialylation sites and putative Siglec-7 recognition interfaces (Fig. 4A–D). Primary EVT samples from RPL patients showed significant downregulation of ST3GAL4 and ST6GALNAC6 (Fig. 2C, D). Lectin pull-down assays further revealed reduced sialylation of CD276, CD151, ITGA2, and LAMP1 in RPL-EVT cells. Binding of these glycoproteins to Siglec-7 was markedly decreased in RPL-EVTs relative to NP, suggesting that impaired sialylation weakens Siglec-7 engagement and may disrupt immune tolerance at the maternal–fetal interface (Fig. 4E–H).

To further investigate this relationship, we overexpressed *ST3GAL4* (*ST3GAL4* OE cells) and *ST6GALNAC6* (*ST6GALNAC6* OE cells) in HEK-293FT cells (Supplementary Fig. 4A, B). The overexpression of *ST6GALNAC6* in HEK-293FT cells led to a marked increase in α 2,6-linked sialylation of these proteins, whereas α 2,3-linked sialylation also moderately increased (Fig. 4I–L). In contrast, the overexpression of *ST3GAL4* did not significantly affect the α 2,3-linked sialylation of these proteins, and only CD151 resulted in a modest increase in α 2,6-linked sialylation (Supplementary Fig. 4D–G). Total protein lysates from *SIGLEC7* OE cells and *ST6GALNAC6* OE cells were mixed to allow for potential sialic acid–Siglec-7 interactions in vitro. Co-immunoprecipitation was performed via the use of anti-Siglec-7 antibodies to pull down Siglec-7 and its interacting partners. Immunoblotting with antibodies specific for CD276, CD151, ITGA2, and LAMP1 revealed that the interactions of these proteins with Siglec-7 were significantly greater in the presence of ST6GALNAC6 overexpression than in the control conditions (Fig. 4I–L). Collectively, these results demonstrate that ST6GALNAC6 promotes the sialylation of CD276, CD151, ITGA2, and LAMP1, thereby increasing their potential to engage Siglec-7.

### The impairment of sialic acid–Siglec-7 interactions inhibits EVT cell migration and invasion in coculture assays

**Fig. 5:**
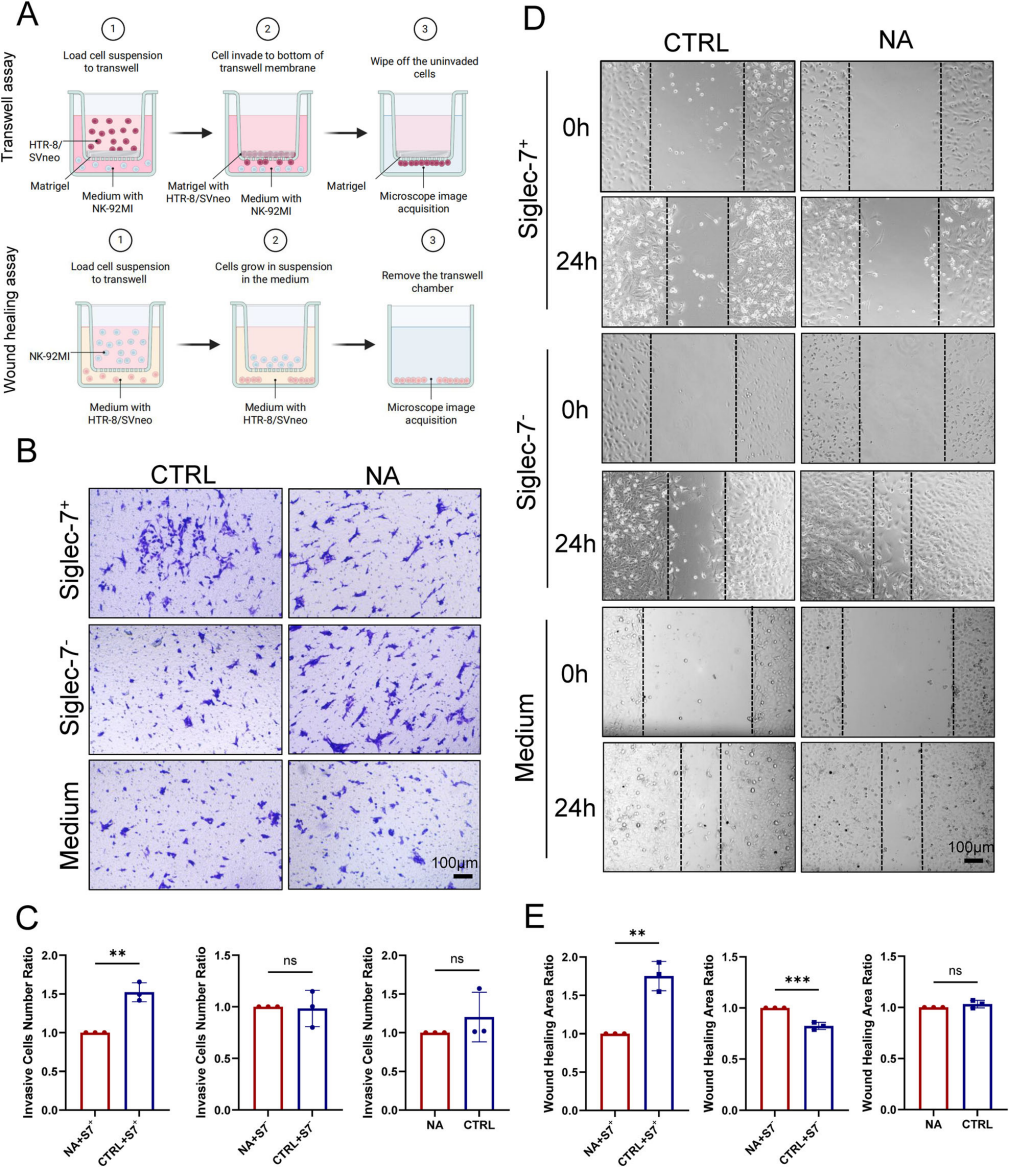
Sialic acid–Siglec-7 interactions regulate HTR-8 cell migration and invasion via NK-92MI cell coculture assays. **A** Schematic overview of the Transwell-based coculture assay for evaluating HTR-8 cell invasion (top) and migration (bottom) in the presence of NK-92MI cells. Figure created with BioRender.com (https://BioRender.com/psweqqc). **B** For the invasion assay, 5 × 10⁴ HTR-8 cells (treated with or without NA) were seeded in the upper chamber of a Transwell system. The lower chamber contained either 1.5 × 10⁵ Siglec-7⁺ or Siglec-7⁻ NK-92MI cells or medium alone. After 24 h, the invading cells were stained with crystal violet. **C** Quantification of invading HTR-8 cells via ImageJ revealed a significant reduction in invasion in NA-treated cells cocultured with Siglec-7⁺ NK-92MI cells. **D** For the migration assay, 2.5 × 10⁴ HTR-8 cells were seeded in the lower chamber and allowed to adhere for 24 hours. After NA treatment, 7.5 × 10⁴ Siglec-7^+/^⁻ NK-92MI cells (or medium) were added to the upper chamber. The scratch area of the adherent HTR-8 cells was imaged at 0 h and 24 h. **E** ImageJ-based quantification of wound closure revealed significantly impaired migration in NA-treated HTR-8 cells cocultured with Siglec-7⁺ NK-92MI cells. Statistical significance was determined by Student’s two-tailed *t* test; **P < 0.01, ***P < 0.001; ns not significant.

To further assess the functional significance of sialic acid–Siglec-7 interactions in EVT behavior, we established a Transwell-based coculture system comprising HTR-8/SVneo (HTR-8) cells and Siglec-7⁺ or Siglec-7⁻ NK-92MI cells (Fig. 5A). HTR-8 cells were pretreated with NA to enzymatically remove surface sialic acids, and the effect of sialic acid–Siglec-7 binding was evaluated. In the invasion assay, we observed no significant difference in the invasive capacity of HTR-8 cells with or without NA treatment. However, NA-pretreated HTR-8 cells exhibited a marked reduction in invasive ability when cocultured with Siglec-7⁺ NK-92MI cells compared with the untreated controls (Fig. 5B, C). To evaluate cell migration, we performed wound-healing assays under similar coculture conditions. NA treatment alone did not significantly affect the migratory ability of HTR-8 cells. In contrast, NA-pretreated cells displayed a pronounced reduction in migration when cocultured with Siglec-7^+^ NK-92MI cells (Fig. 5D, E). To ensure that NA treatment did not alter the baseline physiological state or proliferation of HTR-8 cells, we performed DAPI staining to assess the cell numbers in the NA-treated and untreated groups. No significant differences in cell numbers were observed, confirming that the observed reductions in invasion and migration were not due to impaired proliferation (Supplementary Fig. 5A).

### Sialic acid–Siglec-7 interaction is correlated with IL-8–STAT3 signaling in EVT–dNK coculture

**Fig. 6:**
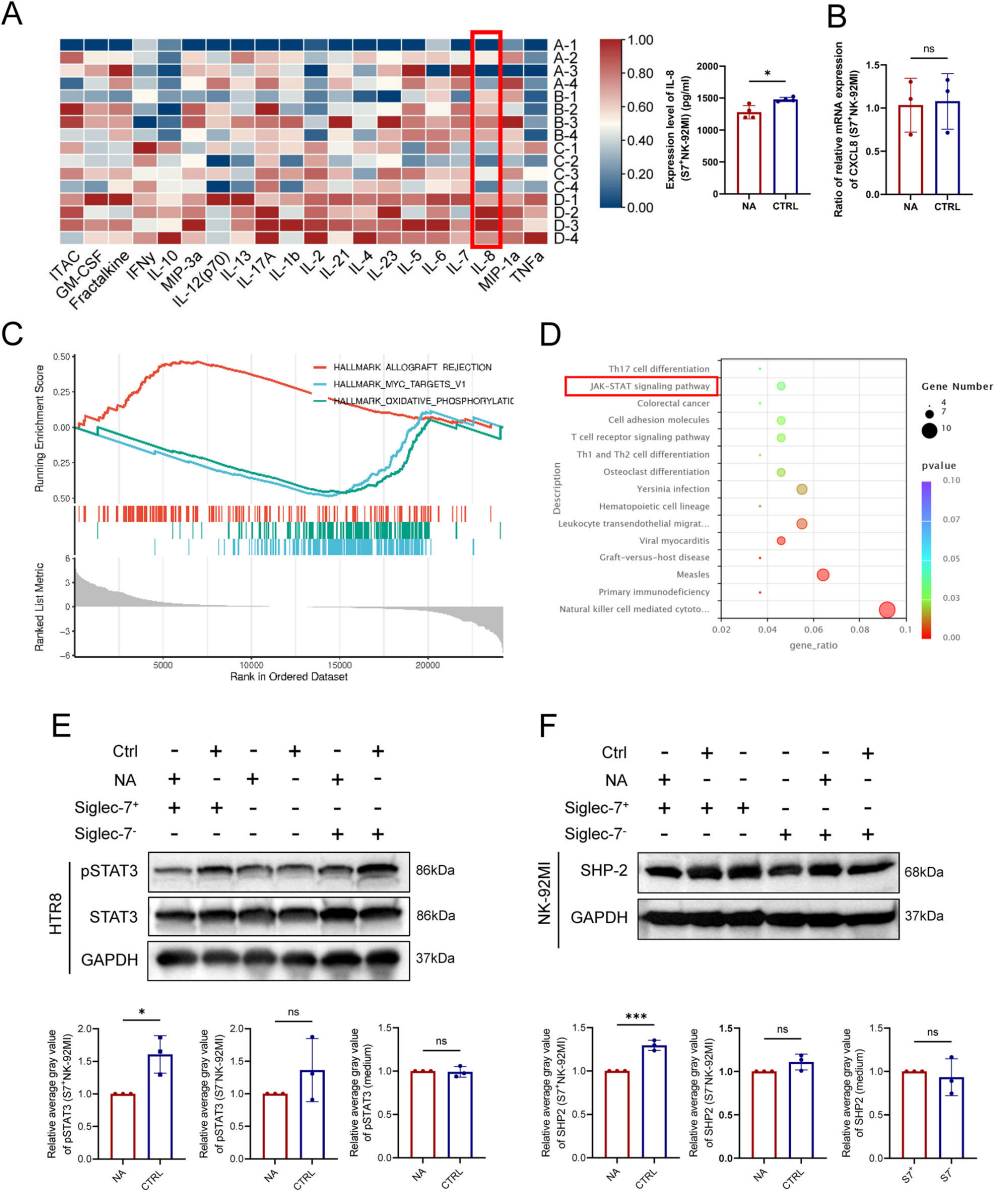
Sialic acid–Siglec-7 interactions regulate IL-8–STAT3 signaling in HTR-8 and NK-92MI cell cocultures. **A** Cytokine levels in the culture supernatants from cocultures of HTR-8 cells and NK-92MI cells were measured via a multiplex liquid-phase cytokine assay following the manufacturer’s instructions. Among all the tested cytokines, IL-8 was significantly different between the groups (*P < 0.05). Group A: NA-treated HTR-8 cells cocultured with Siglec-7^+^ NK-92MI cells; Group B: untreated HTR-8 cells with Siglec-7^+^ NK-92MI cells; Group C: NA-treated HTR-8 cells with Siglec-7⁻ NK-92MI cells; Group D: untreated HTR-8 cells with Siglec-7⁻ NK-92MI cells. **B** *CXCL8* mRNA levels in NK-92MI cells were not significantly altered between NA and CTRL groups. NA: NA-treated HTR-8 cells cocultured with Siglec-7^+^ NK-92MI cells; CTRL: untreated HTR-8 cells with Siglec-7^+^ NK-92MI cell. **C** Transcriptomic analysis of the same experimental groups revealed that NA treatment significantly upregulated immune activation pathways (e.g., allograft rejection) while downregulating MYC target genes and oxidative phosphorylation, both of which are associated with cellular metabolism and proliferation, as shown by GSEA. **D** KEGG pathway enrichment analysis based on RNA-seq data revealed significant enrichment of the JAK–STAT3 pathway, a well-known regulator of cell migration and invasion. **E** Western blot analysis revealed that STAT3 phosphorylation was markedly decreased in NA-treated HTR-8 cells cocultured with Siglec-7⁺ NK-92MI cells. **F** SHP-2 expression was significantly decreased in cocultures of NA-treated HTR-8 cells with Siglec-7⁺ NK-92MI cells. Statistical significance was determined by Student’s two-tailed *t* test; *P < 0.05, ***P < 0.001; ns not significant.

To further investigate the molecular mechanism underlying the sialic acid–Siglec-7 interaction in EVT–dNK cell communication, we established a coculture system comprising HTR-8 cells and NK-92MI cells. Indeed, high-sensitivity Luminex assays confirmed that IL-8 levels in the culture supernatant were significantly lower when NA-treated HTR-8 cells were cocultured with Siglec-7⁺ NK-92MI cells than in untreated controls and Siglec-7^+^ NK-92MI cocultures (Fig. 6A). Notably, RT‒qPCR revealed no significant differences in *CXCL8* mRNA levels in Siglec-7⁺ NK-92MI cells between groups (Fig. 6B), suggesting that the regulation of IL-8 secretion likely occurs at the posttranscriptional level in NK cells rather than at the transcriptional level.

To further explore the downstream effects of altered IL-8 secretion in EVT cells, we performed transcriptomic profiling of HTR-8 cells under various coculture conditions. Gene set enrichment analysis (GSEA) revealed that NA-treated HTR-8 cells cocultured with Siglec-7⁺ NK-92MI cells exhibited significant activation of immune-related pathways, including the “Allograft Rejection” hallmark, while concurrently downregulating MYC target genes and oxidative phosphorylation pathways, both of which are associated with cellular metabolism and proliferation (Fig. 6C). Additionally, Kyoto Encyclopedia of Genes and Genomes (KEGG) pathway enrichment analysis revealed significant enrichment of the JAK–STAT3 pathway in these cells (Fig. 6D). To validate these transcriptomic findings functionally, we assessed the phosphorylation of STAT3 in HTR-8 cells. WB analysis revealed that the level of phosphorylated STAT3 was significantly lower in NA-treated HTR-8 cells cocultured with Siglec-7⁺ NK-92MI cells than in untreated control and Siglec-7^+^ NK-92MI cocultures (Fig. 6E). Within this coculture system, WB analysis revealed that SHP-2 protein expression was significantly lower in Siglec-7⁺ NK-92MI cells when cocultured with NA-treated HTR-8 cells than in untreated controls. Under the same conditions, however, no significant change in SHP-2 expression was observed in Siglec-7⁻ NK-92MI cells (Fig. 6F). Given that SHP-2 is known to positively regulate IL-8 secretion in immune cells [14–16], this reduction in SHP-2 may account for the decreased IL-8 production observed under NA-treated coculture conditions. Collectively, these results suggest that the loss of sialylation in HTR-8 cells disrupts sialic acid–Siglec-7 interactions, leading to reduced SHP-2 protein expression and, consequently, may decrease IL-8 secretion in dNK cells. Decreased IL-8 availability may subsequently compromise STAT3 phosphorylation in EVT cells, ultimately affecting their invasive and migratory capacities.

### Sialic acid–Siglec-7 interaction underlies a critical ligand‒receptor recognition module that mediates IL-8–STAT3 signaling between EVT‒dNK cells

**Fig. 7:**
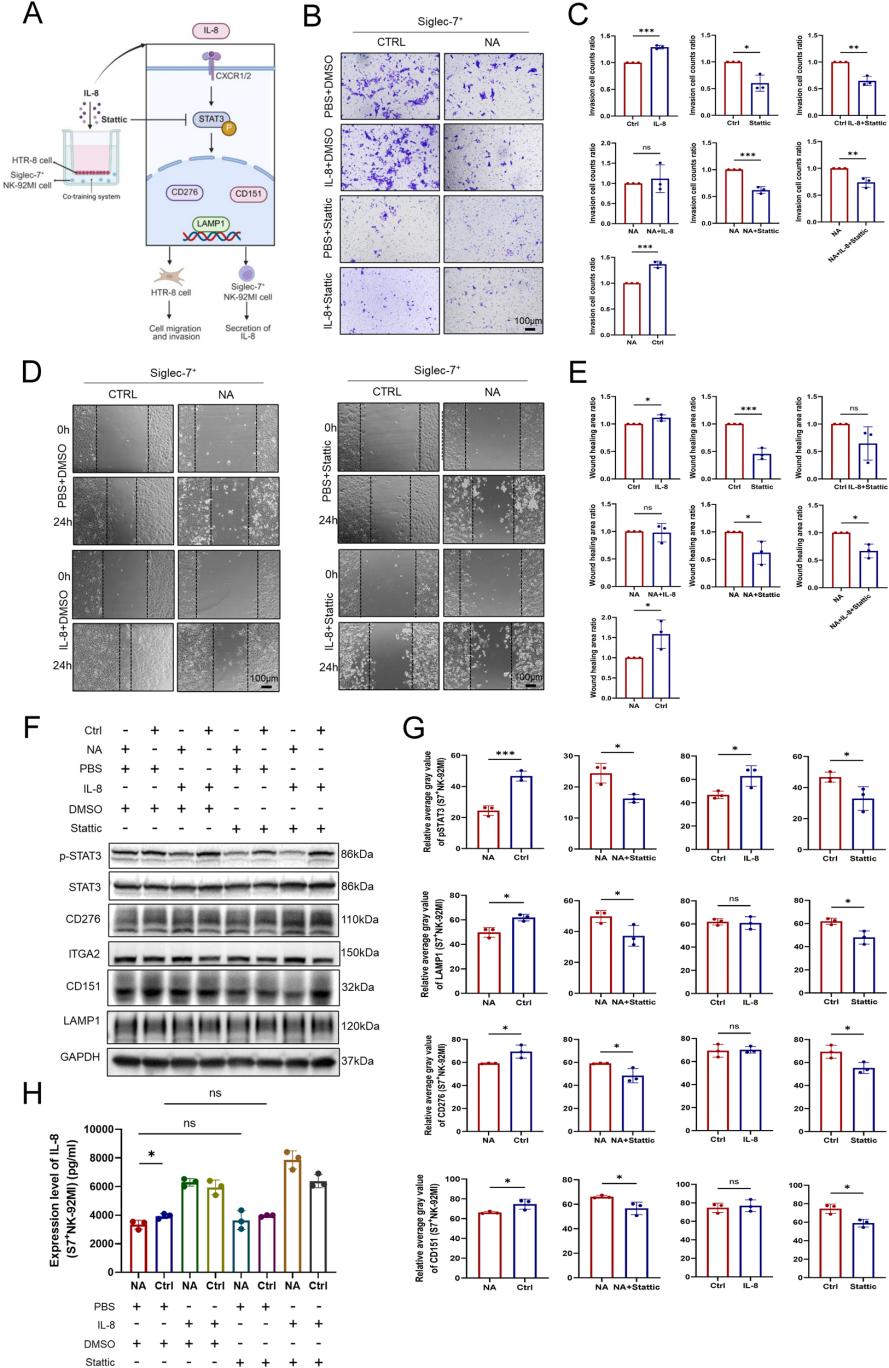
Modulation of the IL-8–STAT3 pathway affects HTR-8 cell invasion and migration. **A** Schematic diagram illustrating the signaling pathways involved in the regulation of IL-8 production and STAT3 phosphorylation. Figure created with BioRender.com (https://BioRender.com/0ig17np). **B**, **C** For Transwell invasion assays, HTR-8 cells pretreated with NA were seeded in the upper chambers of Transwell inserts. Siglec-7⁺ NK-92MI cells were added to the lower chamber. After 24 h, invading HTR-8 cells on the lower membrane surface were stained with crystal violet. Four treatment conditions were applied: PBS + DMSO (control), IL-8 + DMSO, PBS+Stattic (STAT3 inhibitor), and IL-8+Stattic. IL-8 treatment enhanced HTR-8 cell invasion, and Stattic treatment suppressed cell invasion. The combination of IL-8 and Stattic did not rescue the reduced invasive capacity. **D**–**E** For migration assays, HTR-8 cells were seeded in the lower chamber and pretreated with NA for 24 h. Siglec-7⁺ NK-92MI cells were then seeded in the upper chamber. Immediately after cell addition, the width (0 h) of the HTR-8 monolayer in the lower chamber was measured. Scratch closure was monitored after 24 h under the same four treatment conditions as in (**B**). IL-8 treatment promoted wound healing, and Stattic treatment suppressed cell migration. The combination of IL-8 and Stattic did not rescue the reduced migratory capacity. **F**–**G** Following the same four treatments, protein lysates from HTR-8 cells co-cultured with Siglec-7^+^ NK-92MI cells were analyzed by western blot. IL-8 treatment significantly increased the phosphorylation of STAT3. Stattic treatment reduced STAT3 phosphorylation and suppressed the expression of CD276, CD151, or LAMP1. Statistical significance was determined by Student’s two-tailed *t* test; *P < 0.05, **P < 0.01, ***P < 0.001, ns not significant. **H** IL-8 levels in culture supernatants were measured via ELISA. No significant differences were observed between the Statistic-treated and control groups. The data were analyzed via one-way ANOVA; *P < 0.05, ns not significant.

The interaction between EVT and dNK cells at the maternal–fetal interface is essential for the maintenance of pregnancy and involves multiple well-characterized receptor–ligand pairs that promote EVT invasion while tempering dNK cell immune responses [17]. Thus, we investigated the potential involvement of the IL-8–STAT3 axis in the sialic acid–Siglec-7 interaction identified in our earlier study. We employed a Transwell coculture system comprising HTR-8 cells (with or without NA treatment) and Siglec-7^+/−^ NK-92MI cells supplemented with recombinant IL-8 protein or Stattic, a specific inhibitor of STAT3 phosphorylation at Tyr705 (Fig. 7A). In cocultures of untreated HTR-8 and Siglec-7^+^ NK-92MI cells, IL-8 supplementation markedly enhanced cell migration and invasion, whereas Stattic treatment significantly suppressed these functions. Interestingly, although Stattic also inhibited the migration and invasion of NA-treated HTR-8 cells cocultured with Siglec-7^+^ NK-92MI cells, IL-8 supplementation failed to reverse these functional impairments (Fig. 7B–E).

Consistent with these phenotypic observations, WB analysis revealed that IL-8 treatment increased STAT3 phosphorylation in untreated HTR-8 cells cocultured with Siglec-7^+^ NK-92MI cells. However, IL-8 did not appreciably increase STAT3 phosphorylation or CD276, CD151, or LAMP1 protein levels in NA-treated HTR-8 cells. Furthermore, Stattic treatment decreased STAT3 phosphorylation and the expression of CD276, LAMP1, and CD151 (Fig. 7F, G). Given that NA treatment diminishes the surface sialylation of HTR-8 cells (Supplementary Fig. 3C), thereby disrupting sialic acid-Siglec-7 interactions, the inability of exogenous IL-8 to restore cell invasiveness highlights the functional importance of this receptor–ligand interaction in EVT cell behavior.

Taken together, these findings support the notion that the sialic acid-Siglec-7 interaction is linked to the IL-8–STAT3 signaling pathway in EVT cells and that this axis plays a key role in EVT migration and invasion. Previous studies have proposed a bidirectional feedback loop between IL-8 and STAT3 [18, 19]. To assess whether such feedback occurred in our system, we quantified the IL-8 levels in the coculture supernatants via ELISA. No significant differences were detected between the STAT3–treated and control groups, indicating that, in this context, IL-8 likely acts upstream to positively regulate STAT3 phosphorylation rather than being regulated by it (Fig. 6H).

### ST6GALNAC6 supplementation improves the impaired invasiveness of RPL–derived EVT cells

**Fig. 8:**
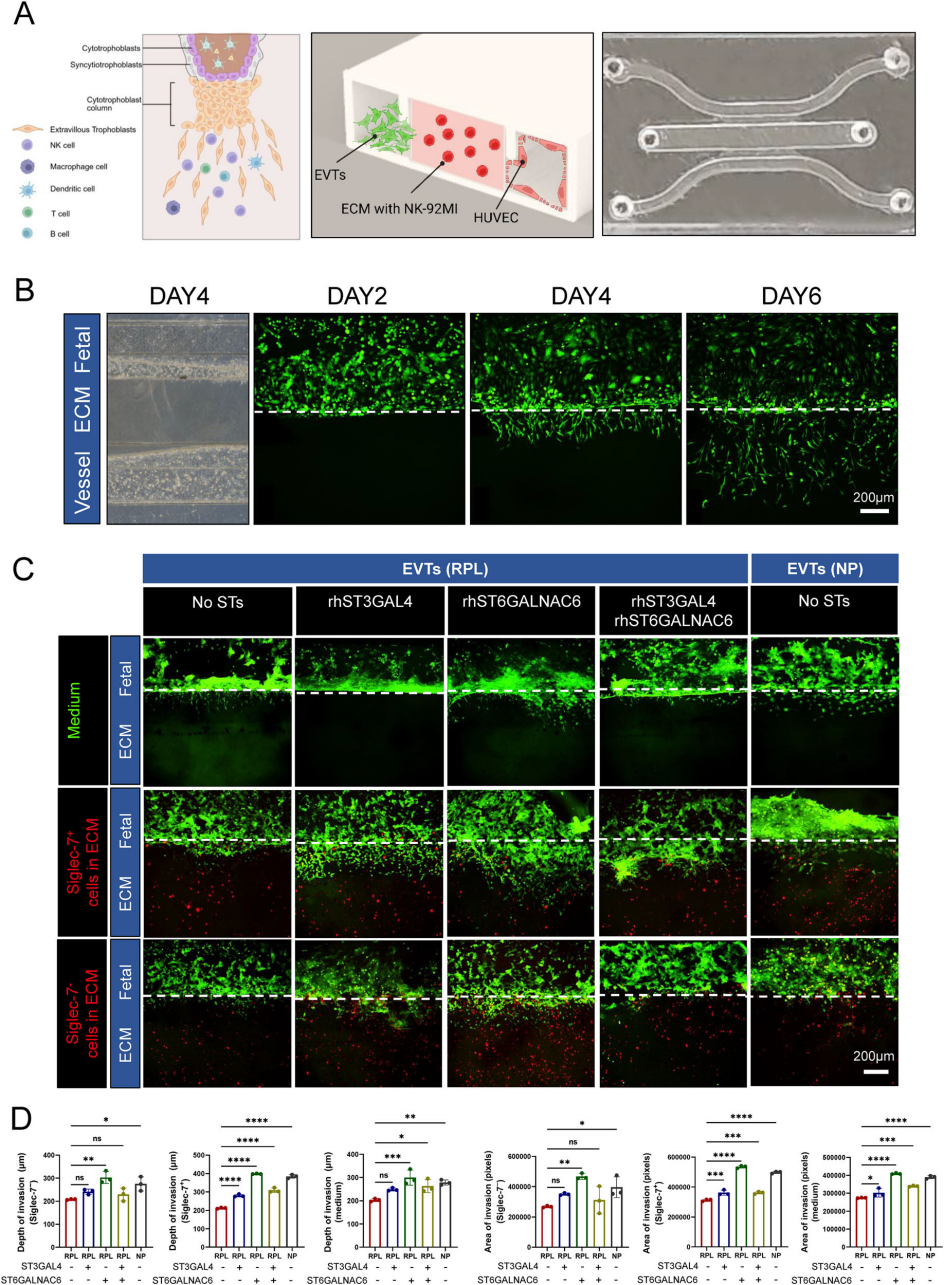
ST6GALNAC6 rescues impaired invasiveness of RPL-derived EVT cells in a microfluidic implantation-on-chip model. **A** Microfluidic implantation-on-a-chip model was established by seeding EVT cells into the fetal chamber and HUVEC cells into the maternal chamber. A layer of low-growth-factor matrigel containing NK-92MI cells was introduced into the central chamber to mimic the decidual–trophoblast interface. Figure created with BioRender.com (https://BioRender.com/x60p1q7). **B** EVT cells displayed increased invasion depth and area over time within the chip-based implantation model. **C**, **D** Recombinant ST3GAL4 and ST6GALNAC6 were added individually or in combination to RPL-derived EVT cells. The matrigel was supplemented with either Siglec-7⁺ or Siglec-7⁻ NK-92MI cells. Images were acquired after 24 h using an Olympus confocal microscope. Quantification revealed that ST6GALNAC6, alone or in combination with ST3GAL4, significantly enhanced the invasion depth and area of RPL EVT cells. ST3GAL4 alone had a minimal effect. All glycosyltransferase treatments improved invasiveness in the presence of Siglec-7⁺ NK cells. The data are presented as the means ± SDs. Statistical analysis was performed via one-way ANOVA. *P < 0.05, **P < 0.01, ***P < 0.001, ns not significant.

Given that exogenous IL-8 failed to restore STAT3 activation and EVT invasiveness in NA-treated cocultures (Fig. 7), we tested whether restoring surface sialylation with ST6GALNAC6 could enhance sialic acid-Siglec-7 interactions and thereby rescue EVT invasion. To further validate this mechanism under physiologically relevant conditions, we established a microfluidic implantation–on–a–chip model consisting of three parallel channels representing the fetal compartment, extracellular matrix (ECM) scaffold, and maternal compartment [20] (Fig. 8A). EVT cells seeded in the fetal channel accumulated at the hydrogel interface and progressively invaded the ECM scaffold over time, allowing quantitative assessment of the invasion depth and area on days 2, 4, and 6 postseeding (Fig. 8B).

Using this model, we first compared the invasive capacity of EVT cells derived from NP and RPL samples in the presence or absence of Siglec-7⁺ or Siglec-7⁻ NK-92MI cells embedded within the ECM. Compared with NP-derived EVT cells, RPL-derived EVT cells exhibited significantly reduced invasion depth and area (Supplementary Fig. 9B, C). Notably, coculture with Siglec-7⁺ NK-92MI cells enhanced EVT invasion compared with coculture with Siglec-7⁻ NK cells, suggesting that sialic acid–Siglec-7 interactions may promote EVT invasiveness (Supplementary Fig. 9B, C).

To assess whether restoring sialylation could rescue EVT invasion, RPL-derived EVT cells were treated with recombinant ST3GAL4, ST6GALNAC6, or both for 24 h. Compared with no treatment, treatment with ST3GAL4 alone had a minimal effect on EVT invasion, whereas treatment with ST6GALNAC6 significantly improved EVT invasiveness (Fig. 8C, upper panel; Fig. 8D). Importantly, in the presence of Siglec-7⁺ NK cells embedded in the ECM, all treatments—ST3GAL4, ST6GALNAC6, or their combination—markedly increased EVT invasion depth and area (Fig. 8C, middle panel; Fig. 8D). In contrast, coculture with Siglec-7⁻ NK-92MI cells did not significantly affect EVT invasiveness under any treatment condition (Fig. 8C, lower panel; Fig. 8D). Collectively, these results indicate that supplementation with sialyltransferases, particularly ST6GALNAC6, enhances EVT invasiveness in RPL by restoring surface sialylation, thereby facilitating sialic acid–Siglec-7 interactions and downstream signaling at the maternal–fetal interface.

## Discussion

RPL remains a multifactorial disorder, with insufficient trophoblast invasion during early pregnancy recognized as a critical contributor [21]. By integrating bulk and single–cell transcriptomics, glycoproteomics, microfluidic implantation-on-a-chip assays, and mechanistic perturbations, we identified the loss of terminal sialylation on EVT membranes as a molecular trigger that disabled the previously unrecognized ST6GALNAC6–sialic acid–Siglec-7–IL-8–STAT3 axis. De-sialylation interrupts glycan–dependent recognition by dNK cells, reduces IL-8 release, reduces STAT3 phosphorylation in EVTs, and compromises cell invasion. Restoration of α 2,6-linked sialic acid with recombinant ST6GALNAC6 rescues these defects in RPL-derived EVT cell invasion in organ–chip cultures, suggesting precision glycoediting may serve as a tractable therapeutic strategy for RPL.

Classical ligand–receptor pairs have long been central to models of maternal immune tolerance—yet they do not fully explain how EVT chemokine licensing couples with innate glycan sensing. These classical pathways include: dNK cell KIR recognition of EVT HLA-C, HLA-G dimer binding to LILRB1 for tolerance, and CD94-NKG2A/C interactions with HLA-E to balance NK cell activity [22]. Our data bridges this gap. In chorionic villi from RPL patients, we detected the coordinated downregulation of seven sialyltransferase genes, among which *ST3GAL4* and *ST6GALNAC6* were the most prominent. Meanwhile, the matched decidua displayed a selective expansion of proinflammatory Siglec-7⁺ dNK3 cells, along with a marked increase in *SIGLEC7* mRNA. Notably, we observed that bulk RNA-seq and scRNA-seq analyses yielded partially discordant results for sialyltransferase and sialidase expression in EVT populations, which likely arose from bulk data capturing signals from contaminating cell types (e.g., fibroblasts and macrophages; Supplementary Fig. 2C-G) in bulk tissue samples. Prior studies have shown that trophoblasts are enriched in sialoglycans [23], with key enzymes such as ST6GAL1 and ST8Sia catalyzing α 2,6-linked and α 2,8-linked sialylation, respectively, thereby supporting embryo implantation, EVT migration, and maternal immune tolerance [24]. For example, ST6GAL1-mediated α 2,6-linked sialylation of E-cadherin facilitates blastocyst adhesion, whereas polysialylation on EVT surfaces enhances ECM invasion [25]. Additionally, polysialic acid (α 2,8 linkages) on EVTs enhances trophoblast invasiveness by promoting migration through the ECM. This enhanced invasiveness can be reversed by the removal of polysialic acid [26, 27]. These studies underscore the critical role of sialylation in maintaining maternal–fetal tolerance and trophoblast function. However, these studies typically focused on individual enzymes or animal models and emphasized the role of sialylation in regulating trophoblast function rather than integrating EVT surface sialylation into the broader context of the decidual immune environment. By combining bulk and single–cell transcriptomics with functional assays, our study systematically revealed the coordinated downregulation of multiple sialyltransferases in RPL, uniquely linking this deficit to impaired Siglec-7 engagement and downstream disruption of IL-8–STAT3 signaling. Importantly, these changes occur independently of the HLA-C allotype or KIR genotype, positioning terminal sialylation as an orthogonal regulatory layer in placental development.

Mechanistically, Siglec-7 is an inhibitory receptor that is expressed on the surface of NK cells. It plays a crucial role in immune evasion in many high-incidence tumors, such as lung, breast, and pancreatic cancer [28–30]. Tumor cells often exhibit abnormally high levels of glycosylation, particularly of terminal sialic acid, which binds to Siglec-7 on NK cells and triggers its immunoreceptor tyrosine-based inhibition motif (ITIM) signaling domain, suppressing NK cell activation and cytotoxicity [31]. Notably, high expression of Siglec-7 or its ligands is correlated with poor prognosis in cancer patients [31]. Interestingly, a similar immunoregulatory mechanism is observed during pregnancy. We demonstrate that the dNK cells express Siglec-7. Furthermore, our findings reveal that EVT surfaces are enriched with sialylated glycans capable of binding to Siglec-7 receptor. This ligand-receptor pairing likely transmits inhibitory signals, thereby promoting immune tolerance at the maternal–fetal interface. However, other studies have shown that under specific conditions, Siglec-7 signaling can induce the production of cytokines such as IL-8 and TNF-α, suggesting that its effects are highly dependent on cell type and microenvironment [32]. In our experimental system, IL-8 appears to act more as a chemokine regulating EVT migration and invasion, rather than a typical proinflammatory mediator [8, 33]. Therefore, the Sialic acid–Siglec-7 interaction primarily exerts an immunosuppressive function through SHP-2, and the observed IL-8 secretion reflects a regulatory signal between dNK cells and EVTs rather than a classical inflammatory response. Prior work has shown that the complete loss of trophoblast sialylation in mice (e.g., via cytidine monophosphate N-acetylneuraminic acid synthetase knockout) triggers complement activation and neutrophil infiltration, ultimately leading to fetal loss [34]. However, the study did not examine how specific sialyltransferases on EVTs coordinate Siglec-7 signaling with downstream cytokine activation. Our work fills this gap by identifying ST6GALNAC6–mediated sialylation as an essential upstream switch that regulates IL-8–STAT3 signaling, thereby integrating glycan sensing with chemokine pathways to control EVT invasion.

Our study revealed that loss of sialylation reduces SHP-2 recruitment, leading to a marked decrease in IL-8 secretion from dNK cells and a subsequent reduction in p-STAT3 (Y705) in EVTs. Exogenous IL-8 rescued STAT3 activation and motility only when sialic acid-Siglec-7 contacts were intact, underscoring the nonredundancy of this glycoimmune checkpoint. De-sialylation inhibits the activation of STAT3, highlighting that the IL-8–STAT3 axis is a downstream effector of trophoblast motility. These data define sialic acid-Siglec-7 as an obligate upstream licensing mechanism that orchestrates EVT–dNK cross-talk. Importantly, by connecting sialylation to both immune regulation and trophoblast motility, our study highlights a dual role for sialylated glycans at the maternal–fetal interface, which has not been fully appreciated in prior work that often examined these aspects in isolation.

While emerging glycoproteomic studies have begun to characterize N‑glycan structures in placental tissues, including multi‑antennary and highly sialylated motifs typical of invasive trophoblasts, these analyses have often focused on bulk placental samples or secreted proteins such as pregnancy‑specific glycoprotein 1 (PSG1). Lectin-based enrichment and mass spectrometry have identified sialylated glycans and PSG isoforms that interact with maternal immune regulators. However, these approaches typically pool VCTs and EVTs and lack the resolution to define EVT–specific surface glycoproteomes [35]. Notably, no dedicated high-resolution glycoproteomic map of purified EVT subpopulations has been reported to date. This gap is significant since N‑glycosylation, particularly α 2,3-linked and α 2,6-linked sialylation, critically regulates EVT invasion, immune interactions, and ECM remodeling. Given the limited availability and heterogeneity of clinical RPL samples, we did not perform glycoproteomic profiling directly on RPL-derived EVTs. Instead, NA treatment of control EVTs was used to model the hyposialylated state observed in RPL, characterized by reduced levels of sialic acids. We observed that NA treatment significantly reduced EVT invasion in coculture with Siglec-7^+^ NK-92MI cells, recapitulating the clinical phenotype and enabling the identification of candidate membrane glycoproteins whose sialylation state may regulate EVT–dNK cross-talk. By applying this model, we overcame donor-specific variability while retaining a physiologically relevant experimental system. Furthermore, by combining glycoproteomic profiling with transcriptomic and functional assays, we systematically delineated EVT-specific surface glycoproteins and their altered sialylation under simulated RPL conditions.

Glycoproteomic profiling of NA-treated versus control EVT samples revealed CD151, CD276, ITGA2, and LAMP1 as the dominant Siglec-7 ligands, demonstrating, for the first time, that these four proteins carry terminal sialylation modifications relevant to EVT biology. Although previously implicated in tumor progression, these glycoproteins have not been systematically analyzed for sialylation in the context of EVT. CD151 (tetraspanin-24), for example, is a key regulator of redox homeostasis via ERK/Nrf2 signaling and is downregulated in preeclamptic placentas, although its glycosylation remains incompletely characterized [36]. CD276 (B7-H3) is stabilized on tumor cell surfaces by fucosylation; however, our data highlighted its sialylation-dependent binding to Siglec-7 in dNK cells [37]. ITGA2 (integrin α2), which is heavily N-glycosylated in cancer models with α 2,6 sialylation critical for ECM interactions [38], is also expressed in trophoblast progenitors [39], yet its glycosylation status in trophoblasts was not defined until our study. LAMP1, a well-known lysosomal glycoprotein with extensive N-glycosylation and polylactosamine extensions [29], has also emerged as a sialylated Siglec-7 ligand on EVT surfaces, potentially facilitating immune interactions during invasion. Notably, unlike canonical migratory regulators, such as MMP2 and MMP9, or the upstream sialyltransferase ST6GALNAC6 itself, which remained significantly unchanged (Supplementary Fig. 8A), our data demonstrate that sialic acid–Siglec-7–IL-8–STAT3 signaling selectively modulates the expression of CD151, CD276, ITGA2, and LAMP1. This finding suggests a nonredundant multivalent recognition platform that controls EVT invasion. NA treatment of EVT led to the loss of sialic acids in these proteins, abolishing Siglec-7 binding and profoundly reducing EVT invasion capacity, akin to direct Siglec-7 blockade. Functionally, these glycoproteins localize at the leading edge of the EVT and, when the combination is disrupted, recapitulate the invasive deficits observed with Siglec-7 pathway disruption. STAT3 inhibition downregulates CD151, CD276, and LAMP1, reinforcing a feed-forward loop wherein disrupted sialic acid–Siglec-7 signaling exacerbates EVT dysfunction.

We chose a human placenta–on–a–chip system rather than an animal model because of several key advantages [20]. First, Siglec‑7 and ST6GALNAC6‑mediated sialylation patterns exhibit marked species specificity, with human Siglec‑7 lacking direct murine counterparts and binding preferences not fully recapitulated in rodents [40, 41]. Second, the chip precisely controls human cell composition and the timing of dNK–EVT interactions, offering a more physiologically relevant model of the maternal–fetal interface. Finally, it avoids the ethical hurdles associated with generating EVT-specific manipulations in vivo while enabling higher-throughput mechanistic and pharmacologic testing. These findings warrant further validation in appropriate in vivo models or clinical specimens in the future.

In summary, our findings suggest that terminal sialylation is a key regulator of chemokine licensing at the maternal–fetal interface, integrating glycan recognition with cytokine signaling to control EVT invasion. These findings highlight the potential for patient stratification according to EVT sialylation status or Siglec-7⁺ dNK abundance, paving the way for targeted therapeutic strategies in RPL. Ultimately, targeted modulation of the ST6GALNAC6–sialic acid–Siglec-7–IL-8–STAT3 axis may offer a promising new avenue for treating RPL. Future studies using in vivo models and larger clinical cohorts are warranted to validate these findings and assess the broader applicability of sialic acid-Siglec-7-targeted immune modulation in reproductive medicine.

## Material and methods

### Tissue collection and ethical approval

Human first-trimester placental villi and decidual tissues were obtained from women undergoing elective terminations of pregnancy without special treatment at West China Second University Hospital, Sichuan University (Sichuan, China). Gestational weeks are estimated on the basis of the last menstrual period. All collections involving human tissue samples were approved by the Ethics Committee of West China Second University Hospital, Sichuan University (Ethics number: 2023077), and informed written consent from all donors was obtained under the supervision of West China Second University Hospital.

### Isolation and purification of human first–trimester dNK cells

Decidual tissues were washed extensively with cold PBS and minced into approximately 1 mm³ fragments. The tissue was enzymatically digested in 1 mg/mL collagenase IV (Sigma) and 50 μg/mL DNase I (Roche-sigma) at 37 °C in a shaking incubator (200 rpm, horizontal) for 45 min. The digested suspension was filtered sequentially through 150- and 350-mesh nylon strainers. The cells were pelleted by centrifugation at 400 rcf for 10 min at 4 °C. Mononuclear cells were isolated via Ficoll density gradient centrifugation (TBD). After incubation overnight in RPMI-1640 medium supplemented with 10% fetal bovine serum (FBS) and 1% antibiotic-antimycotic solution, nonadherent cells were collected. The isolated cells were centrifuged (400 rcf, 10 min, 4 °C), resuspended in PBS or culture medium, counted, and used for downstream applications.

### Isolation and culture of primary EVT cells

Placental villi were minced and digested using 1 mg/mL collagenase IV (Sigma) and 50 μg/mL DNase I (Roche–sigma) at 37 °C in a shaking incubator (200 rpm, horizontal) for 15 min. The cell suspension was filtered through 150- and 350-mesh nylon strainers and centrifuged at 800 rcf for 5 min. The pellet was resuspended in DMEM (Gibco). EVT cells were collected from the 15–30% and 30–45% phases. The cells were resuspended in DMEM containing 10% FBS and 1% antibiotic-antimycotic solution and plated onto culture dishes for 30 min at 37 °C to allow adherent cell removal. Nonadherent cells were then seeded onto 24-well plates precoated with Matrigel (Corning). After 24 h of incubation at 37 °C and 5% CO₂, the culture medium was replaced. EVT identity was confirmed by immunostaining for anti-cytokeratin 8 (CK8; Abcam), Vimentin (Abcam) and FITC-conjugated HLA-G (Abcam). Primary EVTs at approximately 80% confluence were passaged or cryopreserved for subsequent use.

### Cell culture

HTR-8/SVneo (HTR-8) cells (CVCL_7162; ATCC) were cultured in RPMI-1640 medium (Gibco). HEK-293FT cells (CVCL_6911; ATCC) were maintained in DMEM (Gibco). Both were incubated at 37 °C with 5% CO₂ supplemented with 10% FBS (Biochannel). NK-92MI cells (CVCL_3755; ATCC) were cultured in α-MEM (Gibco) containing 12.5% FBS (Gibco), 12.5% horse serum (Gibco), 0.02 mM folic acid (Sigma), 0.2 mM inositol (Sigma), and 0.1 mM 2-mercaptoethanol (Sigma) at 37 °C and 5% CO₂.

### Immunohistochemistry

Tissue microarray sections (3 μm thick) were deparaffinized and incubated with 3% hydrogen peroxide at room temperature for 10 min to block endogenous peroxidase activity. After antigen retrieval in citrate buffer, the slides were incubated overnight at 4 °C with primary antibodies against ST3GAL4 (Proteintech), ST6GALNAC6 (Proteintech), Siglec-7 (R&D Systems) or HLA-G (Santa Cruz Biotechnology), as well as with biotinylated lectins SNA and MALII (Vector Laboratories). The signals were visualized via 3,3′-diaminobenzidine (DAB), followed by counterstaining with hematoxylin.

### Real–time quantitative PCR

Total RNA was extracted via RNA isolation Total RNA Extraction Reagent (Vazyme), and 1 μg of RNA was reverse transcribed into cDNA via the PrimeScript RT Reagent Kit with gDNA Eraser (Takara) in a final volume of 20 μL. RT‒qPCR was performed via specific primers, which were used as internal controls. Relative expression levels were calculated via the 2^–(ΔΔCt)^ method.

### Western blotting and immunoprecipitation

The cells were lysed in RIPA buffer and centrifuged at 14,000 rpm for 10 min at 4 °C. Protein concentrations were determined, and equal amounts of protein were separated by SDS‒PAGE and transferred to PVDF membranes. The membranes were probed with antibodies against ST3GAL4 (Santa Cruz), ST6GALNAC6 (NOVUS), LAMP1 (Santa Cruz), CD151 (Santa Cruz), CD276 (Santa Cruz), ITGA2 (Santa Cruz), Siglec-7 (R&D Systems), phospho-STAT3 (Tyr705) (Cell Signaling Technology), STAT3 (Cell Signaling Technology), SHP-2 (Sigma), and GAPDH (ABclonal). For lectin blotting, biotinylated SNA and MAL II (Vector Laboratories) were used. The signals were detected via an enhanced chemiluminescence (ECL) imaging system, and the band intensities were semi-quantified via ImageJ.

Lectin precipitation was performed by incubating 500 μg of cell lysate with biotinylated SNA or MALII overnight at 4 °C, followed by enrichment via streptavidin magnetic beads (MCE) for 4 h. For co-immunoprecipitation, 100 μg of lysate was incubated with anti-Siglec-7 antibody (eBioscience) overnight at 4 °C, and immune complexes were captured via protein G Dynabeads (Invitrogen) for 2 h. After washing 3–5 times with PBST containing 0.02–0.05% Tween-20, the proteins were eluted by heating in loading buffer at 70 °C for 10 min and analyzed via 10% SDS‒PAGE.

### Flow cytometry

The cells were harvested and washed once with PBS, followed by fixation in 4% paraformaldehyde for 10 min at room temperature. After another PBS wash, cell surface sialylation was assessed by incubating the cells with 10 μg/mL biotinylated SNA or MAL II lectins (Vector Laboratories) for 2 h at room temperature, followed by a 1 h incubation at room temperature with Dylight 488-conjugated streptavidin (BioLegend). To evaluate Siglec-7 expression on dNK cells, the cells were stained on ice for 1 h with APC-conjugated anti-Siglec7 (BioLegend), APC-Cy7-conjugated anti-CD3 (BD Biosciences), AF700-conjugated anti-CD14 (BD Biosciences), BV421-conjugated anti-CD68 (BD Biosciences), BV510-conjugated anti-CD19 (BD Biosciences), and FITC-conjugated anti-CD56 (BioLegend) antibodies. After three washes with PBS, data acquisition was performed on a flow cytometer (BD Biosciences). For cell sorting, NK-92MI cells were stained with APC-conjugated anti-Siglec7 (BioLegend) and sorted into Siglec7⁺ and Siglec7⁻ populations via a BD Biosciences flow cytometer.

### Immunofluorescence

The cells cultured on coverslips were washed with cold PBS and fixed with 4% paraformaldehyde for 10 min at 4 °C. After being blocked with 1% BSA in PBS (w/v) at room temperature for 1 h, the cells were incubated overnight at 4 °C with anti-CK8 (Abcam), anti-Vimentin (Abcam) and FITC-conjugated anti-HLA-G (Abcam) antibodies. After washing with PBS, Alexa Fluor 594- and Alexa Fluor 488-conjugated secondary antibodies (Abcam) were applied for 1 h at room temperature, and the nuclei were counterstained with DAPI. Fluorescence images were captured using a Leica inverted fluorescence microscope.

### Transwell invasion assay

The in vitro invasive capacity of the cells was evaluated via 24-well transwell chambers with PET membranes precoated with Matrigel (50 μg/mL, Corning). Prior to seeding, HTR-8 cells were treated with 0.5 U of *C. perfringens* neuraminidase (NA; Sigma‒Aldrich) at 37 °C for 24 h to remove surface sialic acid. After treatment, HTR-8 cells were seeded at a density of 5 × 10⁴ cells per insert in the upper chamber using serum-free medium, whereas NK-92MI cells (1.5 × 10⁵) were placed in the lower chamber containing complete medium. After 24 h of incubation, the cells were fixed and stained with crystal violet. Non-invading cells on the upper surface of the membrane were removed with a cotton swab. The number of cells that had invaded through the membrane was counted on the lower surface under an optical microscope. Five random fields per sample were selected to calculate the average number of invaded cells. The number of invasive cells was counted via ImageJ. Each experiment was performed in triplicate, and the invasion index was calculated as the percentage of invaded cells normalized to the control.

### Wound healing assay

After 2.5 × 10⁴ HTR-8 cells were seeded, the culture was continued until the cells reached approximately 80% confluence. Then, NA (0.5 U) was added, and the cells were incubated for an additional 24 h. To assess cell migration, a linear scratch was introduced into a serum–starved monolayer of HTR-8 cells via a sterile pipette tip. The cells were rinsed with PBS and incubated in serum–free medium at 37 °C with 5% CO₂ for 24 h to allow migration. After the medium was removed, the cells were gently rinsed with PBS, and wound closure was imaged and quantified under an optical microscope. The wound healing area was determined via ImageJ.

### Plasmid construction

To generate lentiviral particles, the expression plasmids pLVX-Puro-*ST3GAL4*, pLVX-Puro-*SIGLEC7*, and pLVX-Puro-*ST6GALNAC6* were co-transfected with the packaging plasmids psPAX2 and pMD2. G into HEK-293FT cells. Viral supernatants were collected 48 h post-transfection and filtered through a 0.45 μm filter for subsequent use. The resulting viral supernatants were used to generate stable HEK-293FT cell lines overexpressing ST3GAL4, ST6GALNAC6 or Siglec-7. The efficiency of gene overexpression was validated by RT‒qPCR and western blotting.

### Cytokine assays

The supernatants were collected 24 h after coculture of HTR-8 cells and NK-92MI cells and stored at –80 °C until analysis. The levels of cytokines, including Fractalkine/CX3CL1, GM-CSF, IFNγ, IL-1β, IL-2, IL-4, IL-5, IL-6, IL-7, IL-8/CXCL8, IL-10, IL-12 (p70), IL-13, IL-17A/CTLA8, IL-21, IL-23, ITAC/CXCL11, MIP-1α/CCL3, MIP-1β/CCL4, MIP-3α/CCL20, and TNF-α, were simultaneously measured via the Human High Sensitivity T-Cell Panel (Millipore, HSTCMAG-28SK) according to the manufacturer’s instructions. The concentration of IL-8 in the coculture supernatants was also determined via a commercially available Human IL-8 ELISA Kit (Arigo, ARG83357). All the samples were analyzed in triplicate. The detection range for IL-8 was 23.4–1500 pg/mL.

### Membrane protein preparation

Membrane protein extraction was performed according to a previously published protocol [42]. The cells were detached via a cell scraper after being washed with D-PBS and collected via centrifugation at 1000 rcf for 3 min at 4 °C to obtain approximately 1 × 10⁶ cells. All buffers, including HB buffer, Na₂CO₃ solution, and Milli-Q water, were precooled to 4 °C. The preparation of reagents used for membrane protein extraction was performed according to a previously published protocol [42]. The cells were resuspended in 1.2 mL of HB buffer and lysed via ultrasonication (total time of 25 s). Nuclei and debris were removed by centrifugation at 2000 rcf for 10 min at 4 °C. The supernatant was transferred to a 15 mL ultracentrifuge tube and subjected to ultracentrifugation at 200,000 rcf for 45 min at 4 °C to pellet the membrane fractions. The pellet was resuspended in 0.2 M Na₂CO₃ to remove peripheral membrane proteins and centrifuged again at 200,000 rcf for 45 min at 4 °C. The resulting pellet was washed with Milli-Q water, mixed thoroughly, and ultracentrifuged once more under the same conditions. The final pellet was resuspended in 100–300 μL of Milli-Q water, and the protein concentration was measured. Samples with protein concentrations > 200 μg were aliquoted for downstream analysis.

### Glycoproteomic analysis

The protein concentration of the membrane proteins was measured via a BCA protein assay kit. After reduction with dithiothreitol and alkylation with iodoacetamide, digestion was performed with trypsin at a trypsin-to-protein ratio of 1:50 (w/w) (enzyme solution adjusted to 1 mg/mL) at 37 °C overnight. After digestion, the reaction was terminated, and desalting was performed using a Waters Sep-Pak 50 mg C18 column. Glycopeptides and other hydrophilic modified peptides were selectively enriched from the digested protein products via a HILIC column to remove non-glycopeptides or hydrophobic impurities. The enriched glycopeptides were redissolved in 0.1% FA (formic acid). All the samples were analyzed via a Thermo Scientific Orbitrap Eclipse™ Tribrid mass spectrometer. Mass spectrometry analysis of the complete glycopeptides was carried out in HCD-EthcD fragmentation mode in DDA (data-dependent acquisition) mode. The raw data files were searched via pGlyco 3.1, with the mass deviation for both the precursor and fragment ions set to ppm. The enzyme cleavage sites were R and K, with a maximum of three missed cleavages allowed. Fixed modifications were set to carbamidomethyl (C), and variable modifications were set to oxidation (M). The default human N-glycan and O-glycan databases were used. The precursor mass tolerance was set to 10 ppm, and the fragment mass tolerance was set to 20 ppm. Glycopeptides were filtered with an FDR < 1%. Quantification was performed via pGlycoQuant to assess the relative abundance of glycopeptides across different samples.

### Single-cell RNA sequencing

Single-cell RNA libraries were prepared via the 10× Genomics Chromium Single-cell 3′/5′ Library & Gel Bead Kit, which uses a microfluidics–based system to label scRNA-seq with unique molecular identifiers and barcodes. Libraries were sequenced on an Illumina platform in paired-end mode. A sequencing depth of ~40,000 reads per cell was achieved. The raw data were subjected to quality control via FastQC. Cell type annotation was performed by training a model via the trainSingleR function from the SingleR package and applying it to the dataset. Dimensionality reduction was performed via Garnett, and the cell types annotated via SingleR were projected onto UMAP for visualization. Differential expression analysis was carried out via the DesingleR package. Downstream analyses, including clustering and visualization of single-cell data, were conducted in R via the Seurat package (v4.3.0). Specific marker genes and differentially expressed genes were identified for each cluster via the Wilcoxon rank-sum test. Violin plots and feature plots were generated to illustrate gene expression patterns. Functional enrichment analyses were conducted via GeneCodis, applying a significance cutoff of *P* < 0.05. For genes of interest, heatmaps displaying their expression levels across cell clusters were generated via TBtools software (version 2.309).

### RNA sequencing

Total RNA was extracted via RNA isolation Total RNA Extraction Reagent (Vazyme, China), and RNA integrity was assessed via an Agilent 2100 Bioanalyzer. mRNA was fragmented and reverse-transcribed via random hexamer primers and M-MuLV reverse transcriptase to generate first-strand cDNA. RNA was removed by RNase H digestion, followed by second-strand synthesis via DNA polymerase I with dNTPs. cDNA ends were repaired, adenylated at the 3′ end, ligated with sequencing adapters, and size-selected. USER enzyme digestion was used to degrade the uracil-containing second strand before PCR amplification to generate the final library. Libraries were initially quantified via a Qubit 2.0 fluorometer and diluted to 1.5 ng/μL. Insert size was assessed via an Agilent 2100 Bioanalyzer. Libraries with an insert size within the expected range and an effective concentration >1.5 nM (determined by RT‒qPCR) were pooled for sequencing on the Illumina platform according to the target data yield. The raw sequencing data were subjected to quality control with FastQC (v0.11.9), and low-quality reads were filtered via Trimmomatic (v0.39). Clean reads were aligned to the human reference genome (hg38) via STAR aligner (v2.7.3a). Transcript assembly and quantification (FPKM) were performed via StringTie (v2.1.4). Differential expression analysis was conducted via DESeq2 (v1.30.1), with thresholds of adjusted *P* < 0.05 and | log₂ (fold change)|>1. KEGG pathway analysis and GSEA were used to assess whether there were significant differences in the expression trends of predefined gene sets between the two groups.

### Microfluidic Chip implantation Model construction

The microfluidic model was adapted primarily from the method described by Park et al. (2022) [20], with appropriate modifications for experimental requirements. Briefly, the chip microfluidic device was fabricated via standard soft lithography techniques. The PDMS (Sylgard, Dow Corning) base and curing agent were mixed at a 10:1 (w/w) ratio and poured onto a master mold containing SU-8 microstructures. The mixture was cured at room temperature overnight. After curing, the PDMS layer was peeled off from the master mold and perforated at both ends of the three–channel system via a 1 mm punch to create inlet and outlet channels. The upper and lower layers of the PDMS were subsequently treated with oxygen plasma and manually bonded to form a sealed channel system. To enhance the interface between the ECM hydrogel and the channel walls, the internal channels were incubated for 4 h at 37 °C with a 2.0 mg/mL polydopamine (PDA) solution (10 mM Tris-HCl buffer, pH 8.5). After washing with deionized water, the device was dried under UV light in a biosafety cabinet overnight.

The ECM hydrogel precursor solution was prepared by mixing Col-1 (8 mg/mL, Corning) and Matrigel (10 mg/mL, Corning) at a 1:1 (v/v) ratio under physiological pH conditions. The ECM precursor solution was injected into the central channel of the chip and incubated at 37 °C for 2 h to allow gelation. To prepare the hydrogel containing NK-92MI cells, the cells were suspended at a density of 7.5 × 10⁶ cells/mL in the ECM solution and injected into the central channel, where gelation occurred. The surface of the vascular channel was pretreated with fibronectin solution (0.1 mg/mL, Solarbio) to promote the attachment of HUVECs (1 × 10⁷ cells/mL). After cell injection, the device was rotated 90° every 10 min to ensure even cell coverage. EVT cells (1 × 10⁷ cells/mL) were subsequently injected into the fetal compartment, where the surface was not pretreated with ECM to facilitate cell attachment. After seeding, the device was tilted during culture to help EVT cells adhere to the hydrogel surface.

Cell labeling was performed by preincubating cells with CellTracker Green CMFDA (Thermo Fisher) and CellTracker Deep Red (Thermo Fisher) in DMEM containing 10% FBS for 30 min. During the chip culture, the reservoirs on both sides were filled with DMEM or F12 medium supplemented with 10% FBS, and the device was maintained under static culture conditions at 37 °C with 5% CO₂. The culture medium was replaced daily. Imaging was performed via an Olympus spinning disk confocal microscope.

### Sialyltransferase-mediated rescue of EVT invasiveness

To investigate whether exogenous sialyltransferase treatment could rescue EVT cell invasiveness, the cells were resuspended in DMEM supplemented with 10% FBS at a density of 1 × 10⁷ cells/mL. The recombinant sialyltransferases ST3GAL4 (R&D Systems) and ST6GALNAC6 (R&D Systems), both with confirmed enzymatic activity, were added to the suspension at final concentrations of 1 μg/mL and 0.5 μg/mL, respectively. The cells were incubated at 37 °C in 5% CO₂ for 24 h in the presence of the enzymes. After treatment, the medium was replaced with fresh DMEM containing 10% FBS but without recombinant enzymes, and the cells were further incubated under standard conditions prior to downstream assays. The control cells were cultured in parallel under identical conditions but without enzyme supplementation.

### Molecular modeling and protein–protein docking

Amino acid sequences of CD151, CD276, LAMP1, ITGA2, and Siglec-7 were obtained from the UniProt database. The predicted protein structures and interaction models were generated via the AlphaFold server (https://alphafoldserver.com). Protein‒protein interaction interfaces were visualized with PyMOL (v3.0.4, Schrödinger, New York, USA), with key binding sites highlighted to illustrate potential contact regions.

### Data analysis

All the data were analyzed via GraphPad Prism (version 9.5) unless otherwise specified. Statistical comparisons between two groups were performed via unpaired two-tailed Student’s *t* tests, and comparisons among multiple groups were performed via one-way or two-way ANOVA followed by appropriate post hoc tests. A *p* < 0.05 was considered statistically significant. The data are presented as the means ± standard deviations (SDs) or standard errors of the means (SEMs), as indicated in the figure legends. Figures were generated via GraphPad Prism and Adobe Photoshop (version 25.2).

## Supplementary information

Supplementary Figures and Tables

Uncropped gels and blots

Original Western blotting image

List of antibodies and reagents

List of differential glycoproteins

## Data Availability

The raw sequencing data reported in this paper have been deposited in the Genome Sequence Archive in the National Genomics Data Center, Beijing Institute of Genomics, Chinese Academy of Sciences/China National Center for Bioinformation (Single-cell transcriptome data: GSA: HRA011899 with BioProject: PRJCA041616), (Transcriptome data: GSA: HRA012750 with BioProject: PRJCA041677) and are publicly accessible at http://bigd.big.ac.cn/gsa-human and https://ngdc.cncb.ac.cn/bioproject/.
