# Supplementary material for: Deficient extravillous trophoblast invasion caused by impaired sialylation–Siglec-7 interaction contributes to recurrent pregnancy loss
Source: Cell Death Dis. 2026 Mar 2;17(1):291. doi: 10.1038/s41419-026-08503-9 (PMC13031383; doi:10.1038/s41419-026-08503-9)
Supplement: Supplementary file 1 — Supplementary Figures and Tables [file 41419_2026_8503_MOESM1_ESM.doc]

**Supplementary Figures and Tables**

**
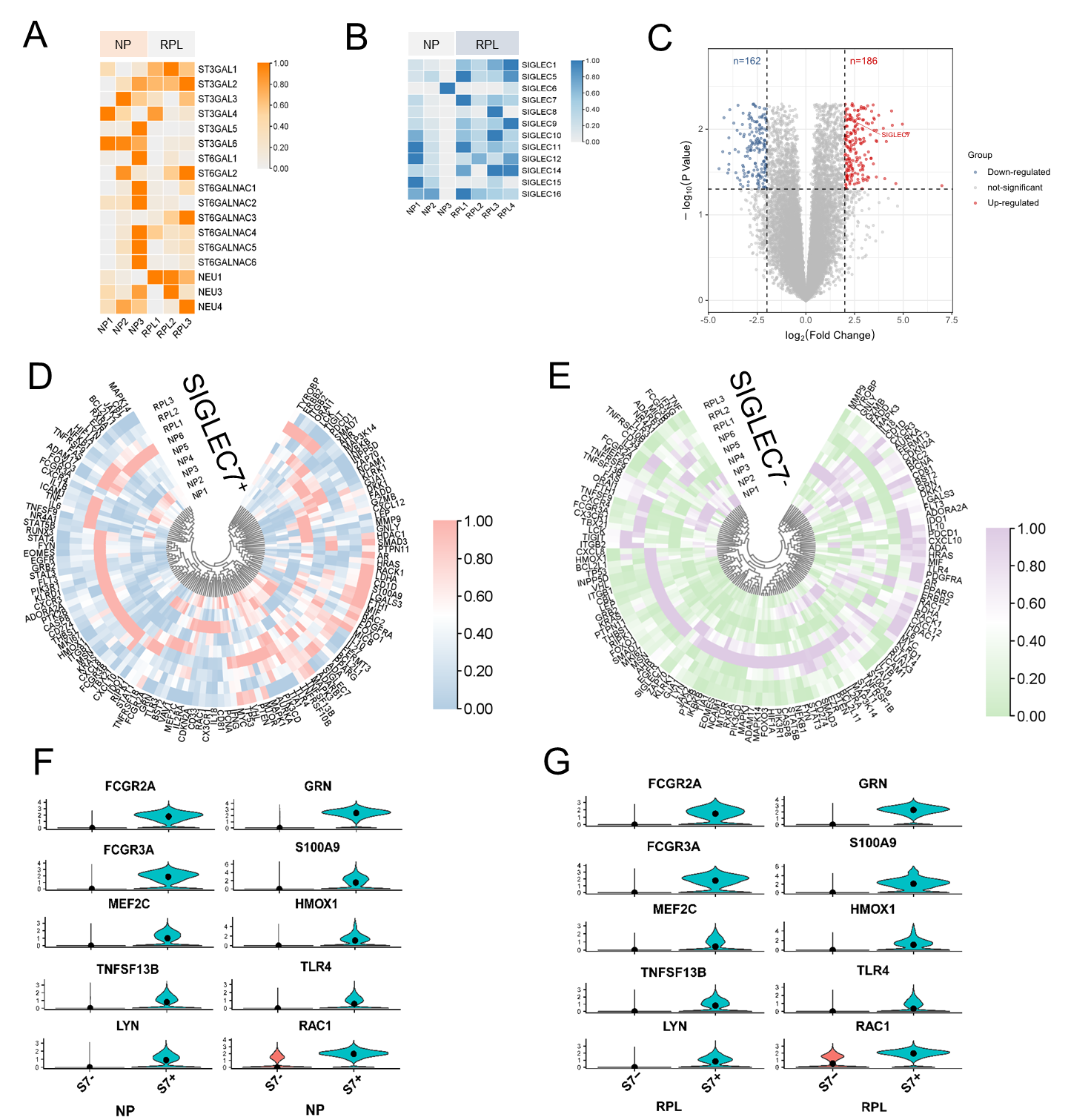
**

**Fig. S1.** Transcriptomic alterations of sialylation-related genes and Siglec family members in RPL. **A** Heatmap showing differential expression of sialyltransferases and sialidases in chorionic villi from RPL and NP groups (bulk RNA-seq: GSE121950). **B** Heatmap showing differential expression of Siglec family members in decidual tissues from RPL and NP groups (bulk RNA-seq: GSE161969). **C** Volcano plot highlighting differentially expressed genes between RPL and NP groups, with *SIGLEC7* indicated. **D, E** Heatmaps showing immune-related gene expression differences between *SIGLEC7*⁺ and *SIGLEC7*⁻ dNK cell subsets in NP and RPL (scRNA-seq). **F, G** Violin plots comparing immune-related genes between *SIGLEC7*⁺ and *SIGLEC7*⁻ dNK cell subsets in NP and RPL samples.

**
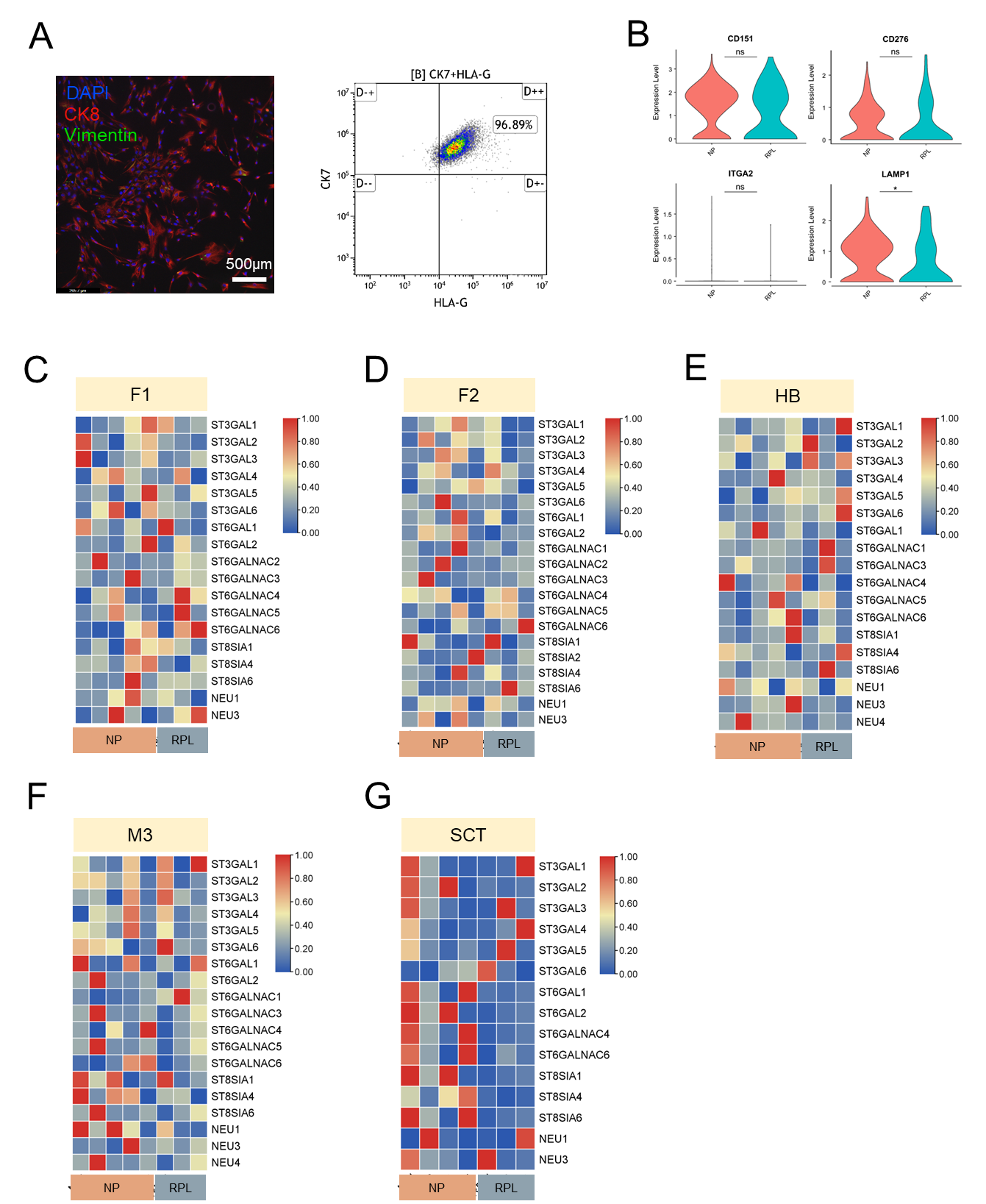
**

**Fig. S2.** Differential sialyltransferase expression across placental cell types. **A** Purity assessment of primary EVT cells. **B** Violin plots show the expression of CD151, CD276, ITGA2, and LAMP1 in EVT cells from NP and RPL samples (scRNA-seq). **C–G** Heatmap showing differential expression of sialyltransferases and sialidases in F1, F2, HB, M3 and SCT cell from RPL and NP groups (scRNA-seq). F1 (fibroblasts 1), F2 (fibroblasts 2), HB (Hofbauer cells), M3 (maternal macrophages), SCT (syncytiotrophoblast). Box plots: the centerline represents the median; boxes span the interquartile range (IQR), from the 25th to 75th percentiles; whiskers extend to the most extreme values within 1.5×IQR; data points beyond this range are shown as outliers. *P < 0.05, ns, not significant.

**
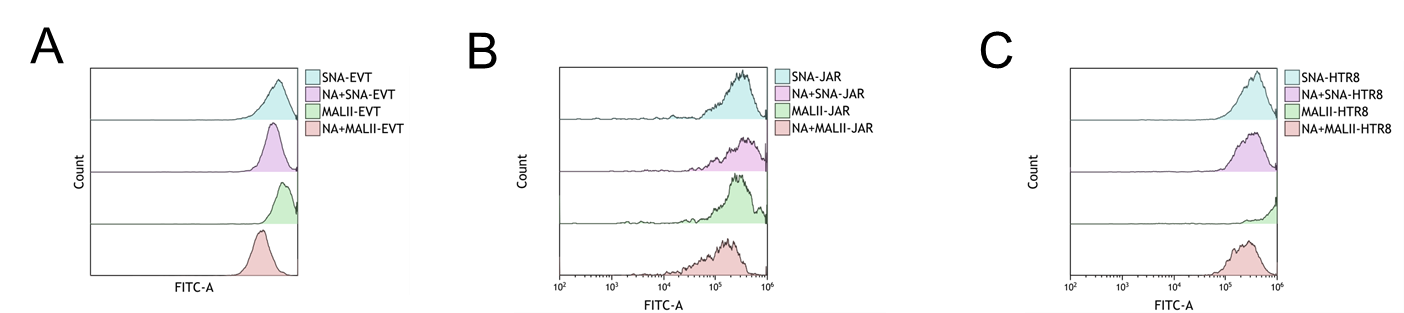
Fig. S3.** Assessment of cell surface sialic acid after neuraminidase (NA) treatment. **A–C** Flow cytometry analysis of surface sialylation using lectin staining in NA-treated EVT (A), JAR (B), and HTR-8 (C) cells.

**
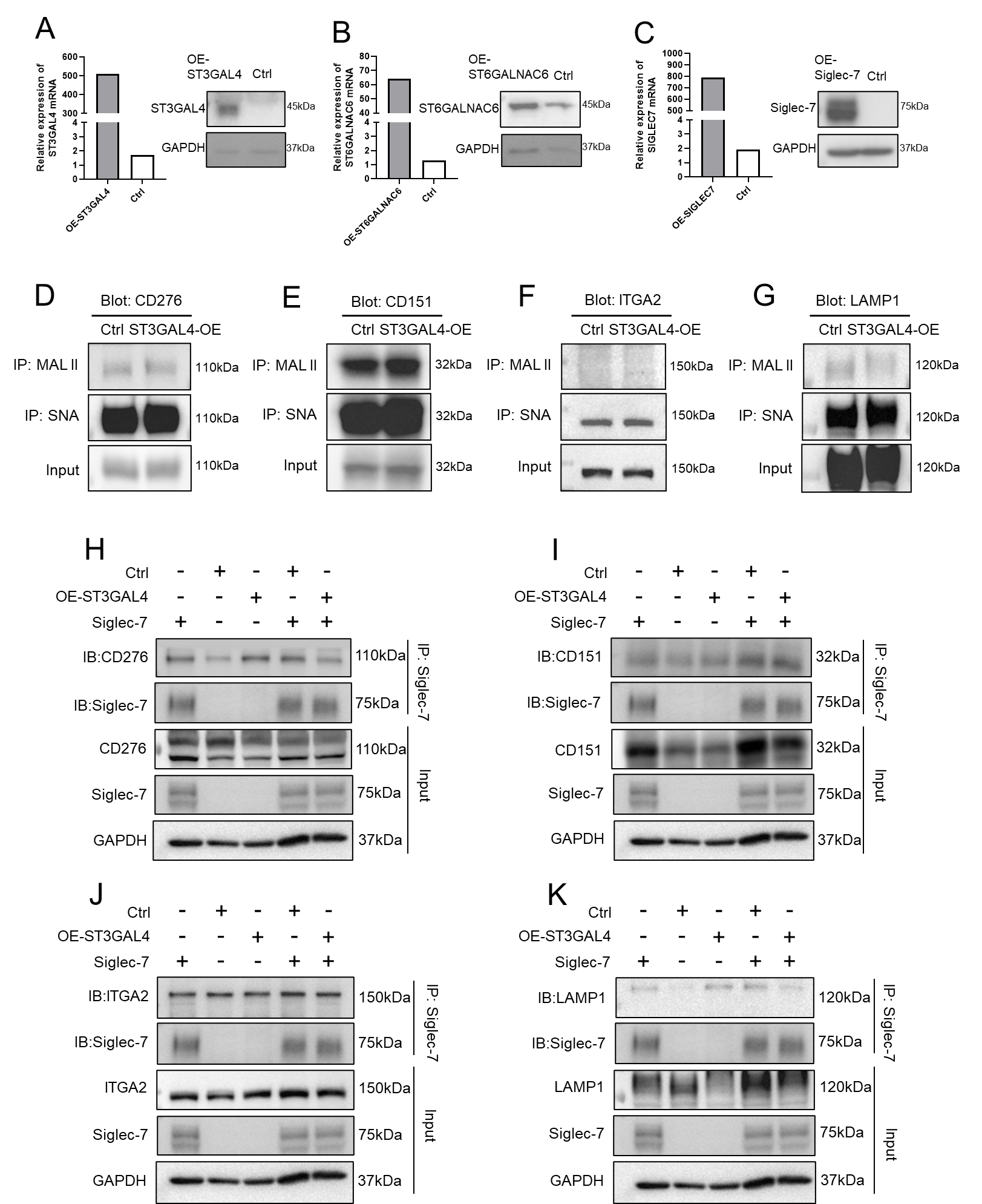
**

**Fig. S4.** Impacts of glycosyltransferase overexpression on sialylation and Siglec-7 binding. **A–C** RT-qPCR and Western blot confirming stable overexpression of ST3GAL4 (A), ST6GALNAC6 (B), and Siglec-7 (C) at mRNA and protein levels. **D–G** Levels of surface sialylation on CD276 (D), CD151 (E), ITGA2 (F), and LAMP1 (G) in ST3GAL4-overexpressing HEK-293FT cells. **H–K** The interaction of CD276, CD151, ITGA2, and LAMP1 with Siglec-7 was compared between ST3GAL4-overexpressing and control groups using co-immunoprecipitation.**
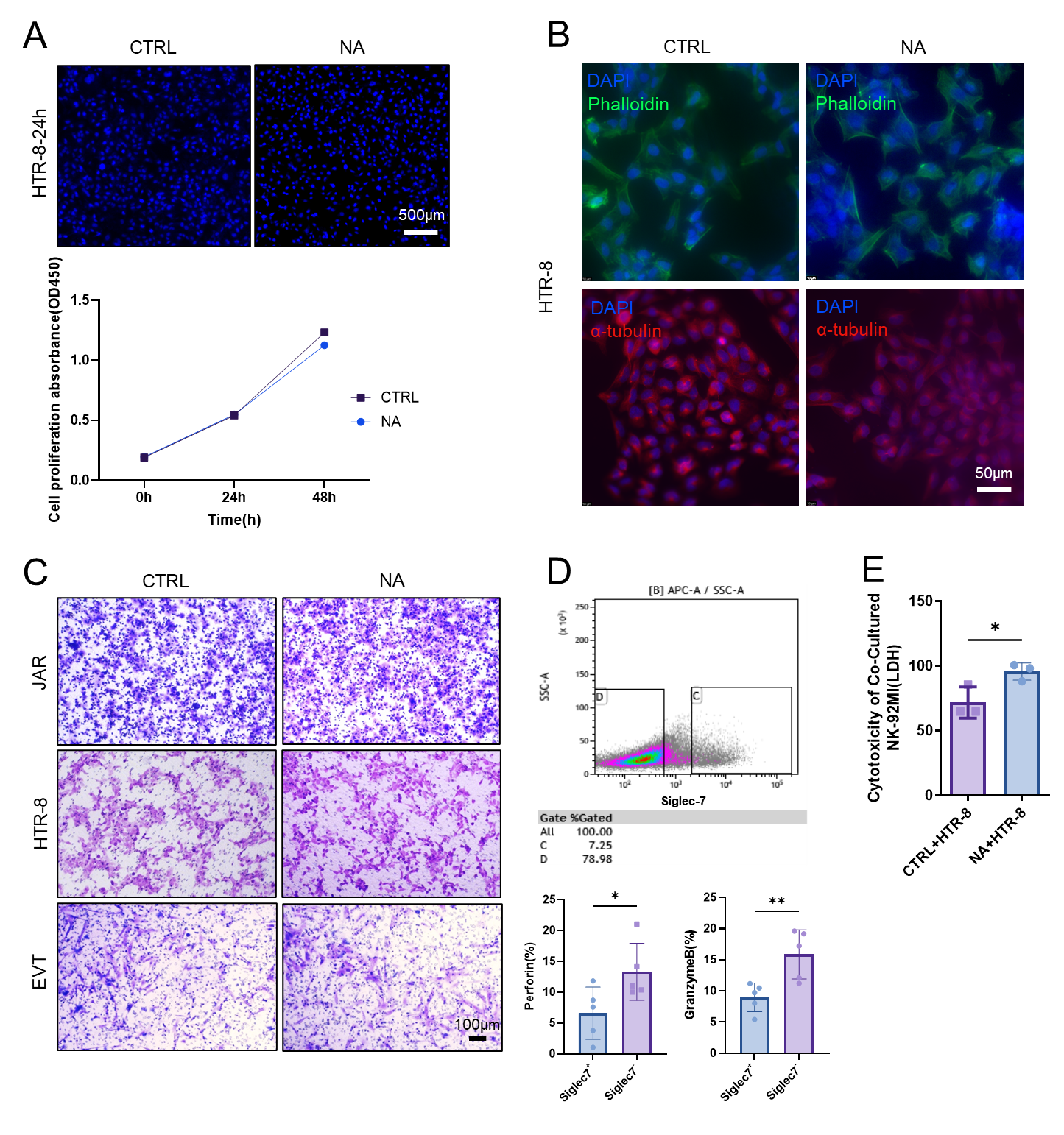
**

**Fig. S5.** Functional assays for NA-treated trophoblasts and NK cytotoxicity. **A** DAPI staining and CCK-8 assay showing comparable growth trends between NA-treated and control HTR-8 cells at 0 h and 24 h. **B** Phalloidin and α-tubulin staining showing no observable cytoskeletal differences between NA-treated and control HTR-8 cells. **C** Invasion assays showing no significant changes in invasive capacity of EVT, JAR, and HTR-8 cells following NA treatment. **D** Siglec-7–based flow cytometric sorting of NK-92MI cells into Siglec-7⁺ and Siglec-7⁻ populations; flow cytometry showing higher perforin and granzyme B expression in Siglec-7⁻ cells. **E** LDH cytotoxicity assay showing enhanced NK-92MI-mediated cytotoxicity toward NA-treated HTR-8 cells. Statistical significance was determined via a two-tailed Student’s *t*-test. *P < 0.05, **P < 0.01.

**
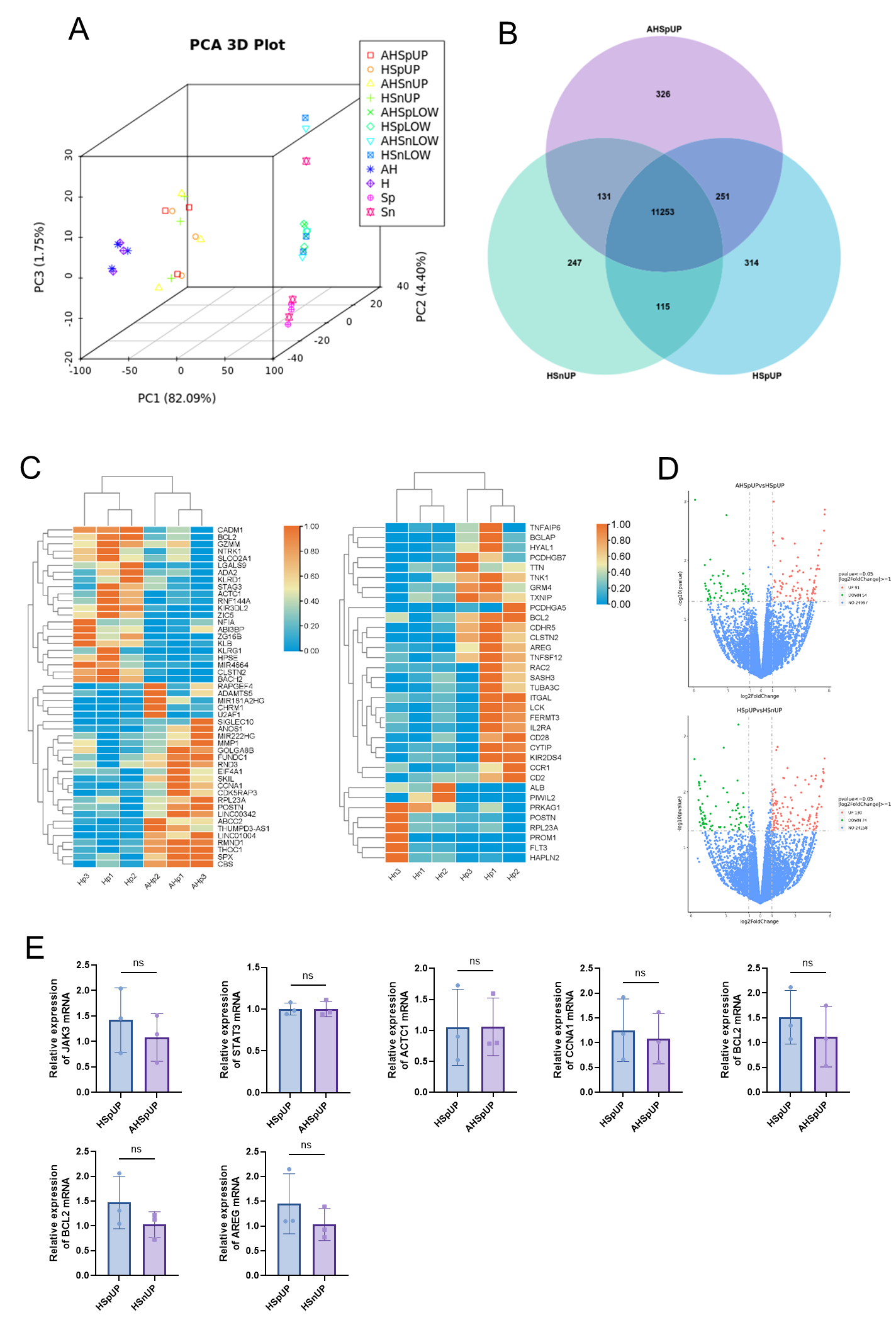
**

**Fig. S6.** Transcriptomic responses to Sia-Siglec-7 modulation in co-culture system**. A** Principal component analysis of transcriptomic profiles across experimental groups. **B** Venn diagram showing shared and distinct genes among HTR-8-derived groups. **C, D** Heatmap (C) and volcano plots (D) showing differentially expressed genes between AHSp vs HSp and HSp vs HSn comparisons. **E** RT-qPCR validation of selected differentially expressed genes. Group definitions: AHSp: NA-treated HTR-8 co-cultured with Siglec-7⁺ NK-92MI; HSp: HTR-8 co-cultured with Siglec-7⁺ NK-92MI; AHSn: NA-treated HTR-8 co-cultured with Siglec-7⁻ NK-92MI; HSn: HTR-8 co-cultured with Siglec-7⁻ NK-92MI. "UP": HTR-8 cells in upper transwell chamber; "LOW": NK-92MI cells in lower chamber. Statistical significance was determined via a two-tailed Student’s *t*-test. ns, not significant.

**
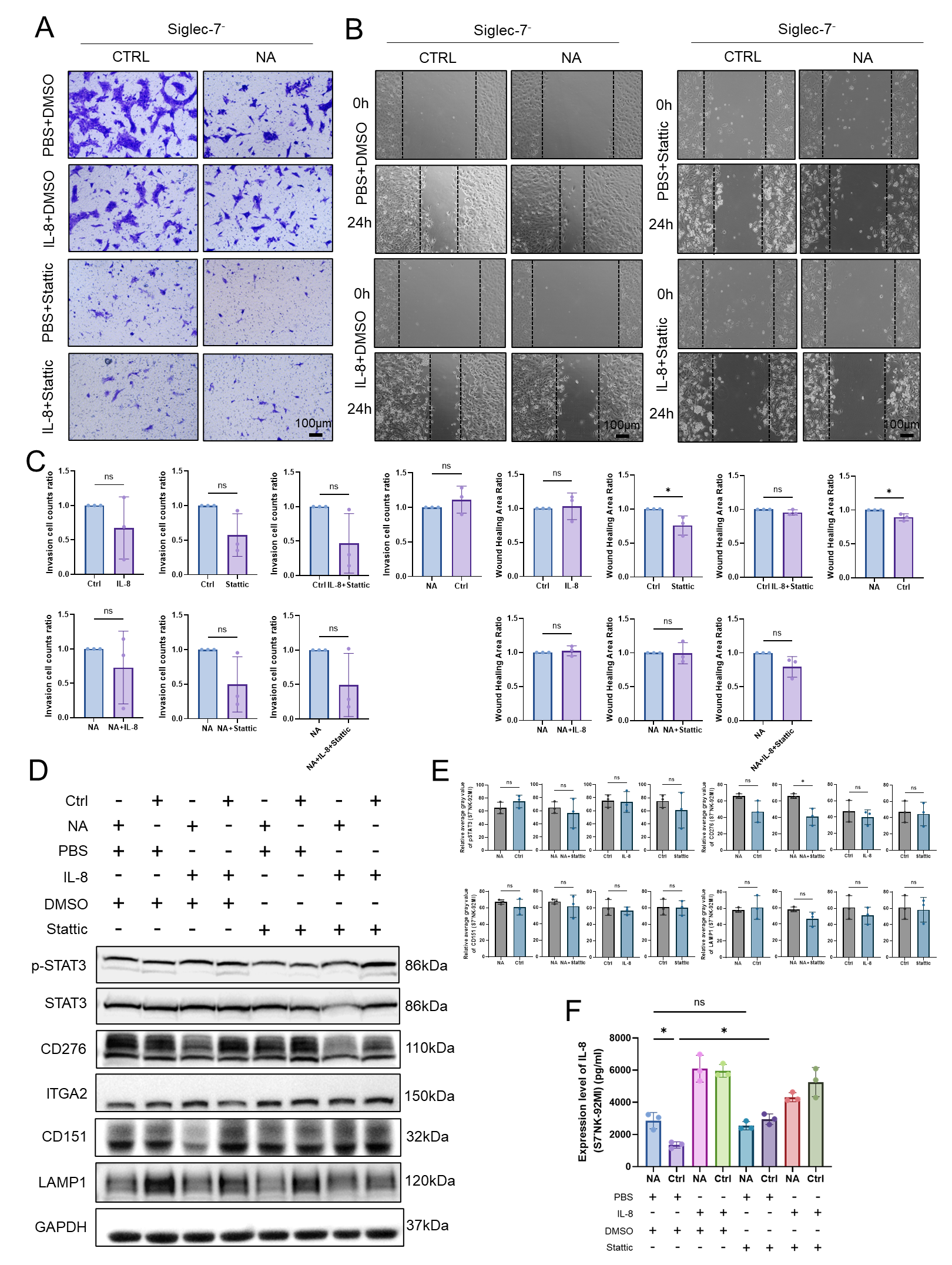
**

**Fig. S7.** HTR-8 cell migration, invasion, and IL-8–STAT3 signaling during co-culture with Siglec-7⁻ NK-92MI cells. **A** Transwell invasion assay showing the number of HTR-8 cells (5×10⁴) that migrated through the membrane toward Siglec-7⁻ NK-92MI cells (1.5×10⁵) after treatment with PBS+DMSO, IL-8+DMSO, PBS+Stattic, or IL-8+Stattic. **B** Scratch assay showing wound area at 0 h and 24 h in NA-treated HTR-8 cells (2.5×10⁴ in the lower chamber), following co-culture with Siglec-7⁻ NK-92MI cells (7.5×10⁴ in the upper chamber) and the same four treatments. **C** Quantification of invasive cells and wound healing areas using ImageJ. No significant differences observed among IL-8, Stattic, or combined treatment groups in the presence of Siglec-7⁻ NK-92MI cells. **D, E** Western blot showing expression levels of p-STAT3, CD276, CD151, and LAMP1 in HTR-8 cells under the same treatment conditions. No significant differences detected between groups. Statistical significance determined by two-tailed Student’s *t*-test: *P < 0.05, ns: not significant. **F** ELISA quantification of IL-8 in supernatants under different treatments. In NA-treated cells, no significant IL-8 changes observed between Stattic and control; in untreated control cells, Stattic increased IL-8 levels. Data shown as mean ± SD, analyzed using one-way ANOVA. *P < 0.05; ns: not significant.

**
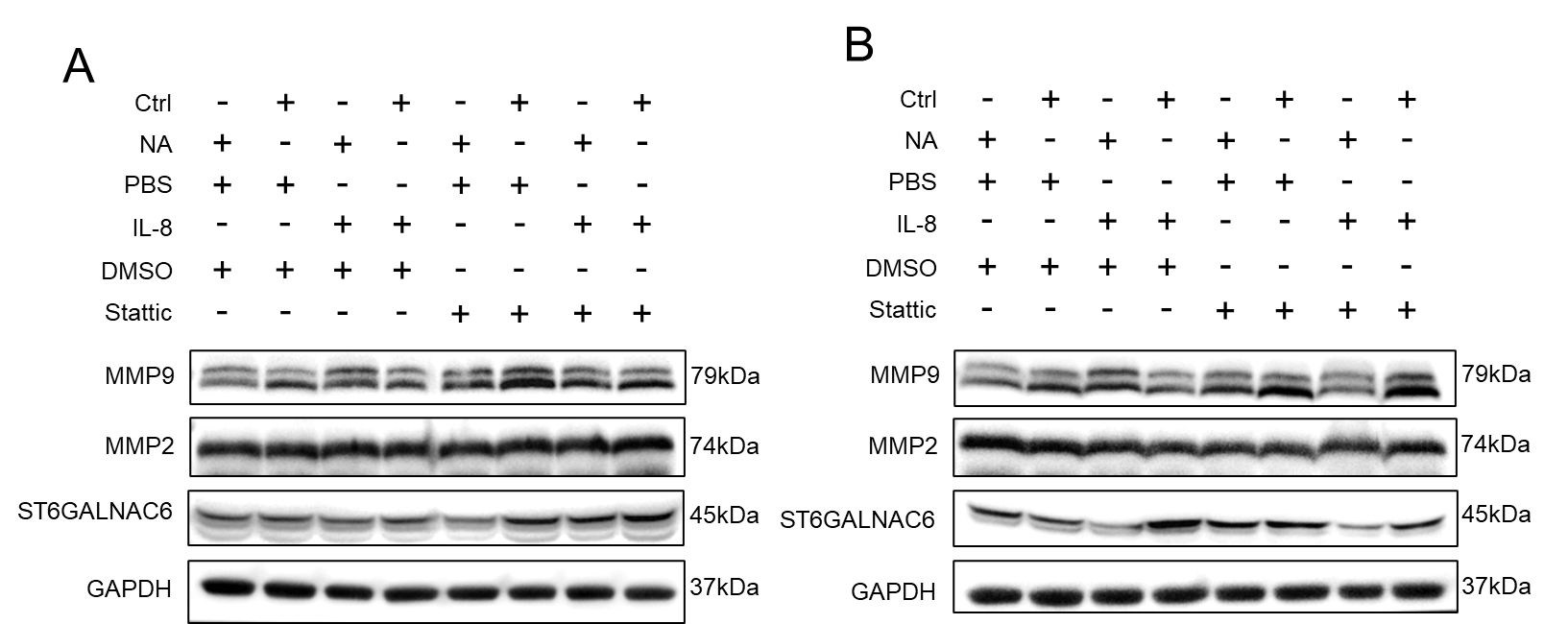
**

**Fig. S8.** HTR-8 co-culture with NK-92MI cells. **A-B** Western blot analysis showed the expression levels of MMP9, MMP2, and ST6GALNAC6 in HTR-8 cells co-cultured with Siglec-7⁺ NK-92MI cells (A) and Siglec-7⁻ NK-92MI cells (B) under the treatment conditions of PBS+DMSO, IL-8+DMSO, PBS+Stattic, or IL-8+Stattic.


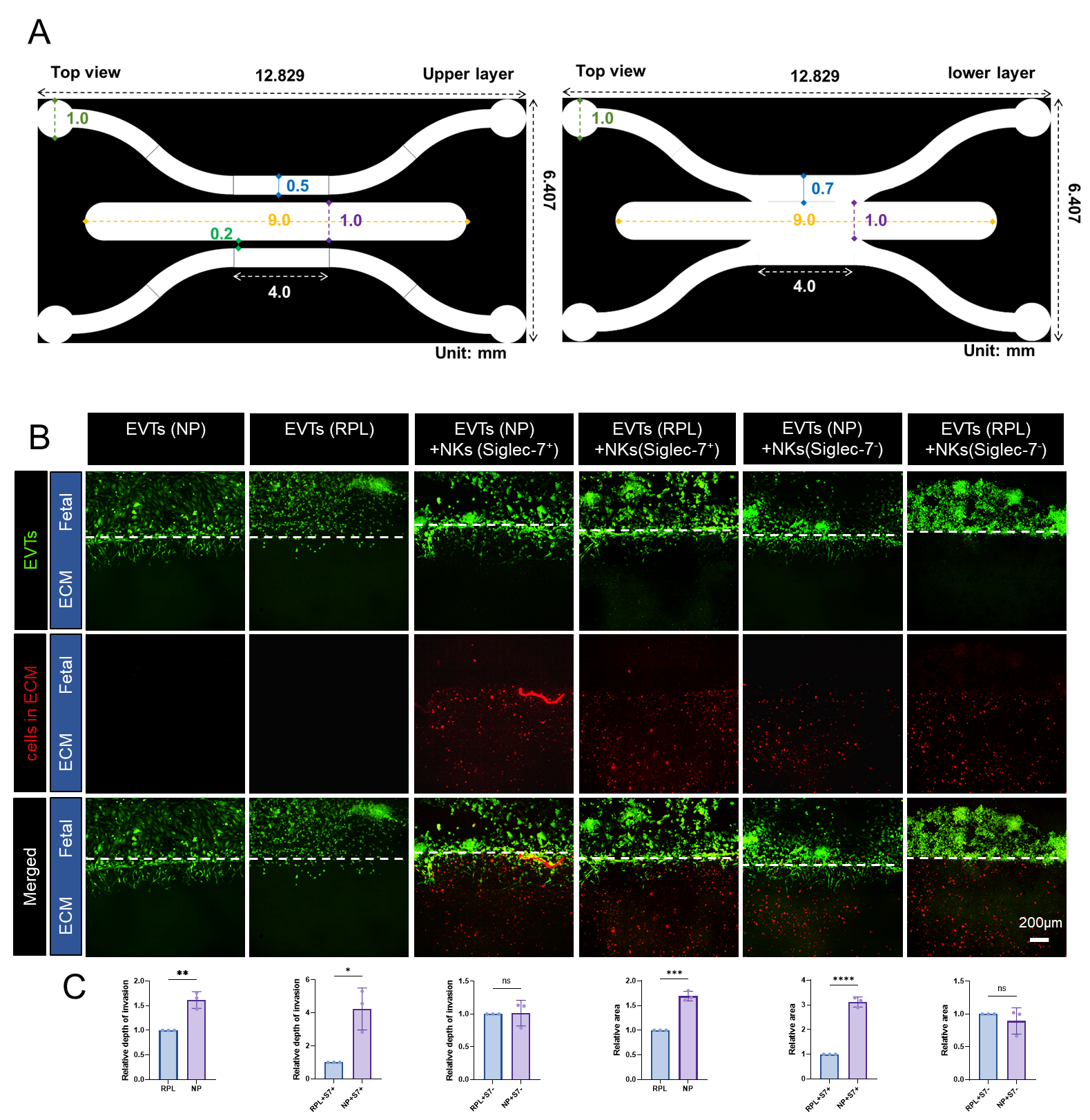


**Fig. S9.** Evaluation of EVT invasion in RPL and NP samples using an organ-on-a-chip model. **A** Schematic illustration of the upper and lower layers of the microfluidic implantation chip. **B** Invasion of EVT cells derived from NP and RPL samples was compared, both in the presence and absence of NK-92MI cells in the matrix. **C** Quantification of invasion depth and area using Olympus software showed that RPL-derived EVT cells exhibited significantly reduced invasiveness compared to NP-derived cells. Statistical significance was assessed using Student’s two-tailed t-test; *P < 0.05, **P < 0.01, ***P < 0.001, ****P < 0.0001, ns, not significant.

**
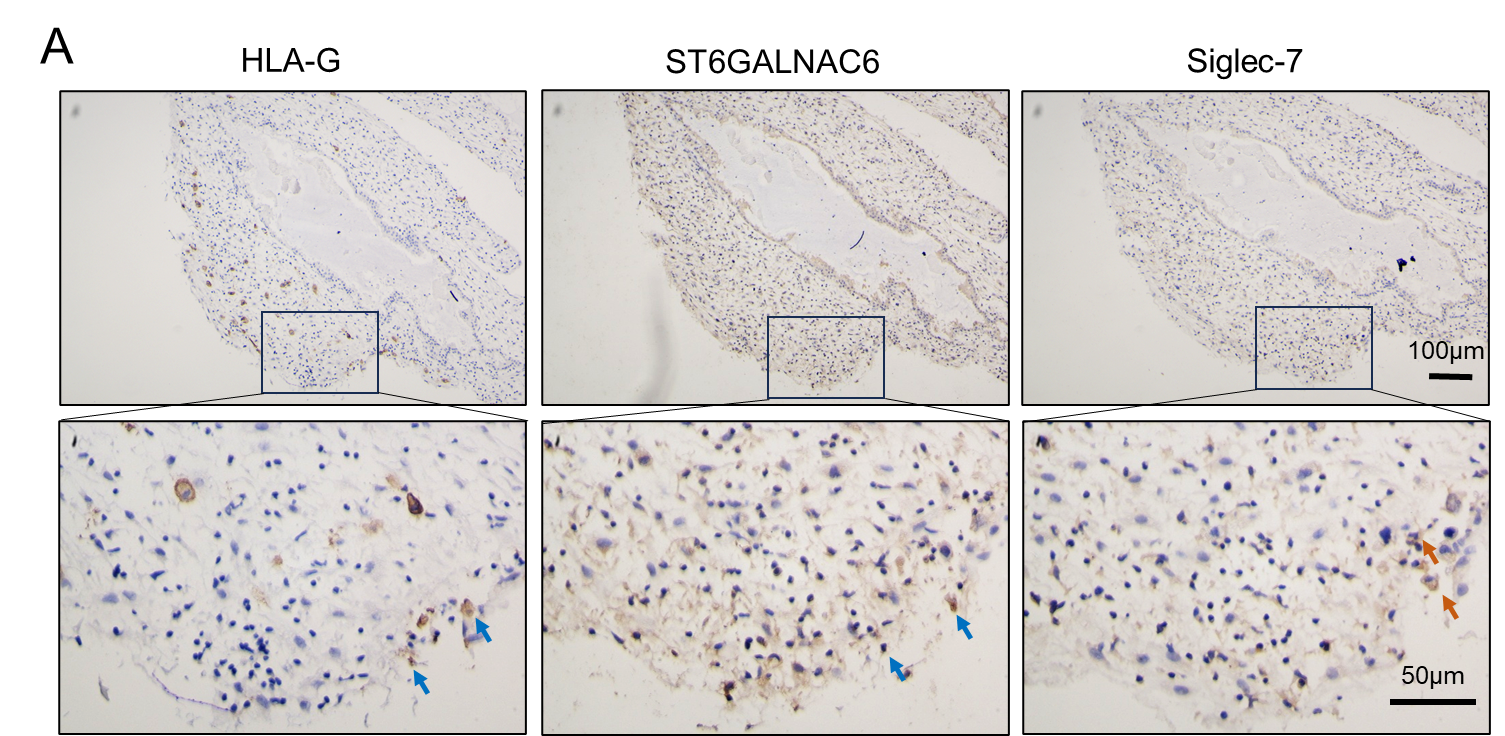
**

**Fig. S10.** Immunohistochemical staining of decidual tissues. Serial sections of decidual tissue were stained to identify spatial relationships among different cell types. The first section shows HLA-G staining to mark EVT cells, the second displays ST6GALNAC6 expression in EVT cells, and the third demonstrates Siglec-7 expression in decidual immune cells. The combined analysis of these serial sections reveals that ST6GALNAC6-expressing EVTs are spatially adjacent to Siglec-7 positive immune cells.

**
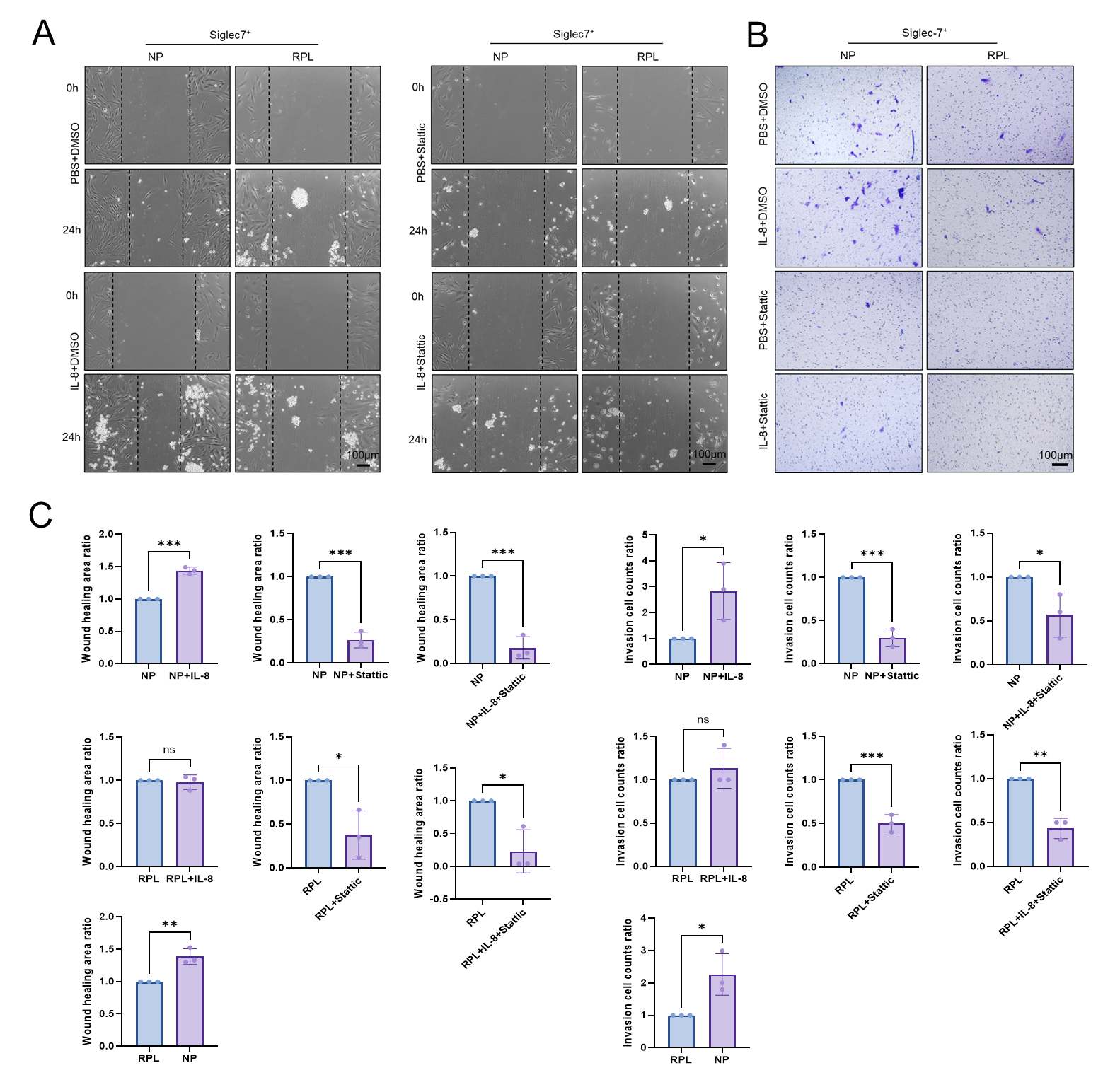
**

**Fig. S11.** EVT cell migration, invasion, and IL-8–STAT3 signaling during co-culture with Siglec-7⁺ NK-92MI cells. **A** Scratch assay showing the wound area at 0 h and 24 h in RPL-EVT cells (2.5×10⁴ in the lower chamber) co-cultured with Siglec-7⁺ NK-92MI cells (7.5×10⁴ in the upper chamber) under the same four treatments (PBS+DMSO, IL-8+DMSO, PBS+Stattic, or IL-8+Stattic). **B** Transwell invasion assay showing the number of EVT cells (5×10⁴) that migrated through the membrane toward Siglec-7⁺ NK-92MI cells (1.5×10⁵) after treatment with PBS+DMSO, IL-8+DMSO, PBS+Stattic, or IL-8+Stattic. **C** Quantification of invasive cells and wound closure areas using ImageJ. IL-8 enhanced the migration and invasion of EVT cells but failed to promote these abilities in RPL-EVTs, whereas Stattic or combined IL-8 treatment significantly inhibited EVT cell migration and invasion in the presence of Siglec-7⁺ NK-92MI cells. Statistical significance was assessed using Student’s two-tailed *t*-test; *P < 0.05, **P < 0.01, ***P < 0.001, ns, not significant.


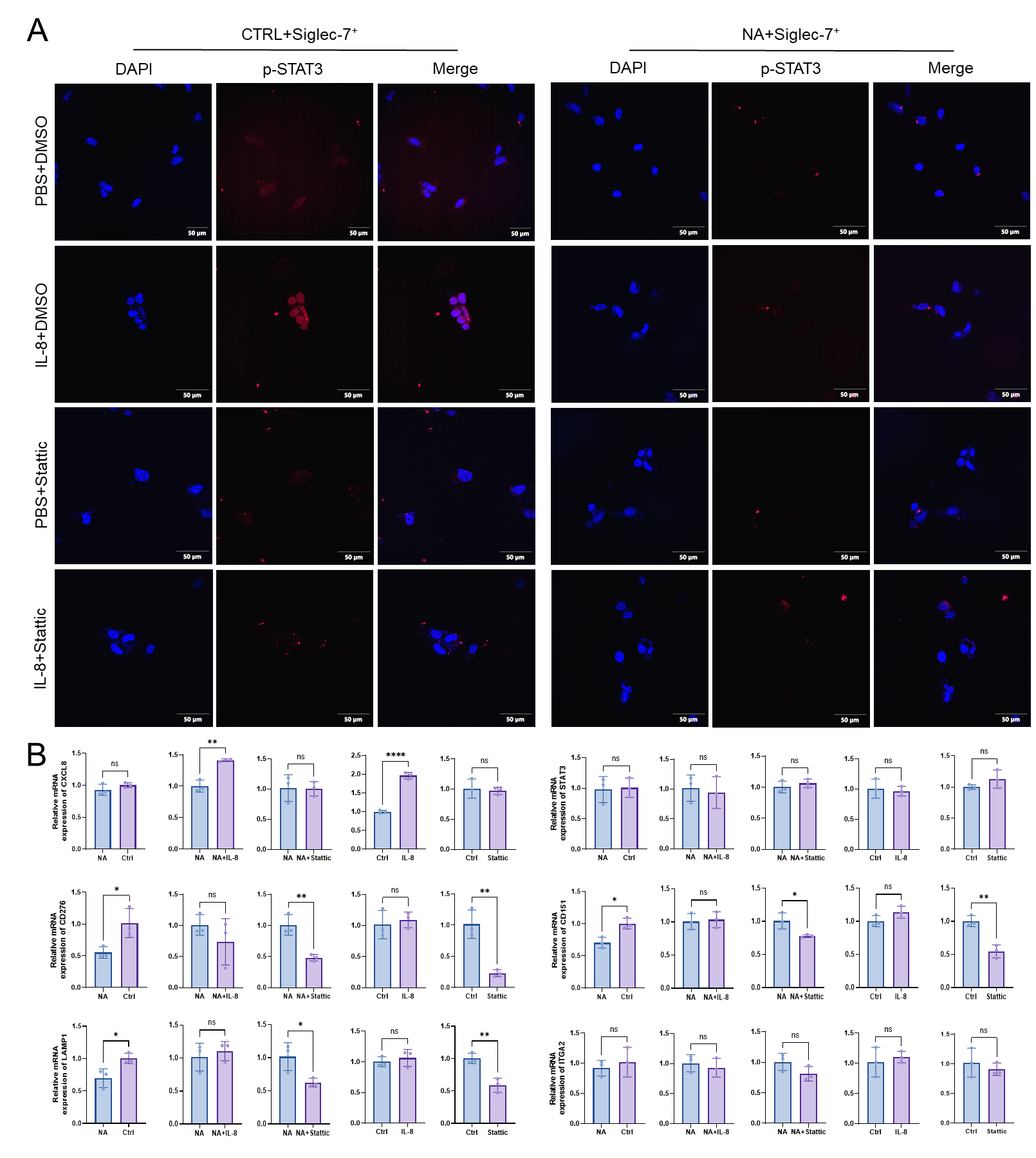


**Fig. S12.** Immunofluorescence staining and mRNA expression in HTR-8 cells after different treatments. **A** HTR-8 cells (5×10⁴) grown on coverslips were treated with PBS+DMSO, IL-8+DMSO, PBS+Stattic, or IL-8+Stattic, and co-cultured with Siglec-7⁺ NK-92MI cells (1.5×10⁵) seeded in the upper chamber of a Transwell insert. Cells were stained for p-STAT3 to assess phosphorylation levels. **B** HTR-8 cells (5×10⁵) were seeded in the upper chamber and Siglec-7⁺ NK-92MI cells (1.5×10⁶) in the lower chamber of a Transwell system under the same treatments. RT–qPCR was performed to measure mRNA levels of *STAT3*, *CD276*, *CD151*, *ITGA2* and *LAMP1* in HTR-8 cells, and *CXCL8* in Siglec-7⁺ NK-92MI cells. Statistical significance was assessed using Student’s two-tailed *t*-test; *P < 0.05, **P < 0.01, ****P < 0.0001, ns, not significant.

**Table S1. The clinical characteristics of pregnant women participating in this study**

|  | NP | RPL | P value |
| --- | --- | --- | --- |
| Total sample | 16 | 13 |  |
| Maternal age (year) | 31.75±3.13 | 32.69±3.64 | ns |
| Gestational age (day) | 51.75±6.88 | 53.77±7.07 | ns |
| Number of spontaneous abortions | n/a | 3.09±1.6 | n/a |

Two-tailed unpaired Student’s *t* test. Data are mean ± SEM. ns, not significant.

**Table S2. Primer list of constructing vector**

| Gene | Primer |
| --- | --- |
| hST3GAL4-Forward | gagctcaagcttcgaattATGGTGGCCCGAGGC |
| hST3GAL4-Reverse | cggtaccgtcgactgcagTCAGAAGGACGTGAGGTTCTTG |
|  |  |
| hST6GALNAC6-Forward | gagctcaagcttcgaattATGGCTTGCTCGAGGCC |
| hST6GALNAC6-Reverse | cggtaccgtcgactgcagCTAGGTCCAGGAGGGGTGG |
|  |  |
| hSIGLEC7-Forward | gagctcaagcttcgaattATGCTGCTGCTGCTGC |
| hSIGLEC7-Reverse | cggtaccgtcgactgcagTTACTTGGGGATCTTGATCTCTGAGTACTC |

**Table S3. Primer list of RT–qPCR**

| Gene | Forward Primer | Reverse Primer |
| --- | --- | --- |
| *ST3GAL4* | CCACTTCGACCCCAAAGTAGA | AGTGATAAGAAGCGGGTGCG |
| *ST6GALNAC6* | ATGGCTTGCTCGAGGCC | CTAGGTCCAGGAGGGGTGG |
| *SIGLEC7* | AAACACCATCAGGGGCTCAG | CCATGGTGTCGGGGGTTATC |
| *CXCL8* | GTGCAGTTTTGCCAAGGAGT | ATGAATTCTCAGCCCTCTTCAA |
| *ACTC1* | CAGCTAAGCGTGGTCCGC | CTTGCTCTGGGCTTCATCAC |
| *AREG* | CGAAGGACCAATGAGAGCCC | TTGAGGTCCAATCCAGCAGC |
| *BCL2* | AAAAATACAACATCACAGAGGAAGT | AGGGGGTGTCTTCAATCACG |
| *CCNA1* | GATAACGACGGGAAGAGCGG | CGGTCTCCATCCCAAGTGAC |
| *JAK3* | CCCTTCGAAAGTCCAGGGTC | GATCAGGGGCGTCTCTTCAC |
| *STAT3* | AACAGGATGGCCCAATGGAA | GAAGCGGCTATACTGCTGGT |
| *GAPDH* | AACTTTGGTATCGTGGAAGG | GCCAGTAGAGGCAGGGATGA |
| *CD276* | ACAGCTGGTGCACAGCTTTGCTGA | GTGCACATCAAACAAGCCCTGCTC |
| *CD151* | CCTAGAGTCCTGGGGAGCTT | GTACTTGAGGCAAACGGTG |
| *ITGA2* | TCCAGAGTAACCTCCAGGGG | CAAAGTCCGTCCTCACCACA |
| *LAMP1* | CTGGTAACGCCGCTGTCTCT | TAAACATTGCTGCTGACGCAC |
