# Supplementary material for: Deficient extravillous trophoblast invasion caused by impaired sialylation–Siglec-7 interaction contributes to recurrent pregnancy loss
Source: Cell Death Dis. 2026 Mar 2;17(1):291. doi: 10.1038/s41419-026-08503-9 (PMC13031383; doi:10.1038/s41419-026-08503-9)
Supplement: Supplementary file 2 — Uncropped gels and blots [file 41419_2026_8503_MOESM2_ESM.pdf]

Fig. 2C

ST6GALNAC6

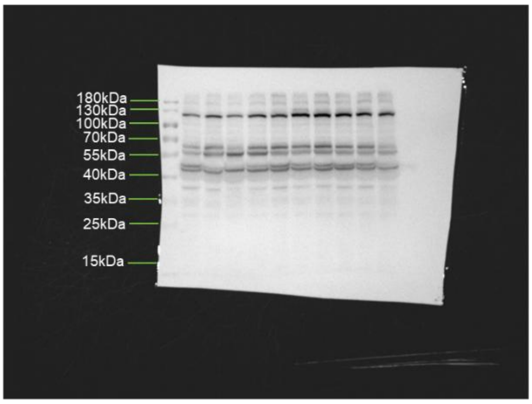

ST3GAL4

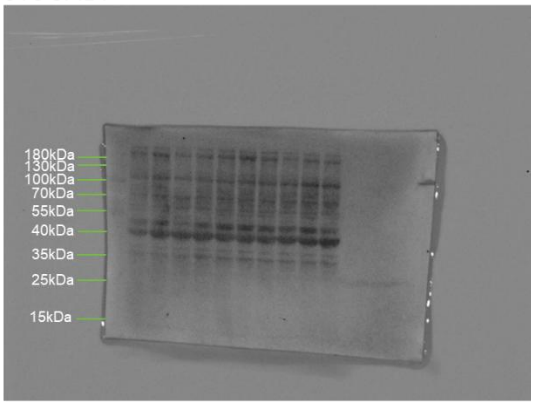

GAPDH

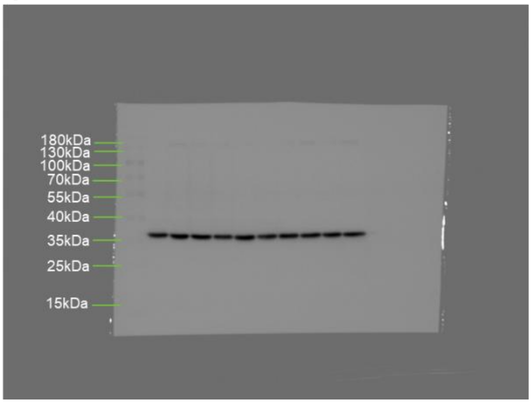

Figure 2-source data 1
Original blots of Figure 2C.

Fig. 2D

MAL II

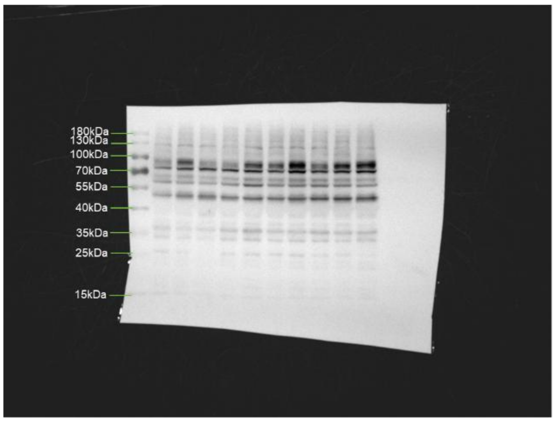

SNA

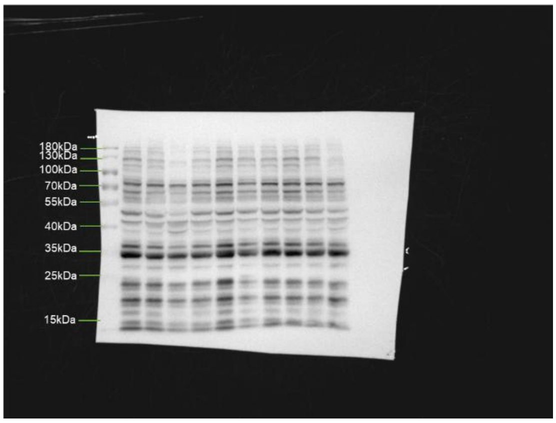

Figure 2-source data 1
Original blots of Figure 2D.

Fig. 4E-H

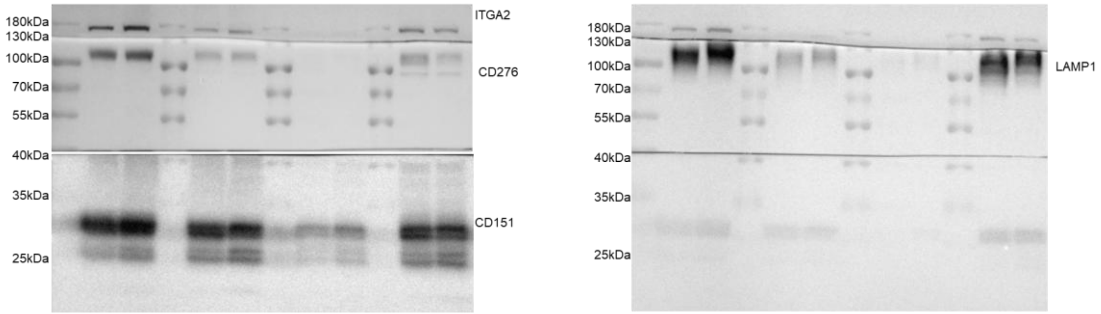

Figure 4-source data 1

Original blots of Figure 4E-H.

Fig. 4E

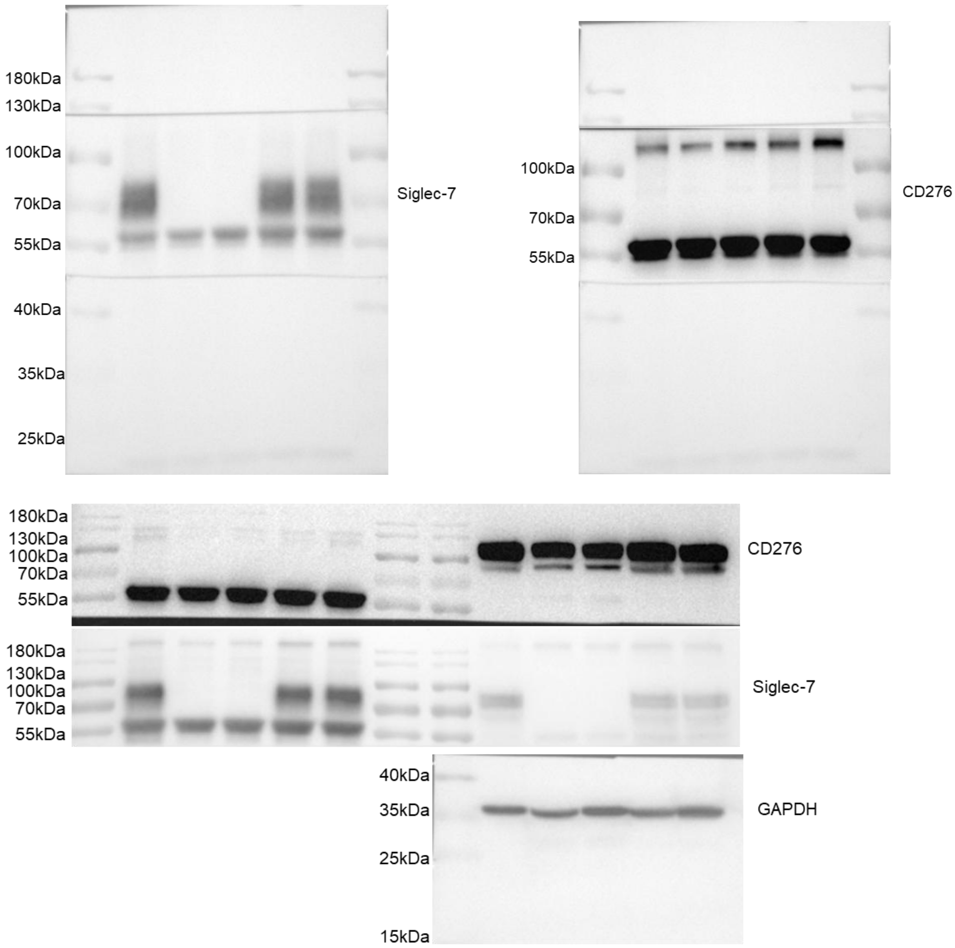

Figure 4-source data 1

Original blots of Figure 4E.

Fig. 4F

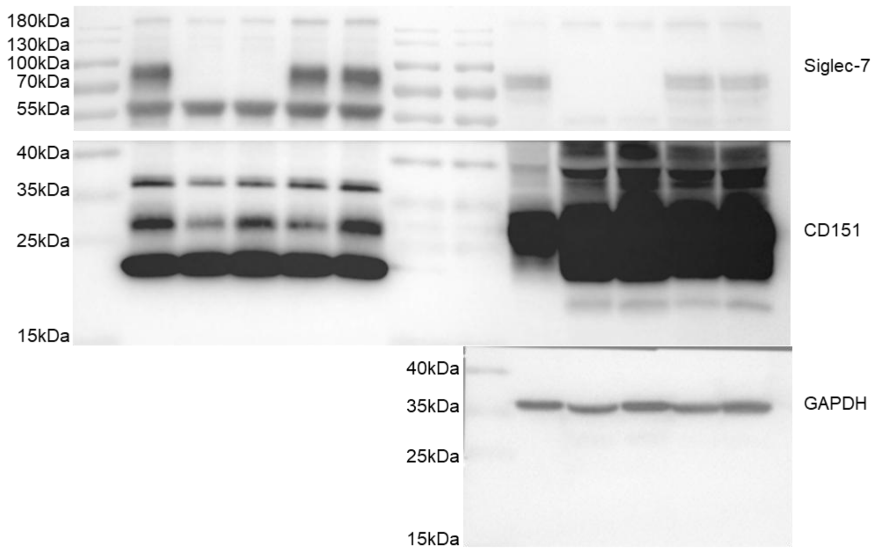

Figure 4-source data 1
Original blots of Figure 4F.

Fig. 4G

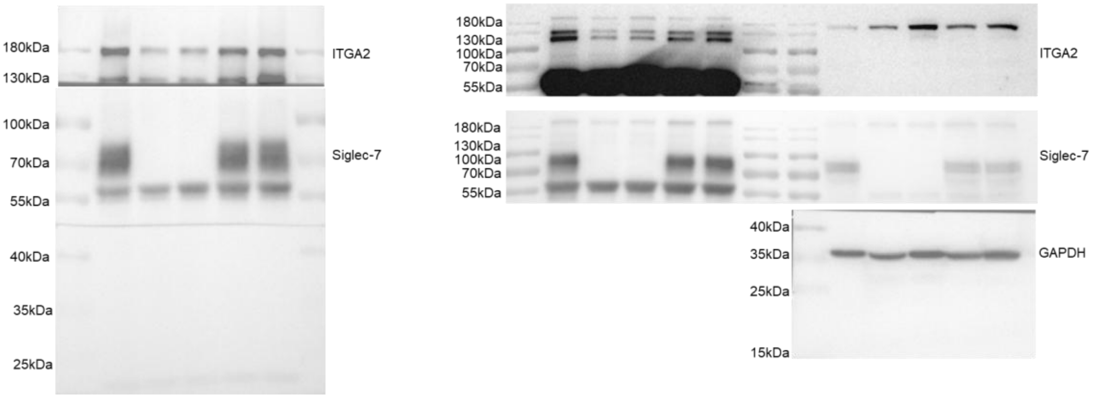

Figure 4-source data 1
Original blots of Figure 4G.

Fig. 4H

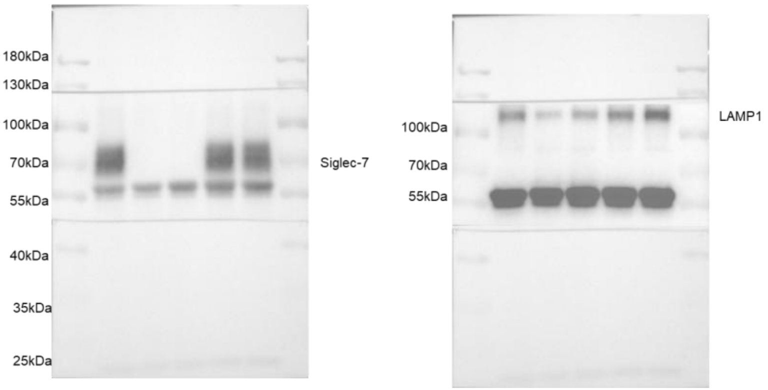

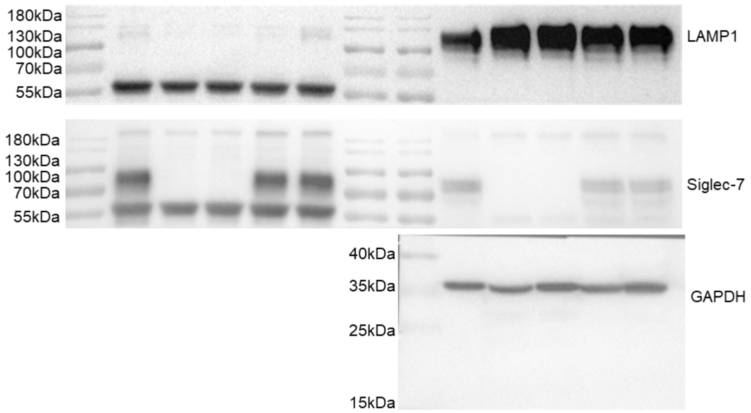

Figure 4-source data 1
Original blots of Figure 4H.

Fig. 4I-L

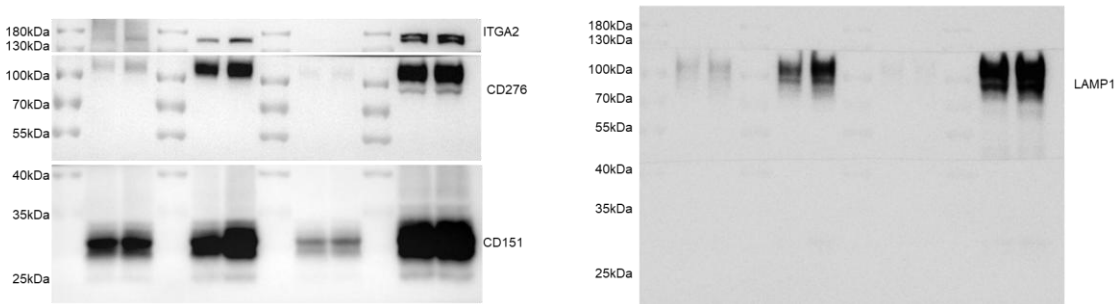

Figure 4-source data 1
Original blots of Figure 4I-L.

Fig. 4I-L

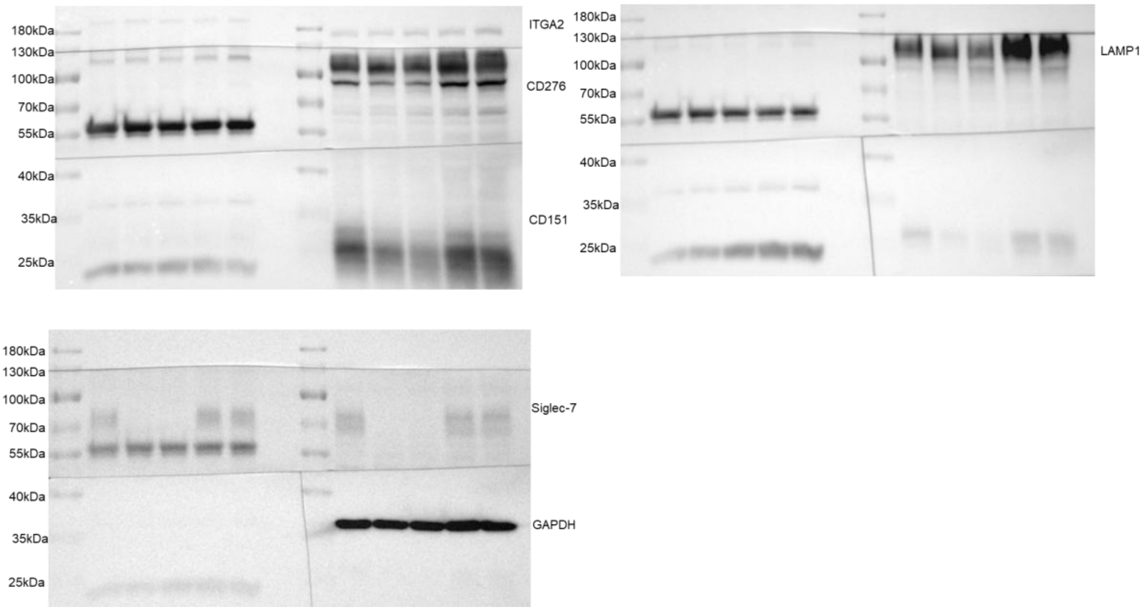

Figure 4-source data 1
Original blots of Figure 4I-L.

Fig. 6E

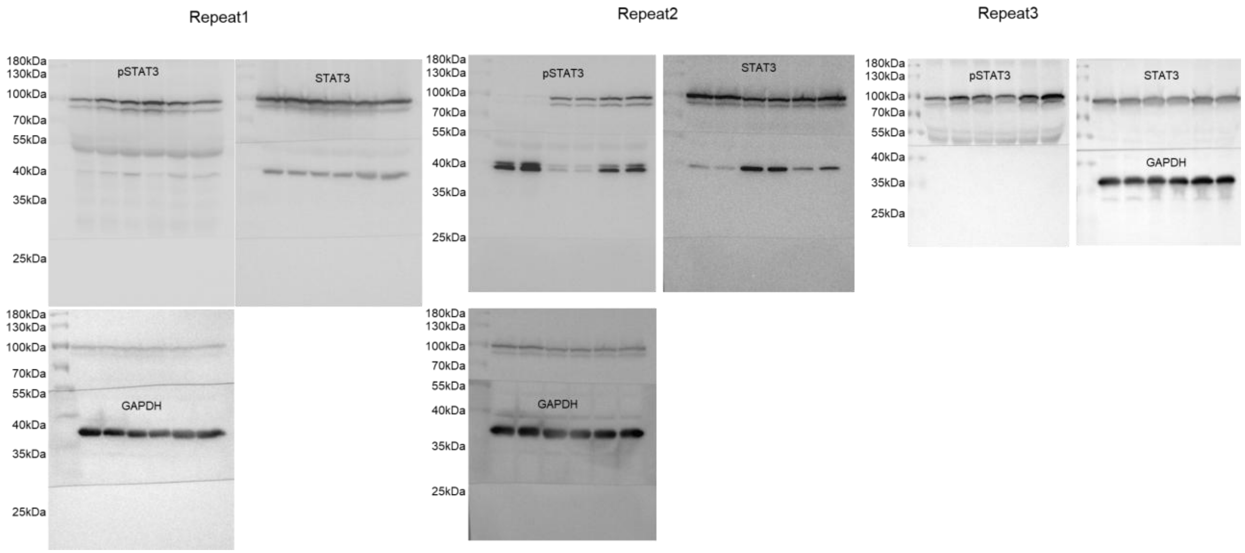

Figure 6-source data 1
Original blots of Figure 6E.

Fig. 6F

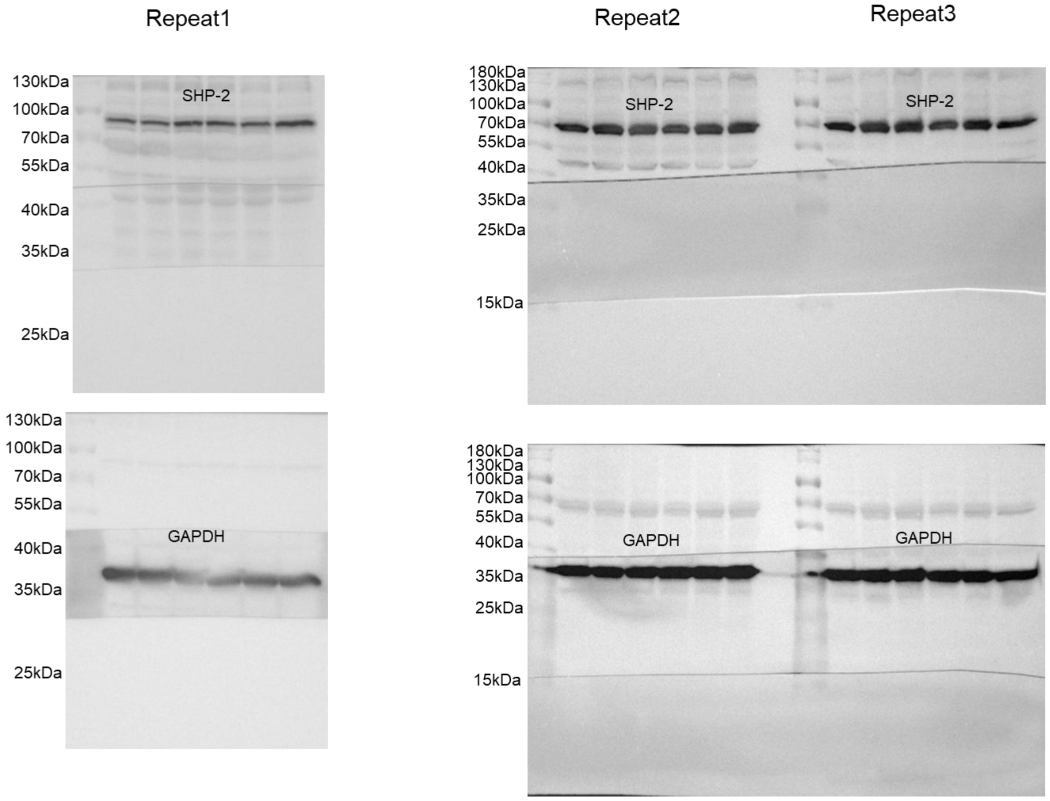

Figure 6-source data 1
Original blots of Figure 6F.

Fig. 7F

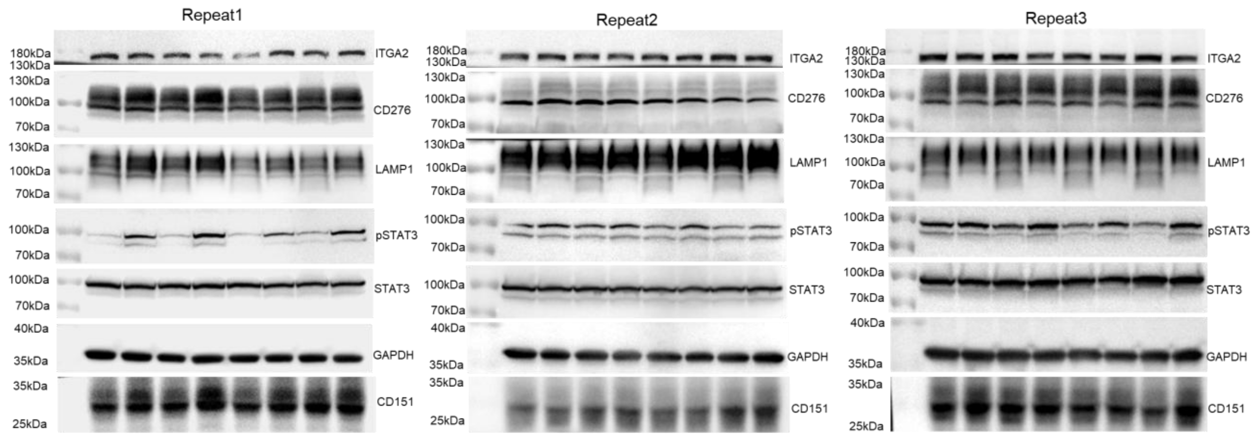

Figure 7-source data 1

Original blots of Figure 7F.

Fig. S4A

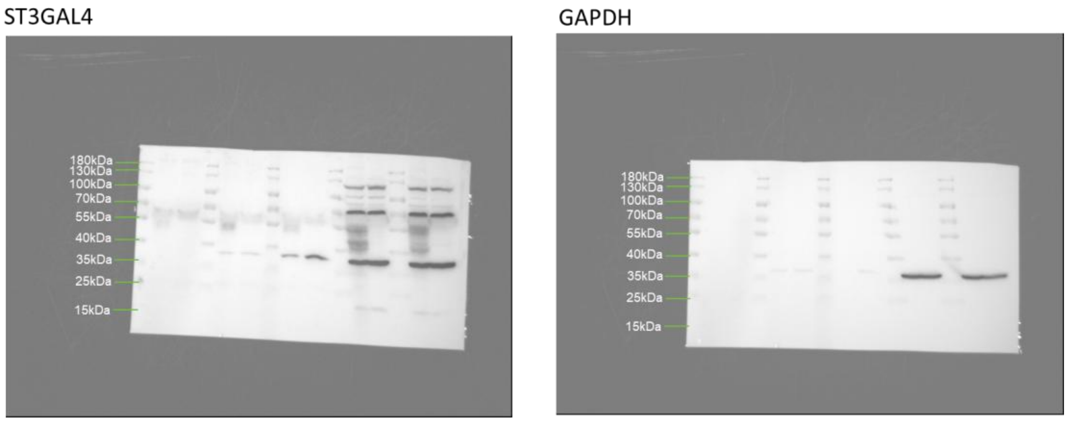

Figure S4-source data 1

Original blots of Figure S4A.

Fig. S4B

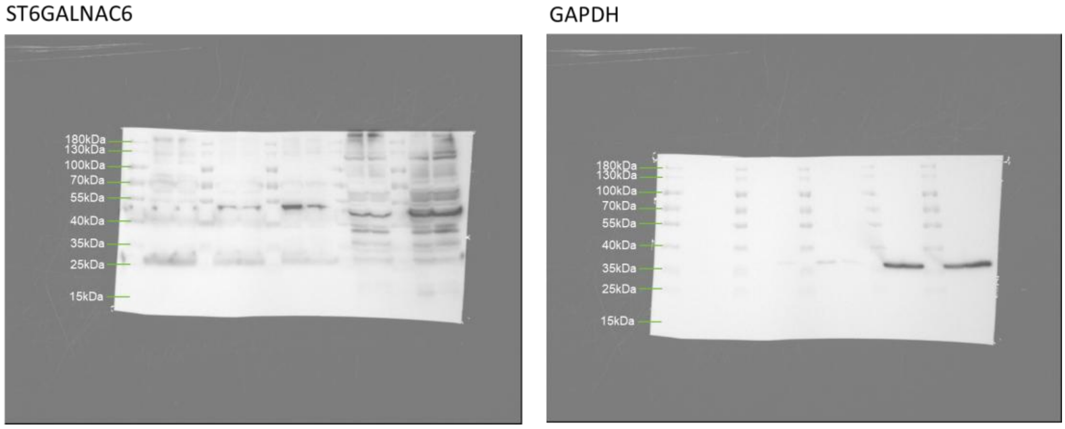

Figure S4-source data 1

Original blots of Figure S4B.

Fig. S4C

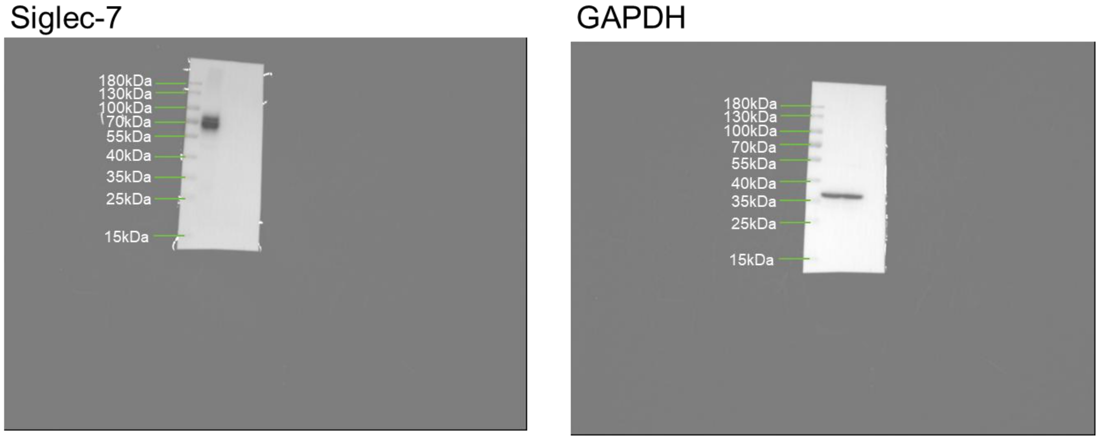

Figure S4-source data 1
Original blots of Figure S4C.

Fig. S4D-G

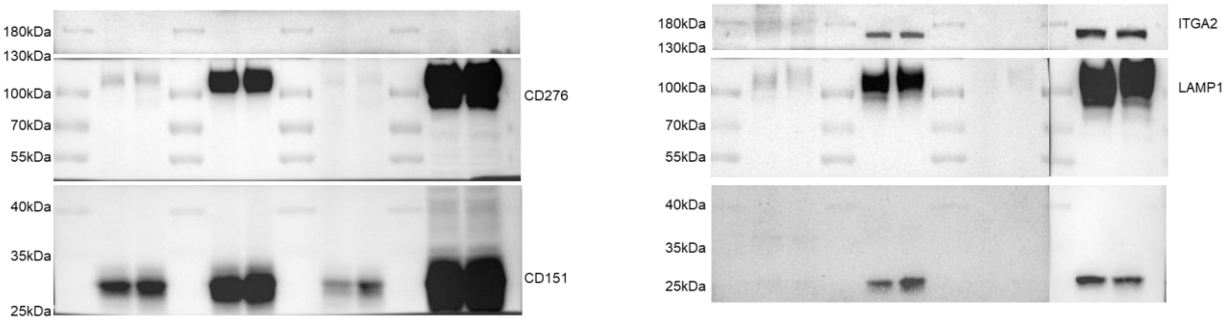

Figure S4-source data 1
Original blots of Figure S4D-G.

Fig. S4H-K

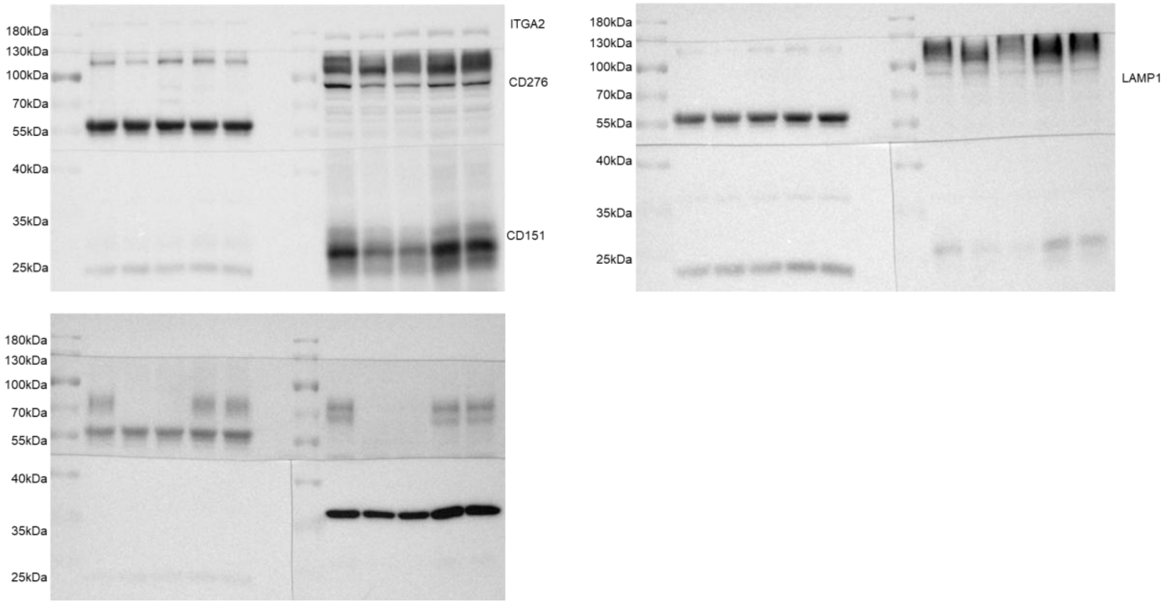

Figure S4-source data 1

Original blots of Figure S4H-K.

Fig. S7D

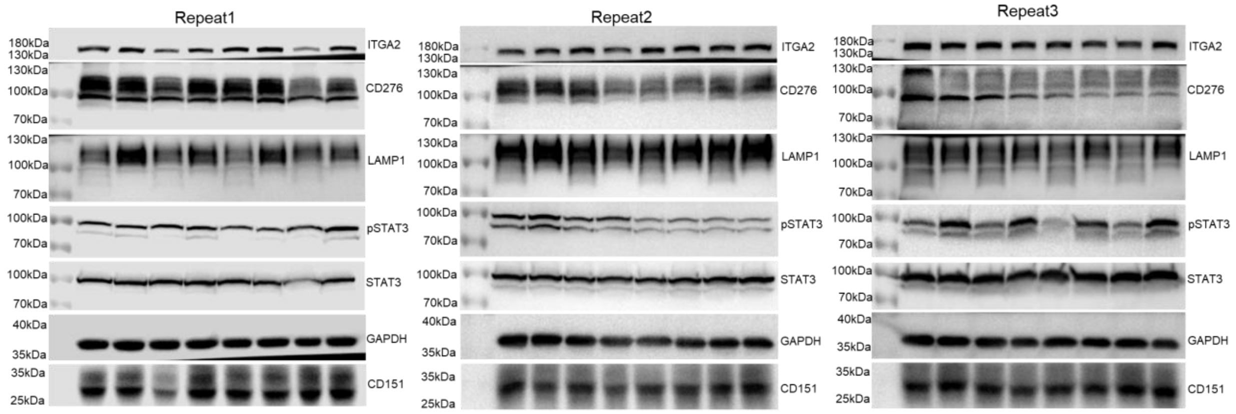

Figure S7-source data 1

Original blots of Figure S7D.

Fig. S8A-B

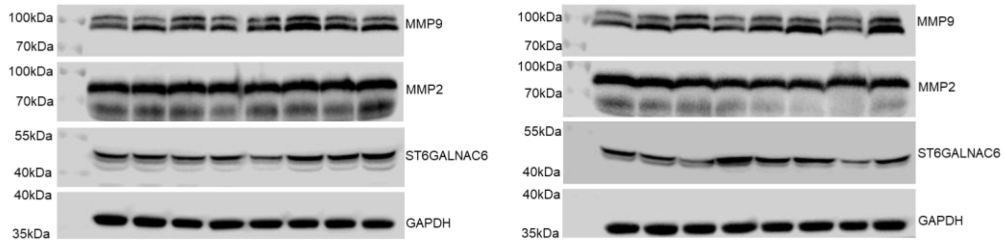

Figure S8-source data 1

Original blots of Figure S8A-B.
