# Supplementary material for: Deficient extravillous trophoblast invasion caused by impaired sialylation–Siglec-7 interaction contributes to recurrent pregnancy loss
Source: Cell Death Dis. 2026 Mar 2;17(1):291. doi: 10.1038/s41419-026-08503-9 (PMC13031383; doi:10.1038/s41419-026-08503-9)
Supplement: Supplementary file 3 — Original Western blotting image [file 41419_2026_8503_MOESM3_ESM.pdf]

Fig. 2C

ST6GALNAC6

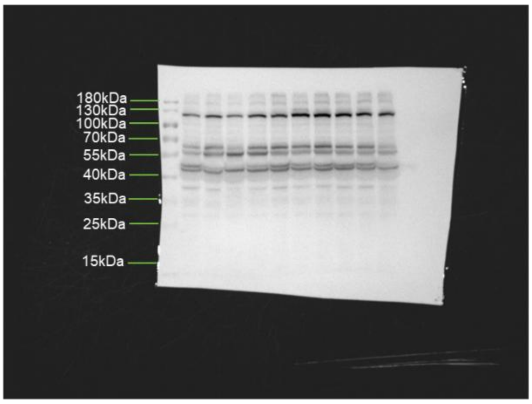

ST3GAL4

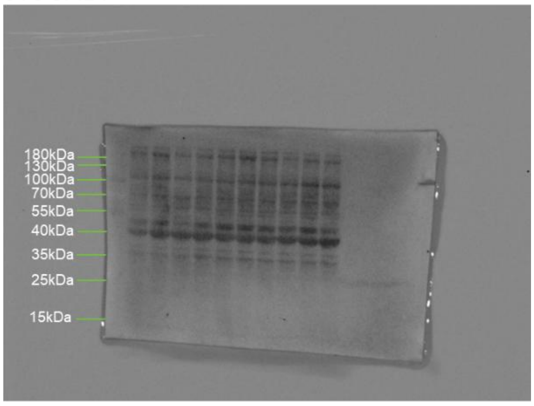

GAPDH

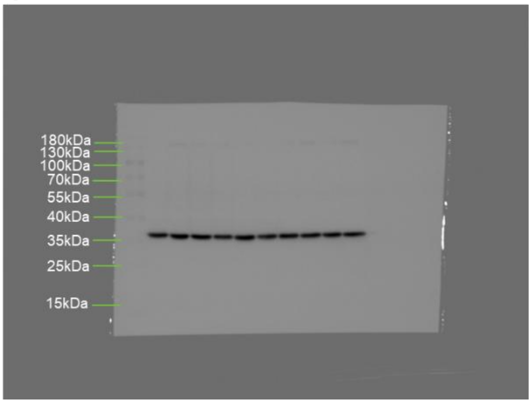

Figure 2-source data 1

Original blots of Figure 2C.

Fig. 2D

MAL II

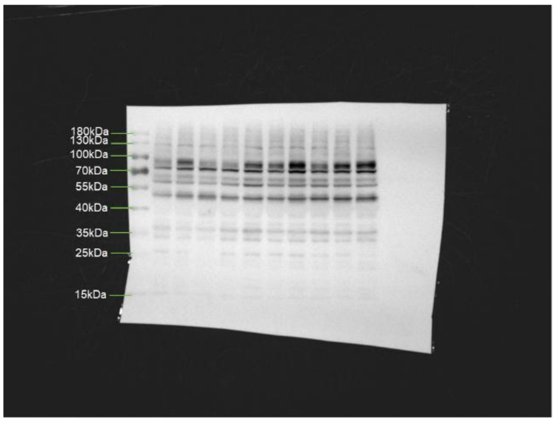

SNA

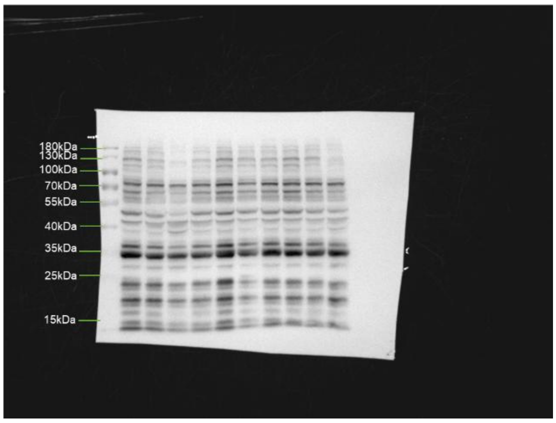

Figure 2-source data 1

Original blots of Figure 2D.

Fig. 4E

CD276

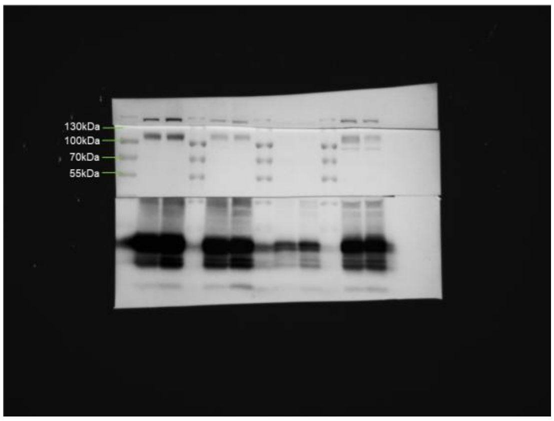

CD276

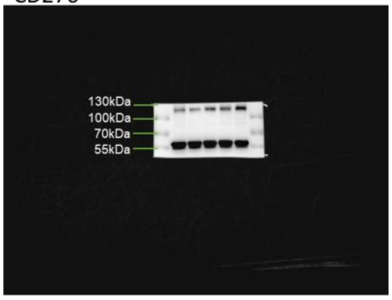

CD276

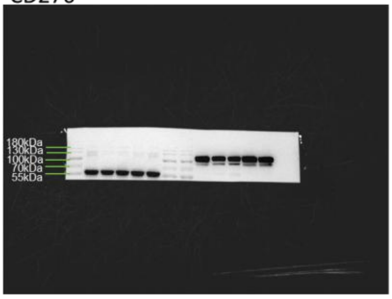

GAPDH

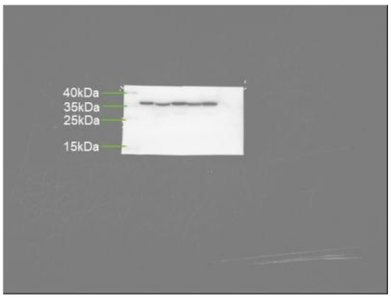

Siglec-7

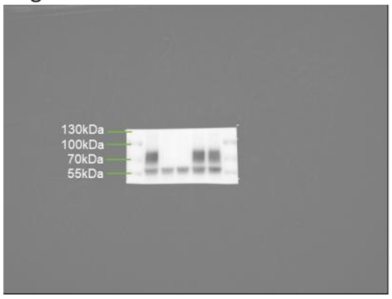

Siglec-7

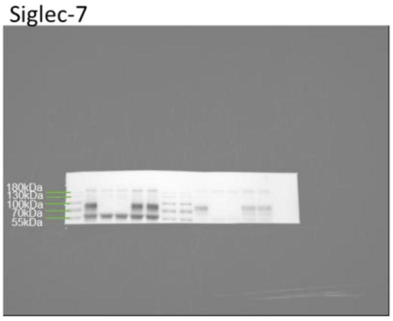

Figure 4-source data 1

Original blots of Figure 4E.

Fig. 4F

CD151

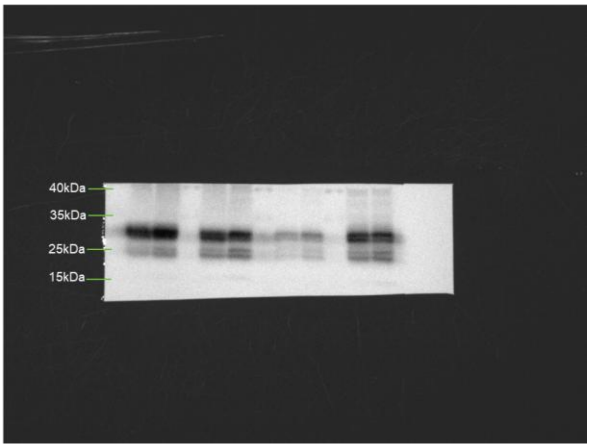

CD151

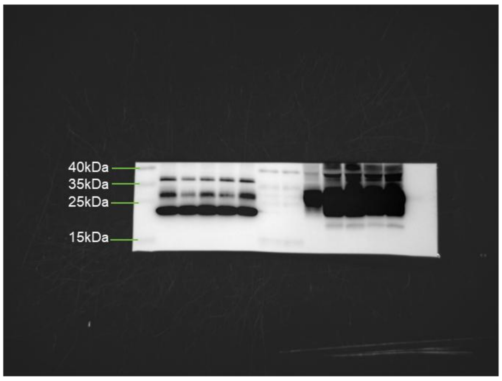

CD151

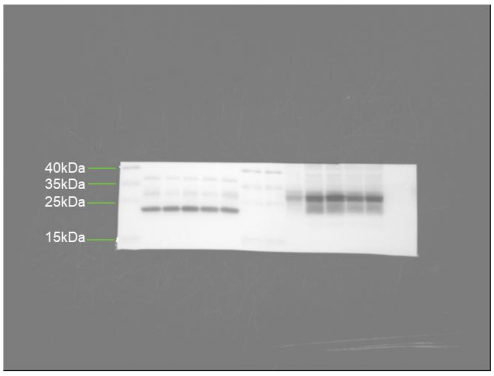

Siglec-7

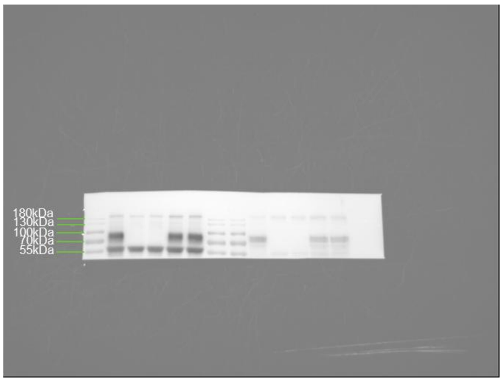

GAPDH

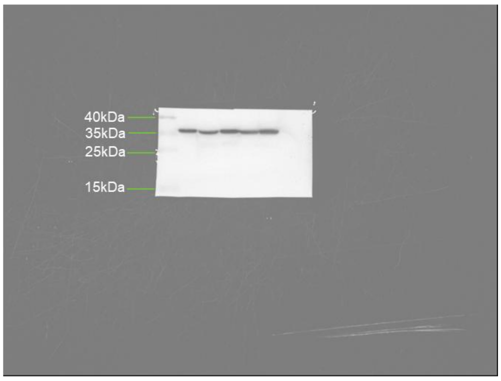

Figure 4-source data 1

Original blots of Figure 4F.

Fig. 4G

ITGA2

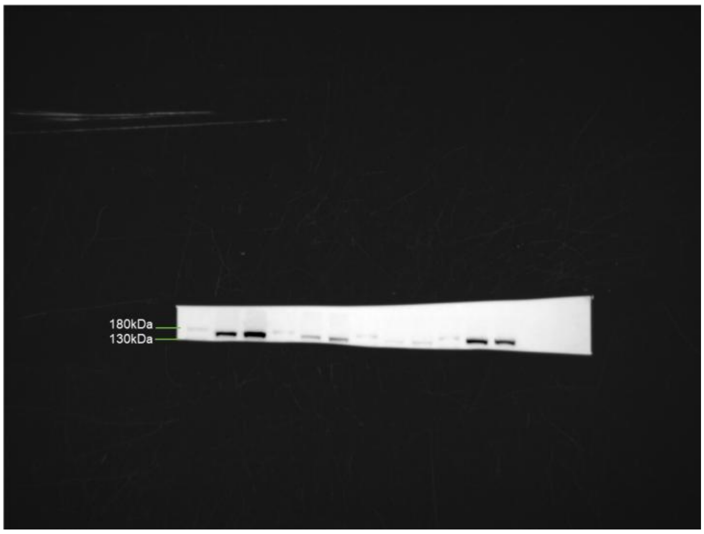

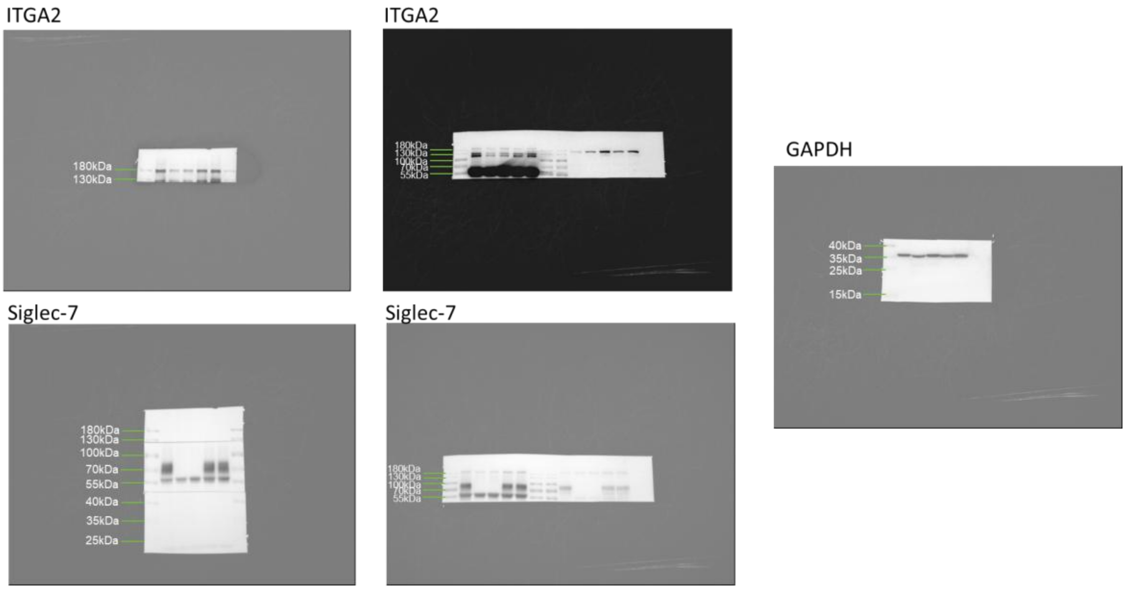

Figure 4-source data 1

Original blots of Figure 4G.

Fig. 4H

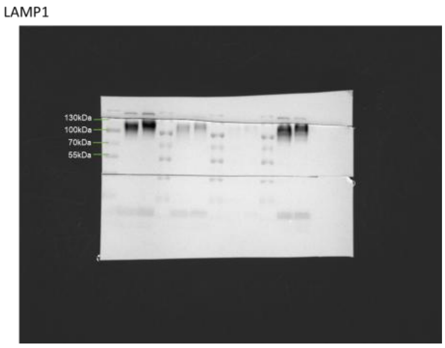

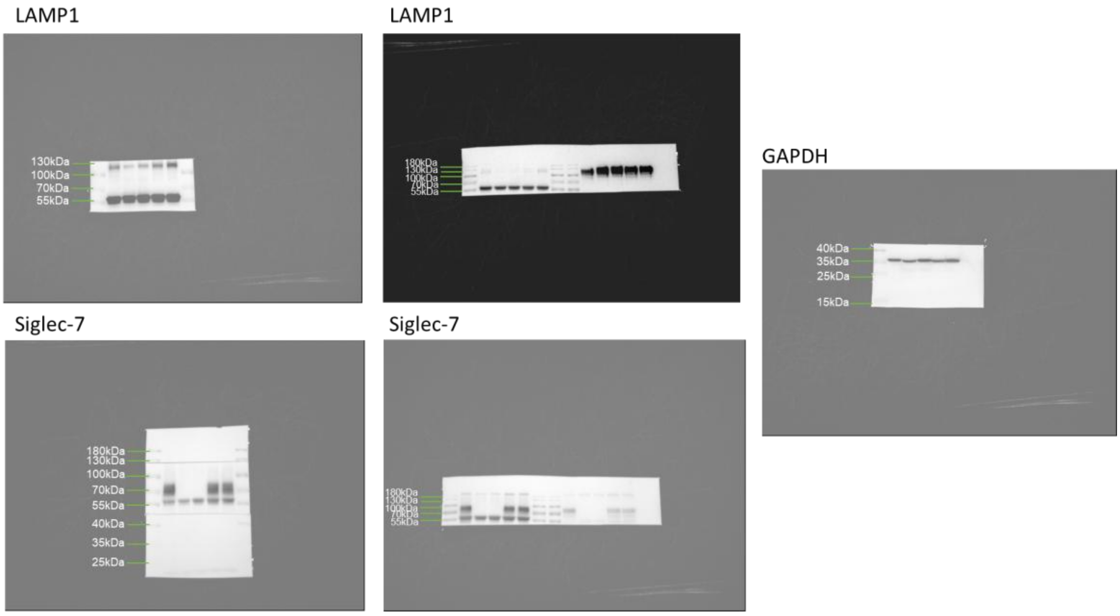

Figure 4-source data 1

Original blots of Figure 4H.

Fig. 4I

CD276

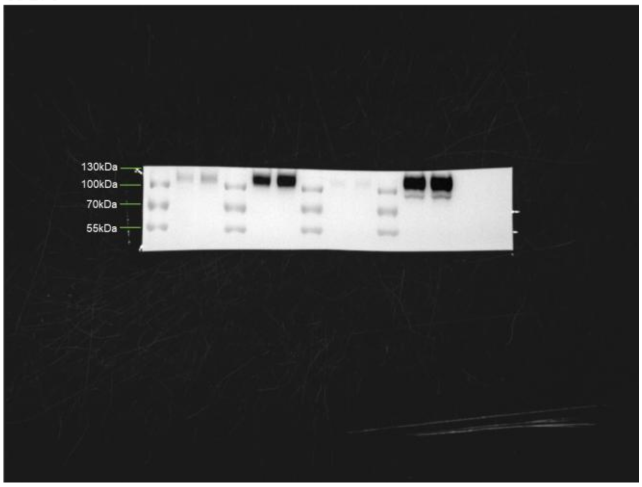

CD276

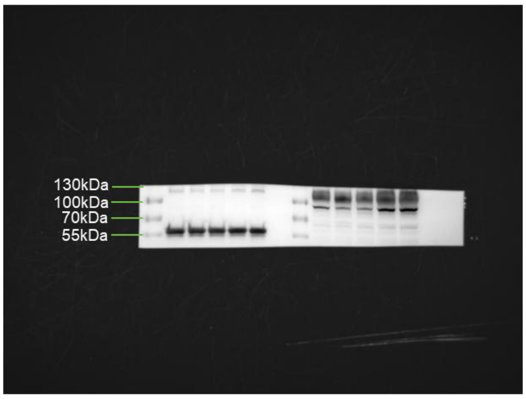

Siglec-7

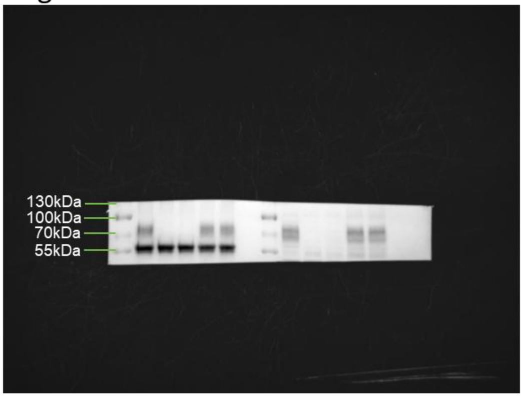

Long exposure time of CD276

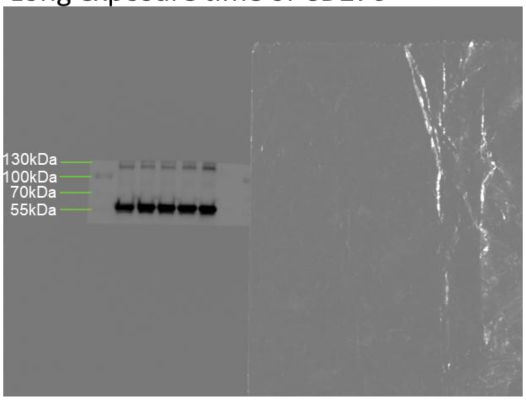

GAPDH

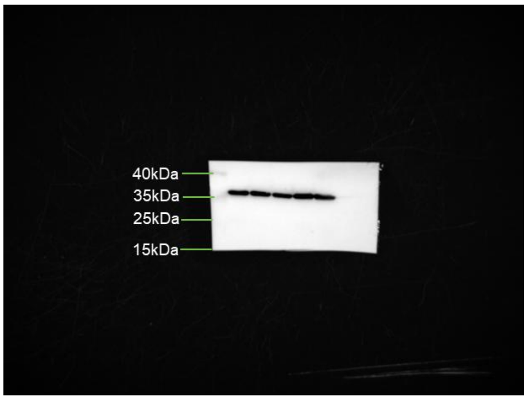

Figure 4-source data 1

Original blots of Figure 4I.

Fig. 4J

CD151

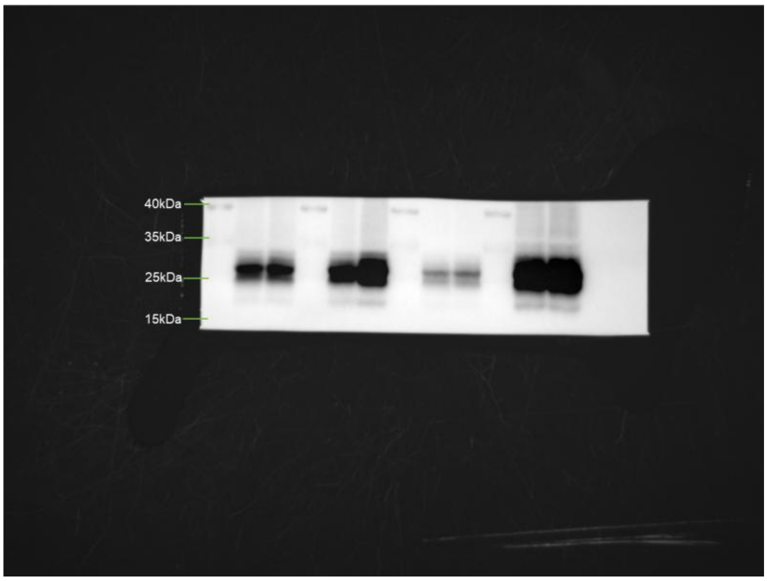

CD151

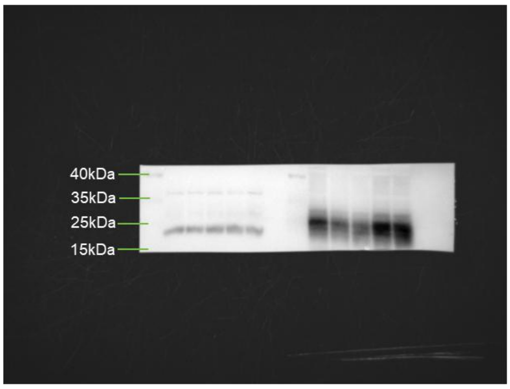

Siglec-7

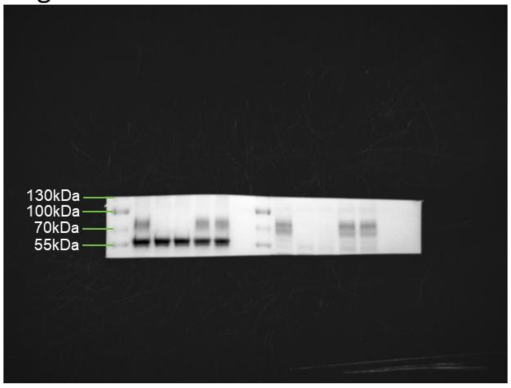

Long exposure time of CD151

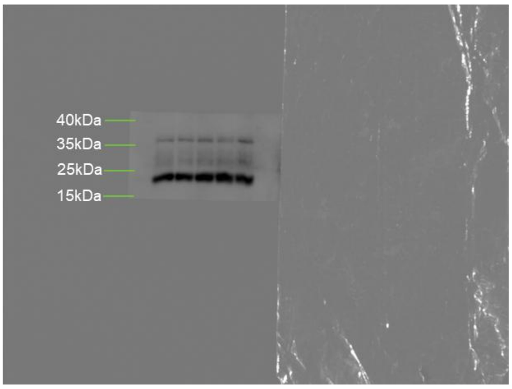

GAPDH

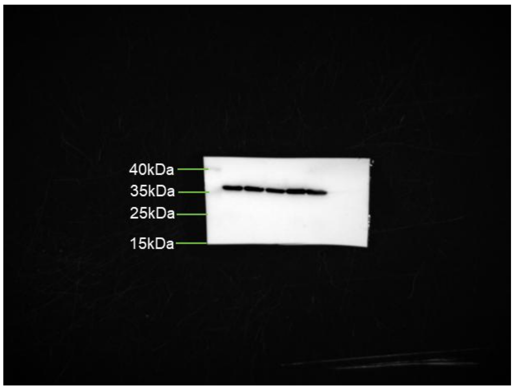

Figure 4-source data 1

Original blots of Figure 4J.

Fig. 4K

ITGA2

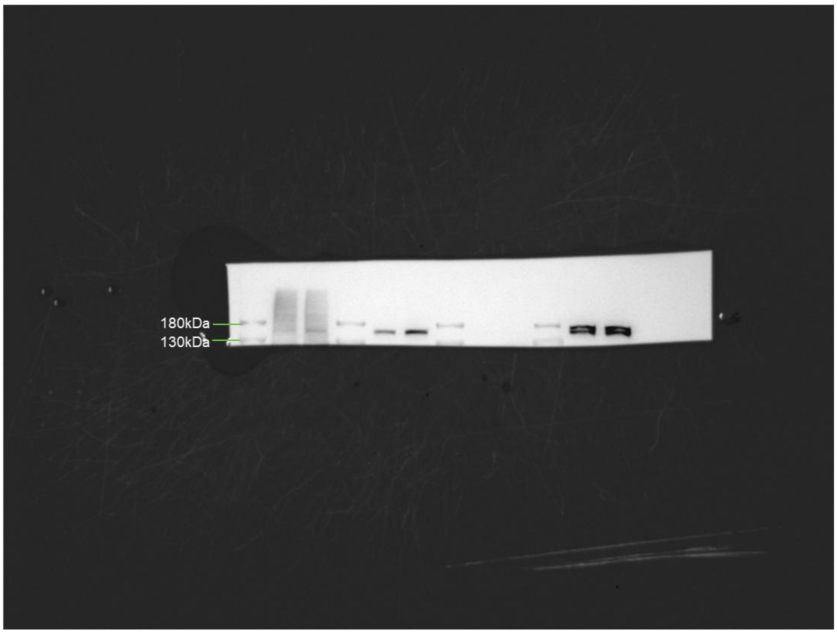

ITGA2

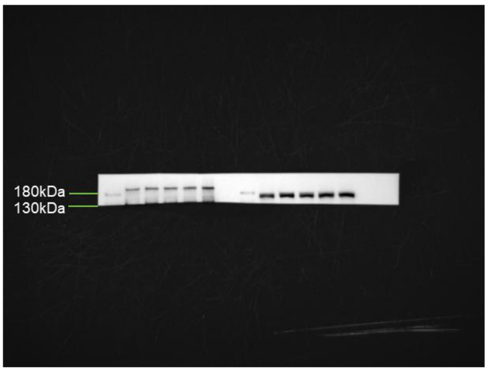

Siglec-7

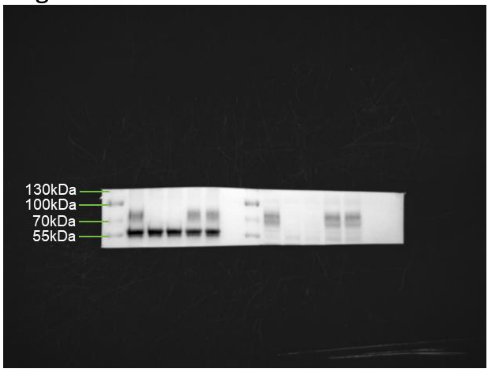

GAPDH

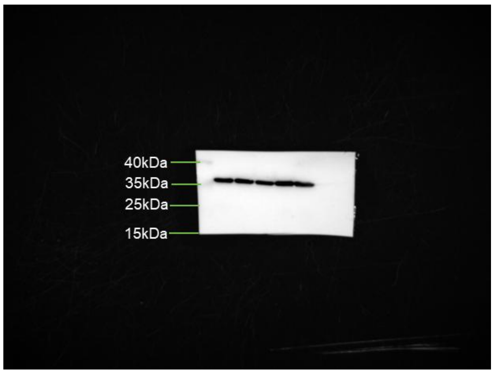

Figure 4-source data 1
Original blots of Figure 4K.

Fig. 4L

LAMP1

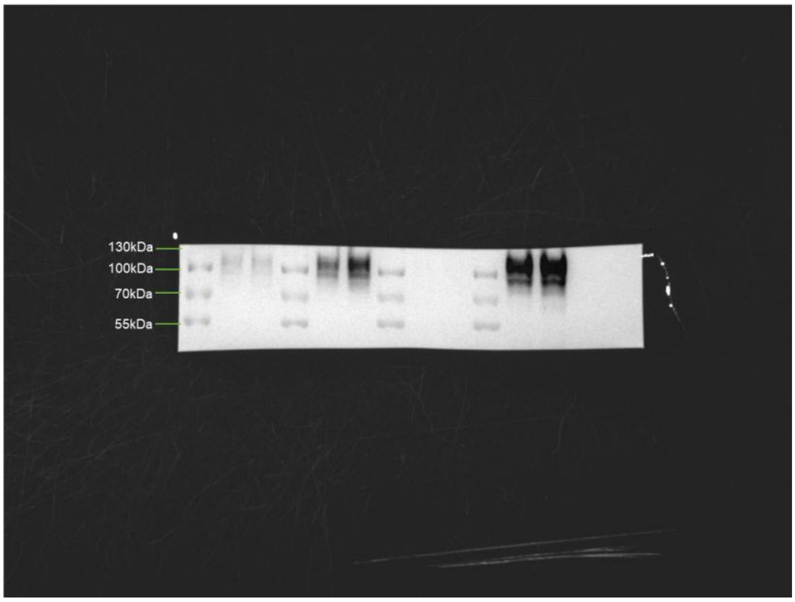

Fig. 4L

LAMP1

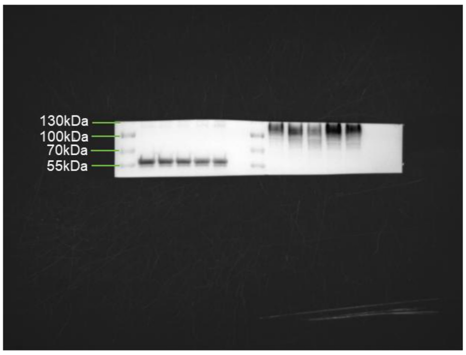

Siglec-7

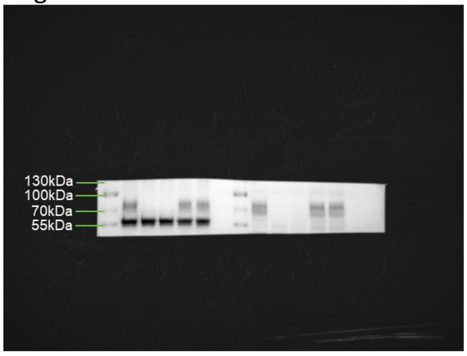

Long exposure time of LAMP1

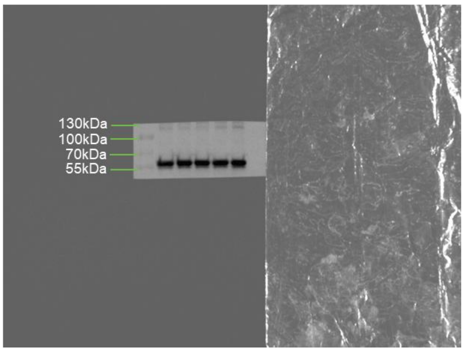

GAPDH

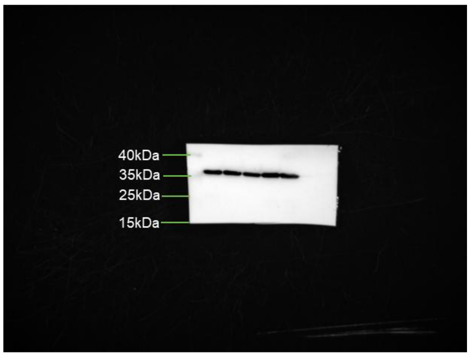

Figure 4-source data 1
Original blots of Figure 4L.

Fig. 6E

1-pSTAT3

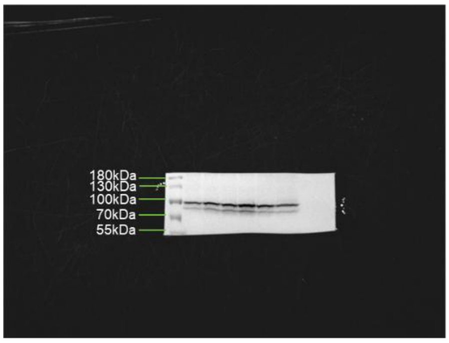

GAPDH

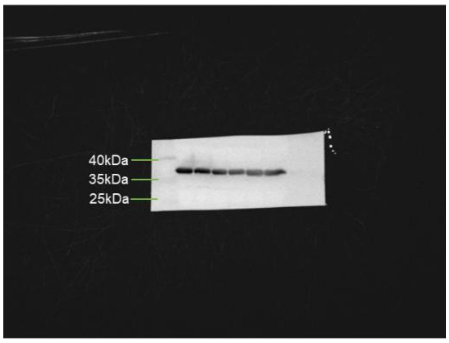

1-STAT3

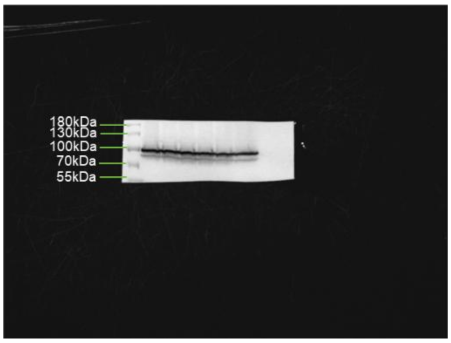

Figure 6-source data 1

Original blots of Figure 6E.

Fig. 6E

2-pSTAT3

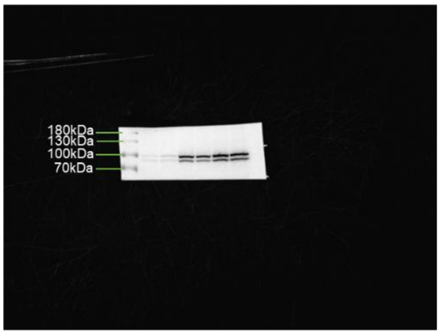

GAPDH

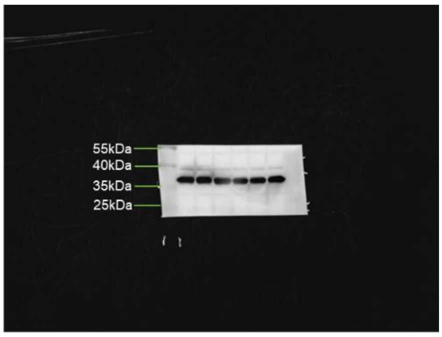

2-STAT3

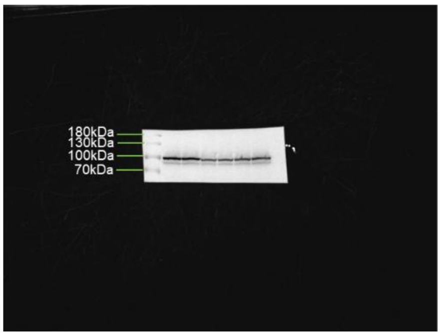

Figure 6-source data 2

Original blots of Figure 6E.

Fig. 6E

3-pSTAT3

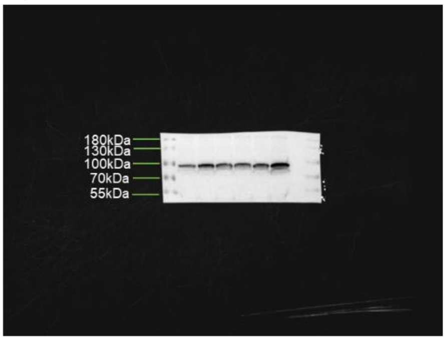

3-STAT3

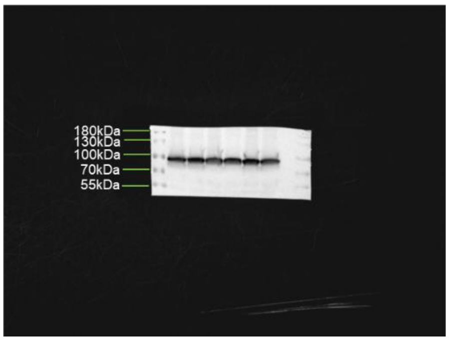

GAPDH

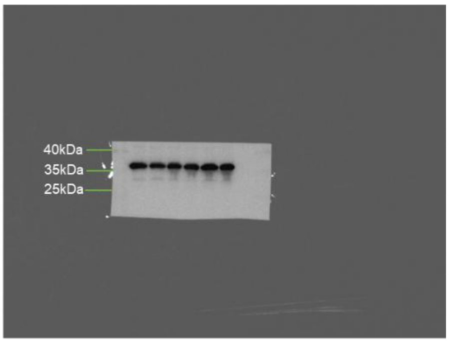

Figure 6-source data 3
Original blots of Figure 6E.

Fig. 6F

1-SHP-2

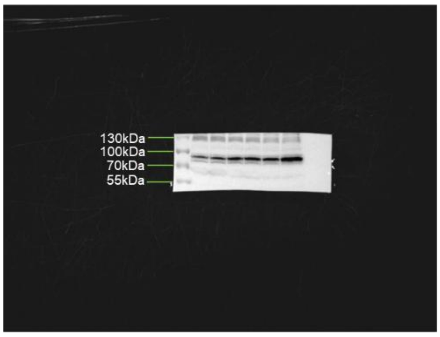

2-SHP-2 3-SHP-2

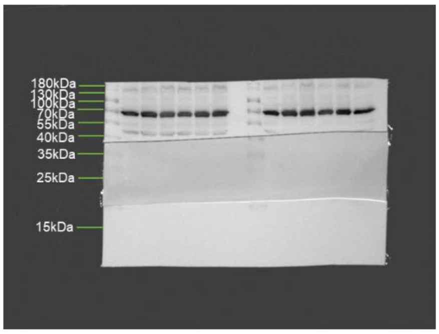

GAPDH

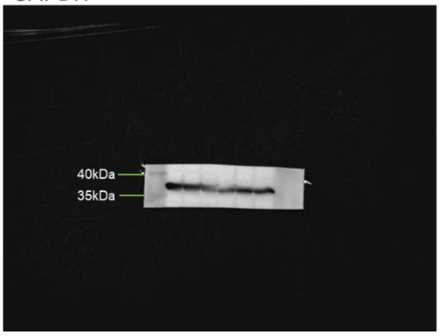

GAPDH

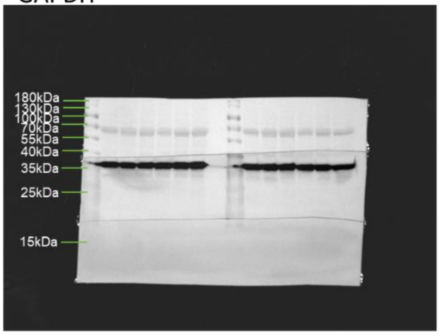

Figure 6-source data 1-3
Original blots of Figure 6F.

Fig. 7F

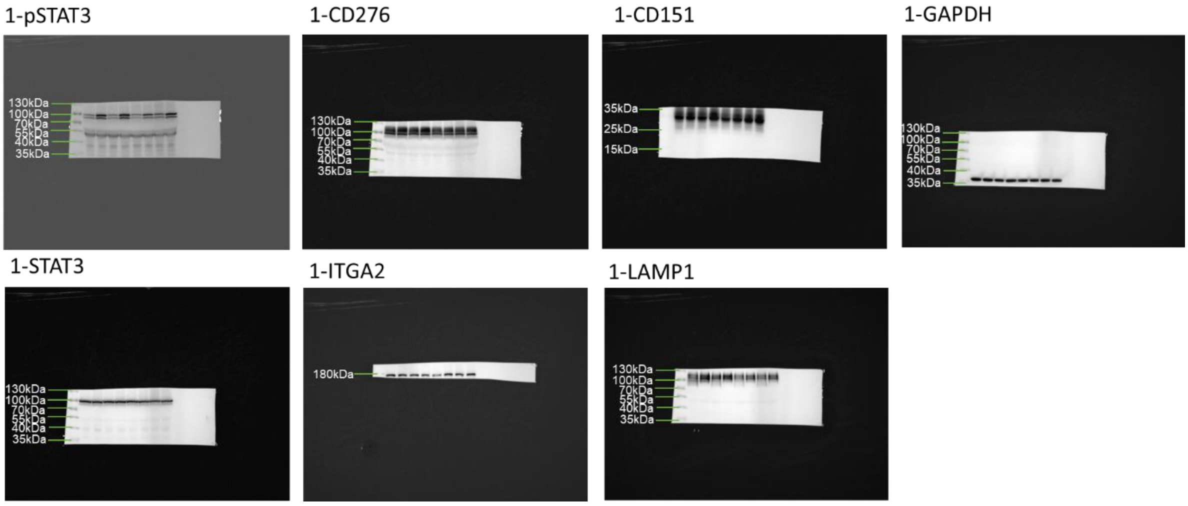

Figure 7-source data 1
Original blots of Figure 7F.

Fig. 7F

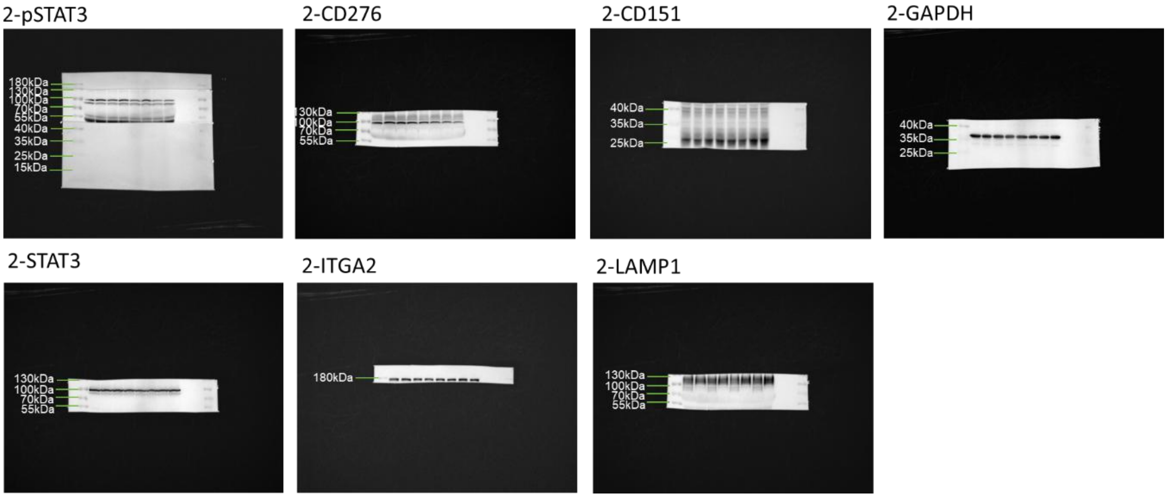

Figure 7-source data 2
Original blots of Figure 7F.

Fig. 7F

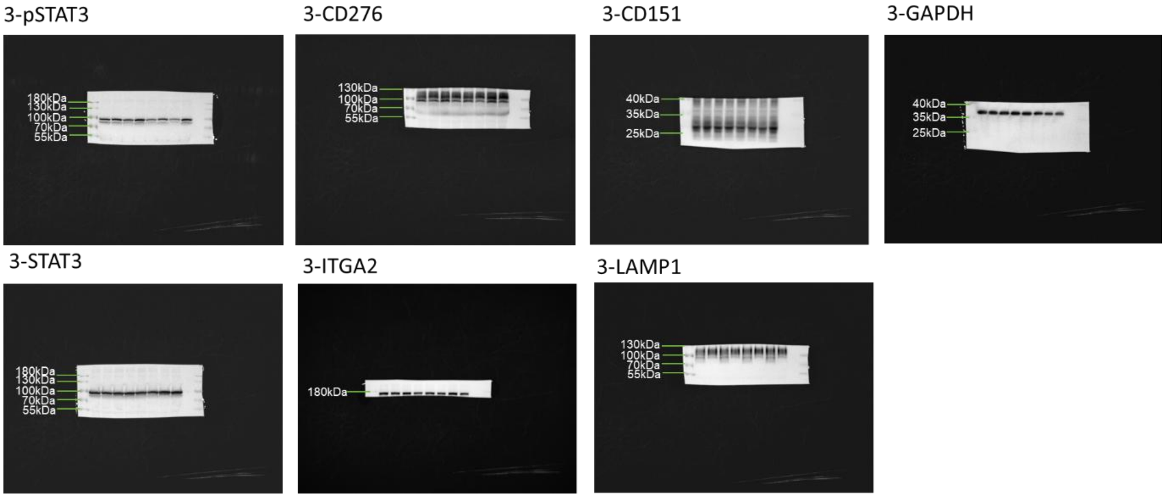

Figure 7-source data 3

Original blots of Figure 7F.

Fig. S4A

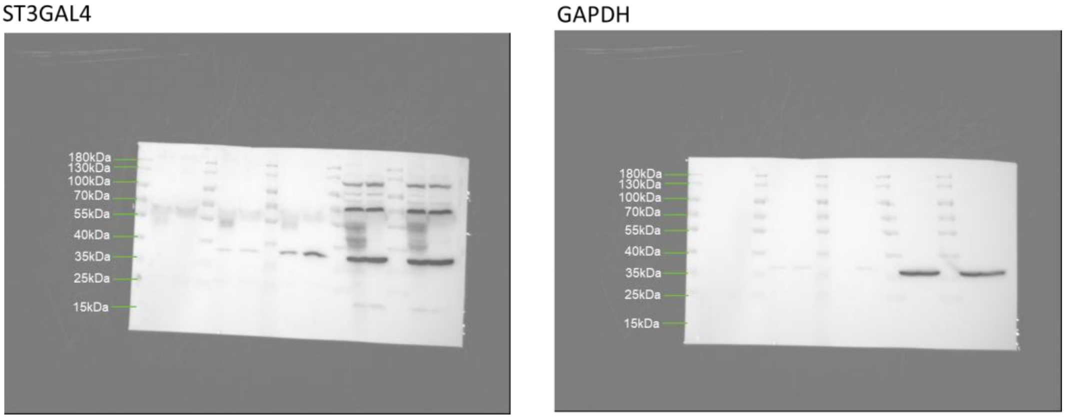

Figure S4-source data 1

Original blots of Figure S4A.

Fig. S4B

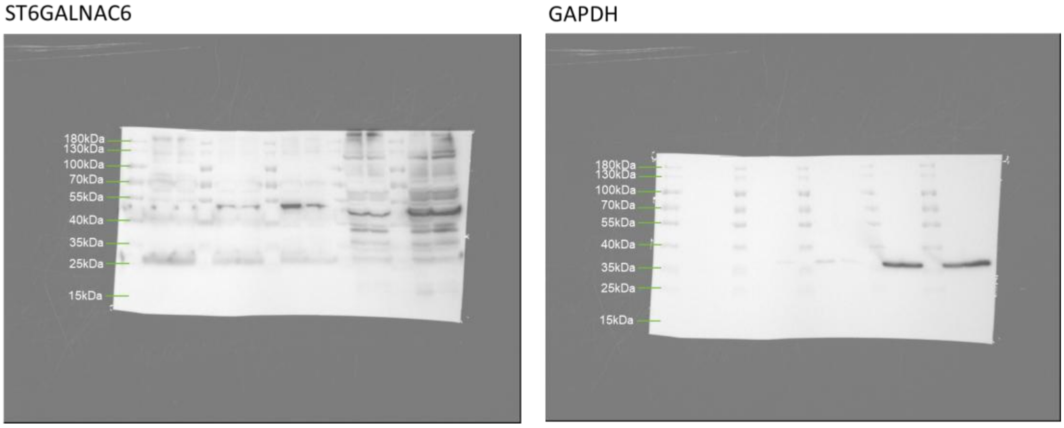

Figure S4-source data 1

Original blots of Figure S4B.

Fig. S4C

Siglec-7

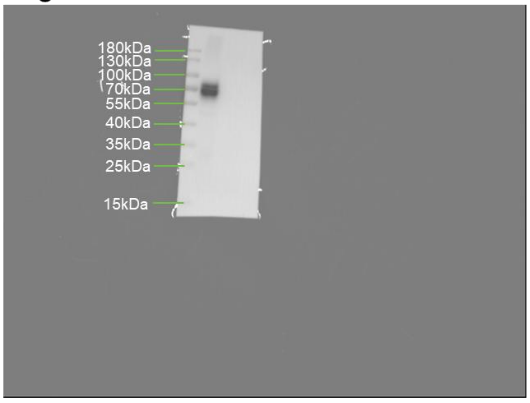

GAPDH

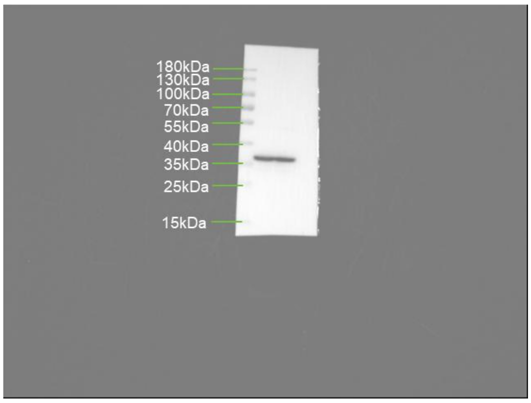

Figure S4-source data 1

Original blots of Figure S4C.

Fig. S4D

CD276

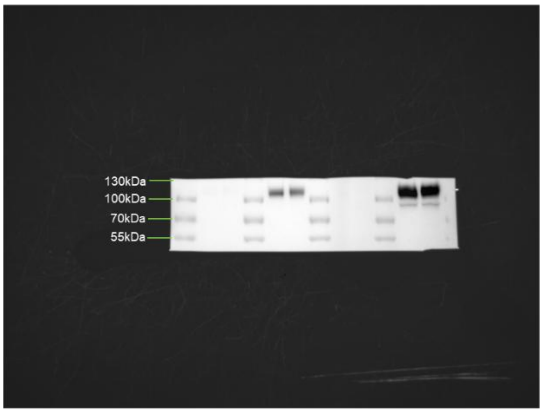

Long exposure time of CD276

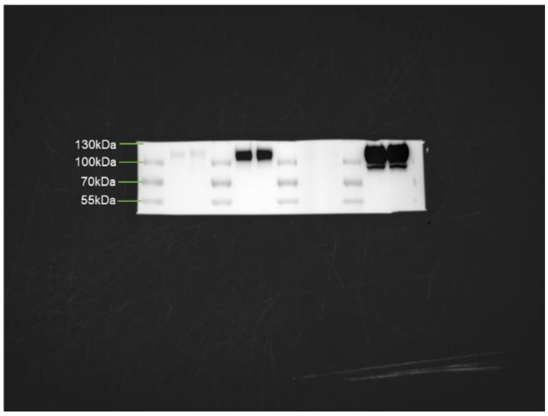

Figure S4-source data 1

Original blots of Figure S4D.

Fig. S4E

CD151

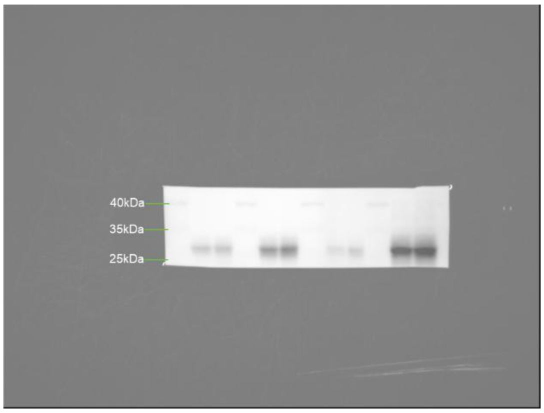

Long exposure time of CD151

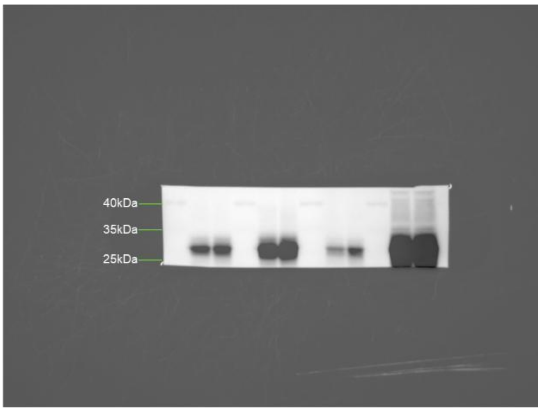

Figure S4-source data 1

Original blots of Figure S4E.

Fig. S4F

ITGA2

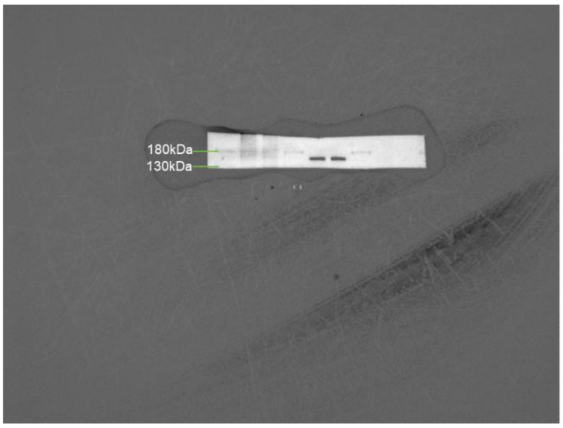

ITGA2

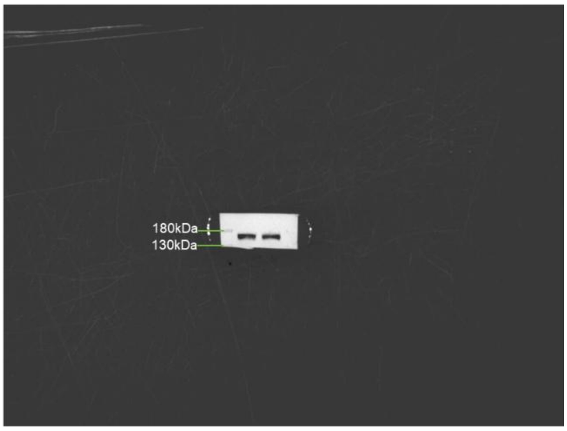

Figure S4-source data 1
Original blots of Figure S4F.

Fig. S4G

LAMP1

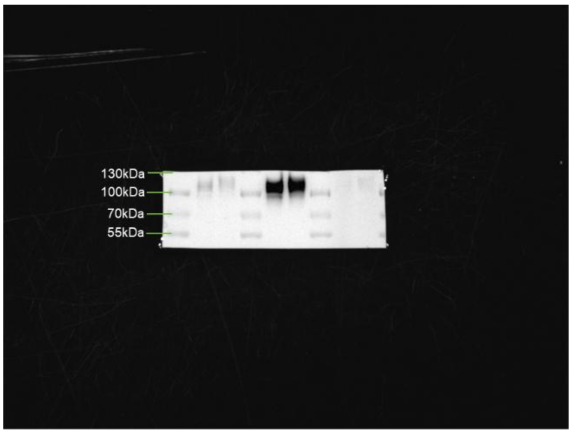

LAMP1

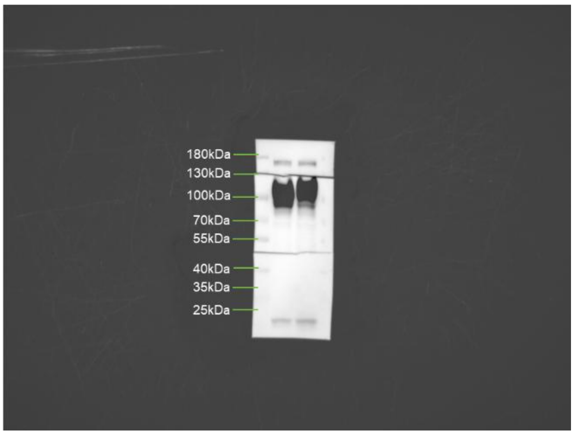

Figure S4-source data 1
Original blots of Figure S4G.

Fig. S4H

CD276

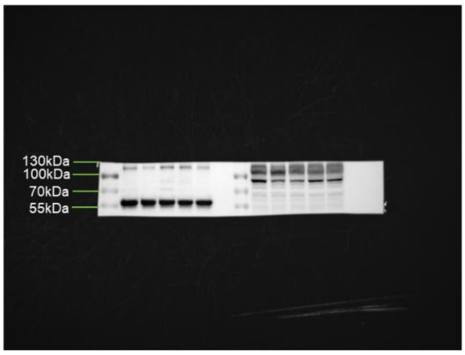

Siglec-7

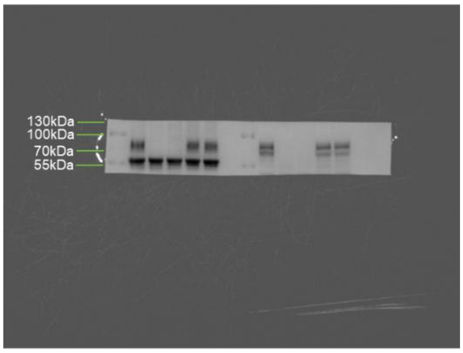

GAPDH

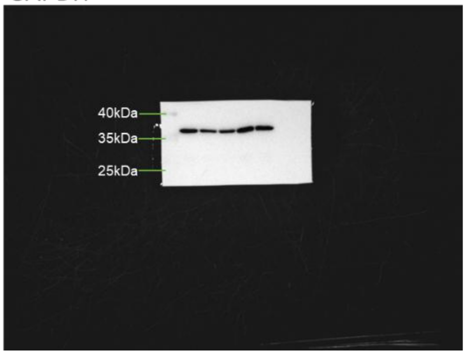

Figure S4-source data 1
Original blots of Figure S4H.

Fig. S4I

CD151

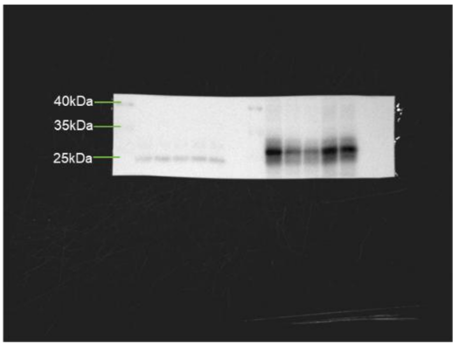

Siglec-7

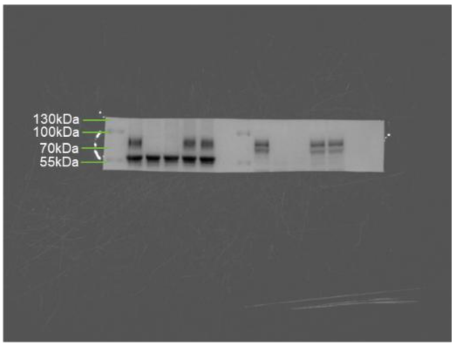

Long exposure time of CD151

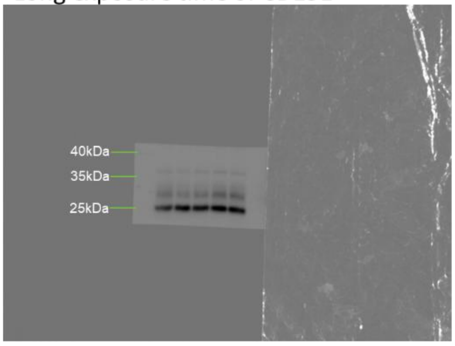

GAPDH

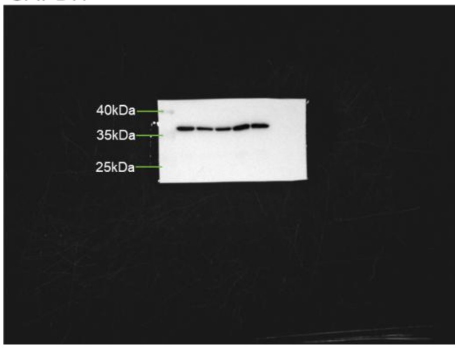

Figure S4-source data 1
Original blots of Figure S4I.

Fig. S4J

ITGA2

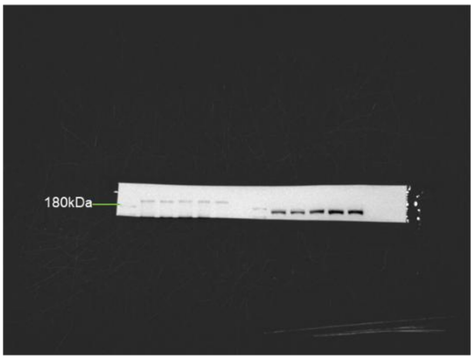

Siglec-7

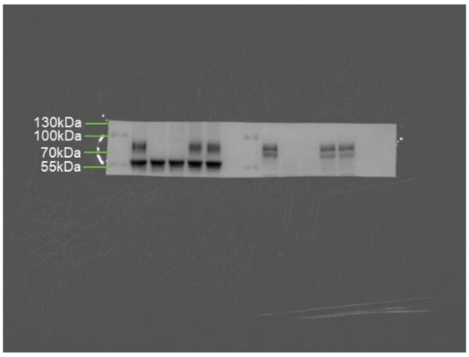

Long exposure time of ITGA2

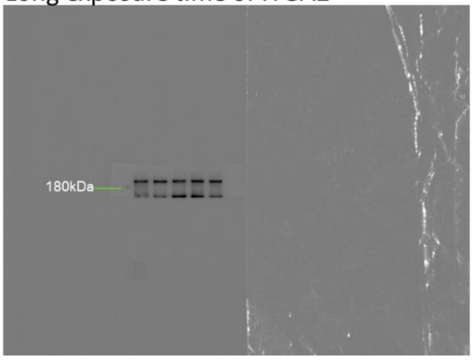

GAPDH

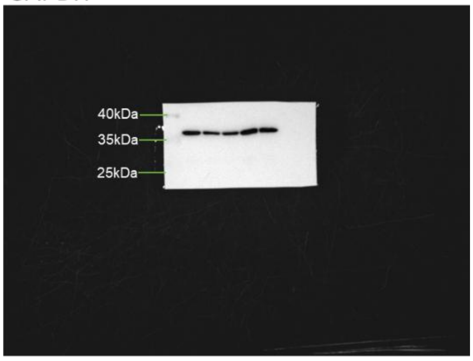

Figure S4-source data 1

Original blots of Figure S4J.

Fig. S4K

LAMP1

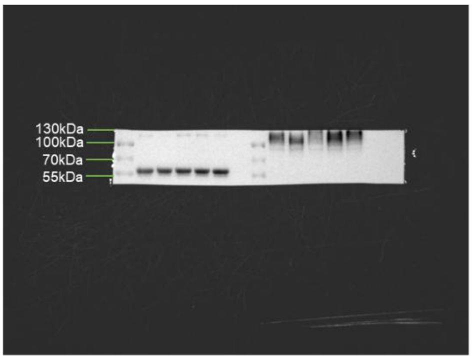

Siglec-7

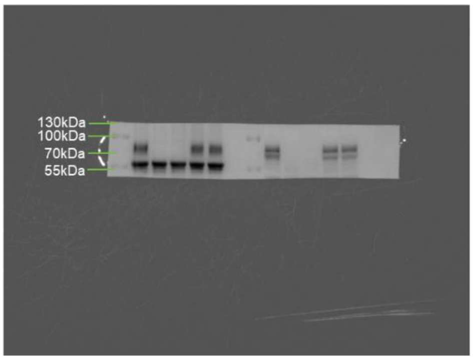

Long exposure time of LAMP1

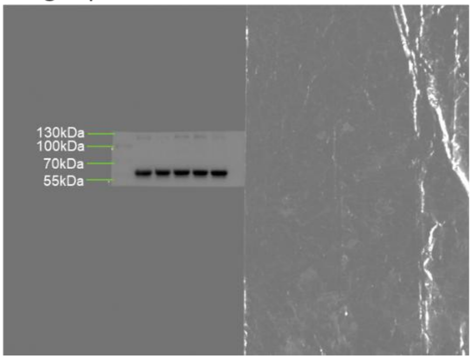

GAPDH

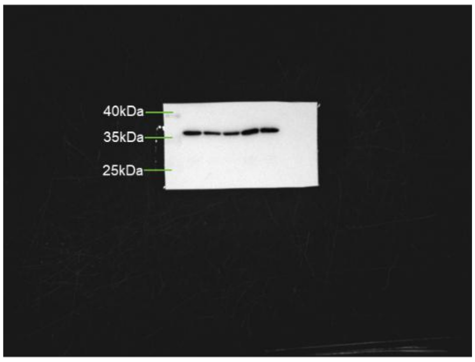

Figure S4-source data 1

Original blots of Figure S4K.

Fig. S7D

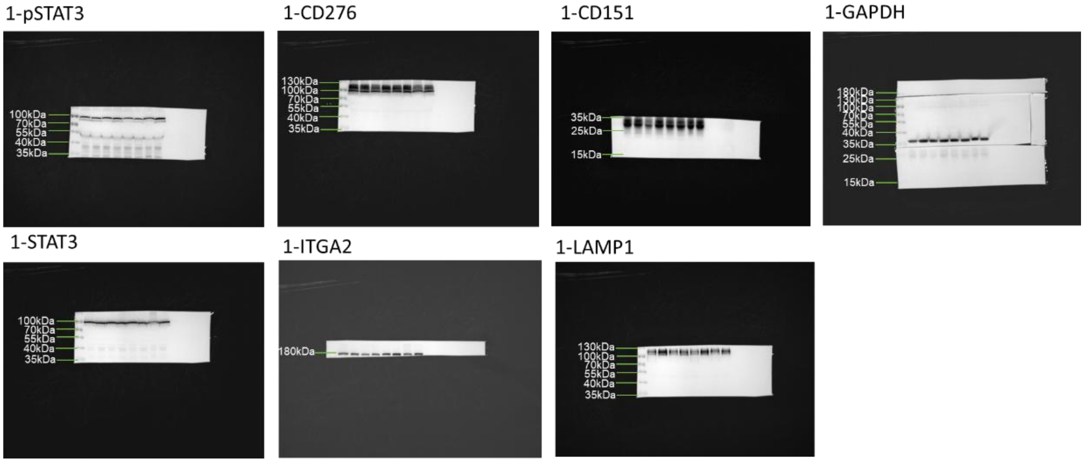

Figure S7-source data 1

Original blots of Figure S7D.

Fig. S7D

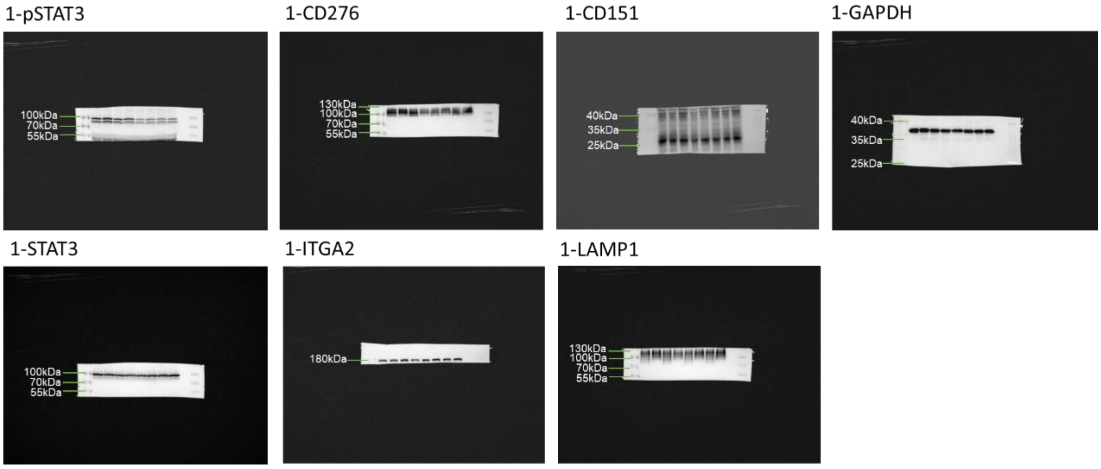

Figure S7-source data 2

Original blots of Figure S7D.

Fig. S7D

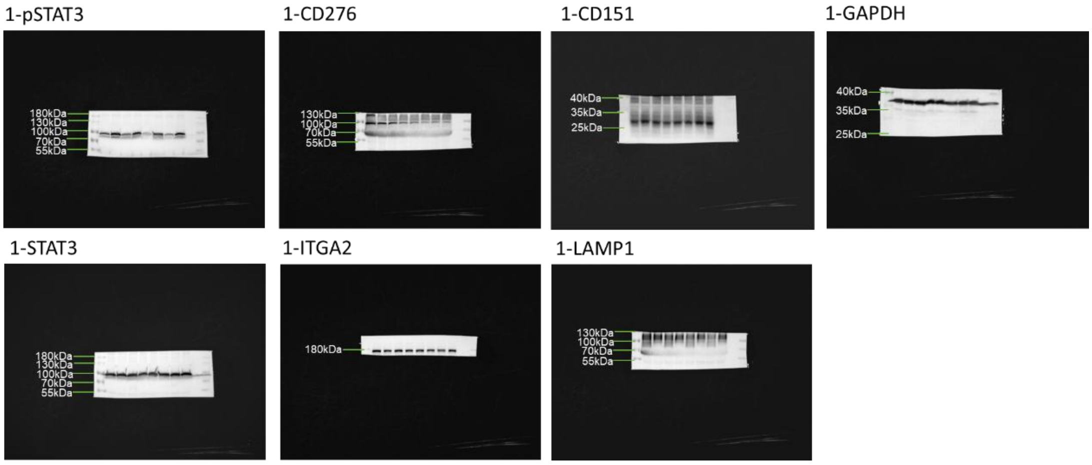

Figure S7-source data 3

Original blots of Figure S7D.

Fig. S8A

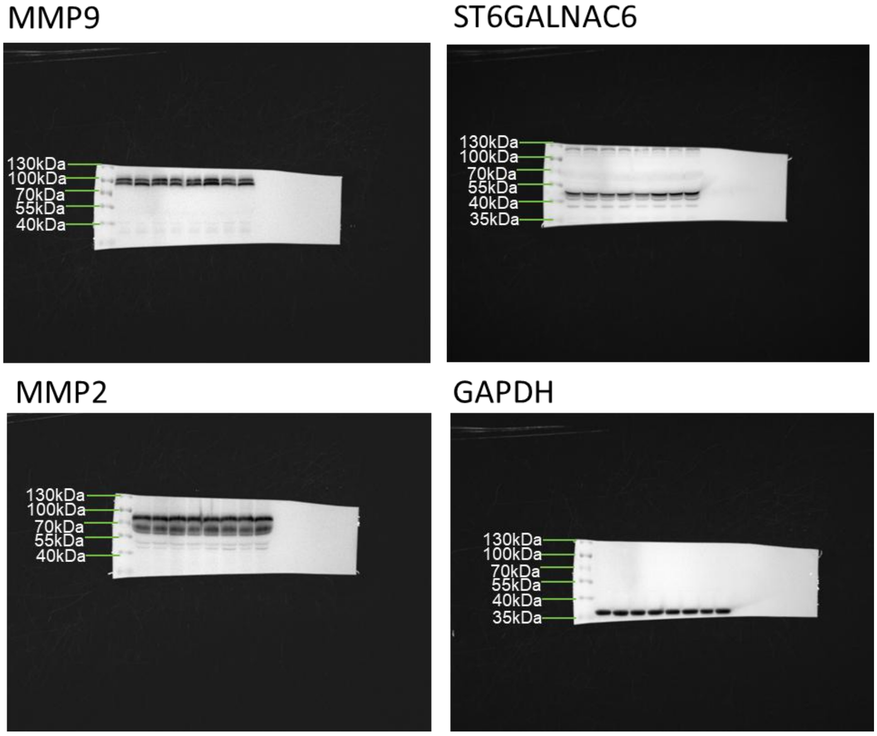

Figure S8-source data 1

Original blots of Figure S8A.

Fig. S8B

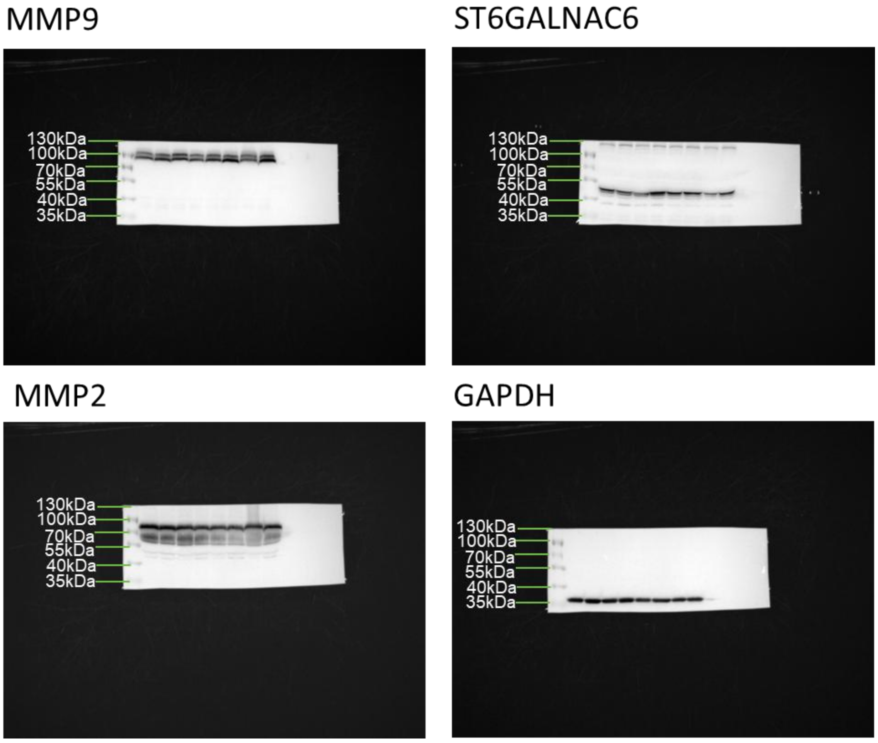

Figure S8-source data 1

Original blots of Figure S8B.
